# Distinguishing between driver and passenger mutations in individual cancer genomes by network enrichment analysis

**DOI:** 10.1186/1471-2105-15-308

**Published:** 2014-09-19

**Authors:** Simon Kebede Merid, Daria Goranskaya, Andrey Alexeyenko

**Affiliations:** Master program in Bioinformatics at Department of Biochemistry and Biophysics, Science for Life Laboratory, Stockholm University, Box 1031, 171 21 Solna, Sweden; Institute of Environmental Medicine, Karolinska Institutet, Box 210, SE 171 77 Stockholm, Sweden; Department of Biochemistry and Biophysics, Science for Life Laboratory, Stockholm University, Box 1031, 171 21 Solna, Sweden; Max Planck Institute for Human Cognitive and Brain Sciences, Stephanstraße 1a, 04103 Leipzig, Germany; Department of Microbiology, Tumour and Cell biology, Bioinformatics Infrastructure for Life Sciences, Science for Life Laboratory, Karolinska Institutet, 17177 Stockholm, Sweden

**Keywords:** Driver mutations, Passenger mutations, Somatic mutations, Copy number alterations, Gene networks, Network analysis, Cancer, Glioblastoma, Ovarian carcinoma, Brain cell compaction, Collagen cross-linking

## Abstract

**Background:**

In somatic cancer genomes, delineating genuine driver mutations against a background of multiple passenger events is a challenging task. The difficulty of determining function from sequence data and the low frequency of mutations are increasingly hindering the search for novel, less common cancer drivers. The accumulation of extensive amounts of data on somatic point and copy number alterations necessitates the development of systematic methods for driver mutation analysis.

**Results:**

We introduce a framework for detecting driver mutations via functional network analysis, which is applied to individual genomes and does not require pooling multiple samples. It probabilistically evaluates 1) functional network links between different mutations in the same genome and 2) links between individual mutations and known cancer pathways. In addition, it can employ correlations of mutation patterns in pairs of genes. The method was used to analyze genomic alterations in two TCGA datasets, one for *glioblastoma multiforme* and another for ovarian carcinoma, which were generated using different approaches to mutation profiling. The proportions of drivers among the reported *de novo* point mutations in these cancers were estimated to be 57.8% and 16.8%, respectively. The both sets also included extended chromosomal regions with synchronous duplications or losses of multiple genes. We identified putative copy number driver events within many such segments. Finally, we summarized seemingly disparate mutations and discovered a functional network of collagen modifications in the glioblastoma. In order to select the most efficient network for use with this method, we used a novel, ROC curve-based procedure for benchmarking different network versions by their ability to recover pathway membership.

**Conclusions:**

The results of our network-based procedure were in good agreement with published gold standard sets of cancer genes and were shown to complement and expand frequency-based driver analyses. On the other hand, three sequence-based methods applied to the same data yielded poor agreement with each other and with our results. We review the difference in driver proportions discovered by different sequencing approaches and discuss the functional roles of novel driver mutations. The software used in this work and the global network of functional couplings are publicly available at http://research.scilifelab.se/andrej_alexeyenko/downloads.html.

**Electronic supplementary material:**

The online version of this article (doi:10.1186/1471-2105-15-308) contains supplementary material, which is available to authorized users.

## Background

Cancer diseases result from stable perturbations in the network of functional interactions between genes and proteins. *Which* particular molecular mechanism was involved in any given case is less important than *where* in the network the alteration occurred. This is why attempts to understand cancer from the perspective of single genes or specific molecular mechanisms fail so often.

Recent large-scale investigations have demonstrated that cancer genomes are typically altered at multiple points in a single tumor [1–5]. Cancers with similar phenotypes may have hundreds of genomic alterations each, but these lists have low overlap with each other. There are two main reasons for this: 1) multiple different perturbations can generate identical cell states via alternative network routes [6], and 2) given the failure of chromosome repair mechanisms, spurious non-deleterious mutations start to occur at random. Many mutations discovered in cancer cells are thus neutral *passengers* that merely accompany functionally important *drivers* that have been subject to selective pressure. These mixtures of passenger and driver mutations together comprise the mutated gene sets (MGS) of the tumors in question. It is important to delineate the driving components of MGS both to facilitate basic research and to enable the development of individualized cancer therapies. Such information is important for several reasons. For example, some drivers should be targeted simultaneously during chemotherapy while others need to be targeted in a staggered fashion [7–10]. Similarly, it will be important to distinguish between different molecular subtypes of a particular cancer in order to identify the most appropriate treatment [11].

However, because each individual tumor will exhibit a unique combination of perturbations and random non-deleterious mutations, it is not trivial to identify the drivers within a given MGS. Estimates of the true driver fraction have ranged from a few percent [12] to around a half of all point mutations. The analysis of large, chromosome-scale copy number changes is even more challenging than that of point mutations. Specific chromosomal regions exhibit recurrent aberrations in over 50% of all cases of certain cancer types, such as ovarian carcinoma [13]. While these may include tens, hundreds, or even thousands of genes, it is intuitively clear that only a few of them are relevant to the disease. Many studies have been based on the hypothesis that mutations that occur frequently in cancers are most likely to contribute to cancer progression [1]. Sequence-based analyses have been used to distinguish between functional hot spots of individual genes such as *TP53*, which were identified as drivers, and non-functional mutations in the same gene [14]. Other authors have examined mutation patterns at the sequence level such as the ratio of synonymous to non-synonymous mutations [2] or the rates of break-of-translation mutations [15] while others have examined the potential functional consequences of specific mutation patterns [16] and the associated changes at the amino acid level [17]. Leary and co-authors [18] analyzed rates of sequence mutation and copy number changes simultaneously. To facilitate the discovery and classification of novel oncogenes and tumor suppressors, Vogelstein and co-authors [19] introduced the “20/20 rule”: a driver gene can be classified as an oncogene if at least 20% of its recorded mutations are missense mutations that occur at recurrent positions, and as a tumor suppressor gene if at least 20% of its recorded mutations are inactivating.

Many attempts to disentangle gain and loss patterns in large chromosomal regions have incorporated analyses of expression [20]. The GISTIC method identified driver gene copy number alterations (CNA) [21] by analyzing the statistical frequencies of various features, thus necessitating the use of large samples. Ciriello et al. [22] demonstrated that a given gene may exhibit different forms of alteration (e.g. copy number changes, point mutations, or changes in methylation) in different tumors. However, their method also can only identify cancer drivers in frequently mutated genes and chromosome-level patterns. As an example estimation of sample size required for such studies, the International Cancer Genome Consortium [23] determined that 500 samples per tumor type would be needed to detect a novel cancer gene that is mutated in at least 3% of patients. Vogelstein et al. [19] reviewed the challenges associated with the complex mutation landscapes of tumor genomes. Based on an analysis of 294,881 reported mutations from 3284 tumors that yielded only 125 discovered or confirmed drivers, they concluded that “at best, methods based on mutation frequency can only prioritize genes for further analysis but cannot unambiguously identify driver genes that are mutated at relatively low frequencies”. In other words, the vast majority of cancer genes have rates of mutation that are too low to enable their detection by frequency-based analyses. It will therefore be necessary to consider their functional and genomic contexts in order to determine their roles in specific cancers.

Considering the functional relationships between genes introduces a new dimension in the search and may radically improve the detection of driver mutations. One way to analyze these relationships is to establish and use a global network of functional couplings. Broadly defined, such a network consists of nodes (which represent genes, proteins, and potentially other molecules) and edges, i.e. functional links that connect them. An account of early efforts in the network analysis of disease genes and specifically those associated with cancer has been written by Ideker and Sharan [24]. The network edges are expected to link genes that were mutated in the same genome more densely than would be expected by chance alone. We have used this approach to validate a predicted human interactome [25] by examining mutations from *glioblastoma multiforme* (GBM) brain tumors obtained via The Cancer Genome Atlas project [26].

Torkamani and Schork [27] analyzed the functional contexts of mutated genes in co-expression modules. Cerami et al. [28] employed a network of protein-protein interactions to systematically evaluate the relationships between the most common mutations in GBM. They pooled the GBM point mutations in a larger sub-network (>300 genes) of potential drivers and investigated its modular structure. Subsequently, Ciriello et al. [29] utilized negative correlations (s.c. mutual exclusivity) between the most frequent mutations to identify multiple smaller modules, called cliques, in the protein interaction network. However, none of these methods were capable of detecting the involvement of specific mutations in each individual tumor. Gu et al. [30] also demonstrated that driver genes produced modular structures, and that different modules depended on each other in the network of physical protein-protein interactions. Babaei et al. [31] developed a multi-scale graph diffusion algorithm which confirmed that somatic mutations tend to gather around particular spots in the network. The dimensionality of such spots can range from single genes (frequent mutators) to larger pathway-like structures that are sparsely populated with rare mutations. An appealing feature of the three latter methods is that individual mutation effects are generalized to larger network modules which recur in different cancers. This reflects the observation that multiple driver mutations co-operate within a genome. In order to analyze large-scale chromosomal alterations, Akavia et al. [20] introduced an approach whereby copy number gains and losses were evaluated in terms of their impact on the expression of other genes and the associated modules, although this method also uses the frequency of occurrence as an input variable. In addition, functional relationships between genes were identified based on transcriptome responses within the analyzed dataset rather than being obtained from a large and previously-established interactome. Another advanced method for finding driver copy number alterations (CNA) [31] uses networks to identify the most important driver pathways rather than individual driver CNA events in particular genomes. This approach was largely based on the assumption that CNA changes the expression of either the directly affected gene or its network neighbors. On the other hand, many researchers (including Akavia and co-authors [20]) argued that, due to selective pressure, the expression of CNA drivers tends to be less correlated with their own copy number than the expression of certain passenger CNAs.

The common feature of all these frequency- and network-based methods is that they are *global*, i.e. require summarizing observations across multiple samples. However, it is intuitively clear that local contexts are important, and that the role of a certain mutation might depend on other alterations in the genome. As such, even rare mutations may be essential in driving a specific instance of cancer. However, it would be impossible to identify or study such rare mutations in a global analysis because of the low statistical power of the latter in such situations.

Therefore, we have developed a new, *local* approach to network analysis in order to distinguish between driver and passenger genes. We designed and have previously used an algorithm of the network enrichment analysis (NEA, [32]) to identify and probabilistically evaluate functional relationships between various experimental and known gene sets including GO terms [33], pathways [34, 35], differentially expressed transcript lists [32], and lists of candidate disease genes [36–38]. The key property of NEA is that it can be used to evaluate the statistical significance of observations by calculating the likelihood that they would occur by chance alone, i.e. in a random network. In our view, an optimal network-based algorithm would test individual mutation events against functional gene sets (FGS). Thus, NEA can identify driver mutations by considering the relationships between individual events in each somatic genome and 1) other mutations in the same genome and 2) genes that constitute known cancer pathways.

The article is organized as follows. We present:

1. The principle and main components of a new method for the network analysis of cancer genes and explain the choice of required components and parameters.
2. The results of an analysis of the two cancer sets that were published by the Cancer Genome Atlas consortium: glioblastoma (GBM) and ovarian carcinoma (OV) [13, 26]. We report all of the findings obtained and suggest potential biological roles for the most interesting novel drivers.
3. Results that validate our network-based method and comparisons of its performance to that of existing methods.

Finally, we discuss the strengths, weaknesses, and application domains of different approaches for identifying driver genes. The optimal global network and the software used in our analysis are made freely available. Our perl program NEA.pl can be used to perform multi-pronged and multilateral statistical evaluations of biological hypotheses in the network context.

## Results

### The method: parallel procedures to test driver roles

The application of NEA [32] to single mutations can be briefly described as follows: it evaluates significance of the functional relation between the mutated gene and a pre-defined set of genes which are known or supposed to functionally relate to each other. This is done by counting network edges (links) between the given gene and any other genes of the set in the actual global network. Next, the links are counted in the same way in a *random* network, which provides the count expected by chance. If it is significantly lower than the actual count (given the observed level of variance in a sufficiently big series of random networks), then NEA claims that there is functional relation between the mutation and the gene set. The details are explained in the section “Connectivity tests” (see Methods).

**Figure 1:**
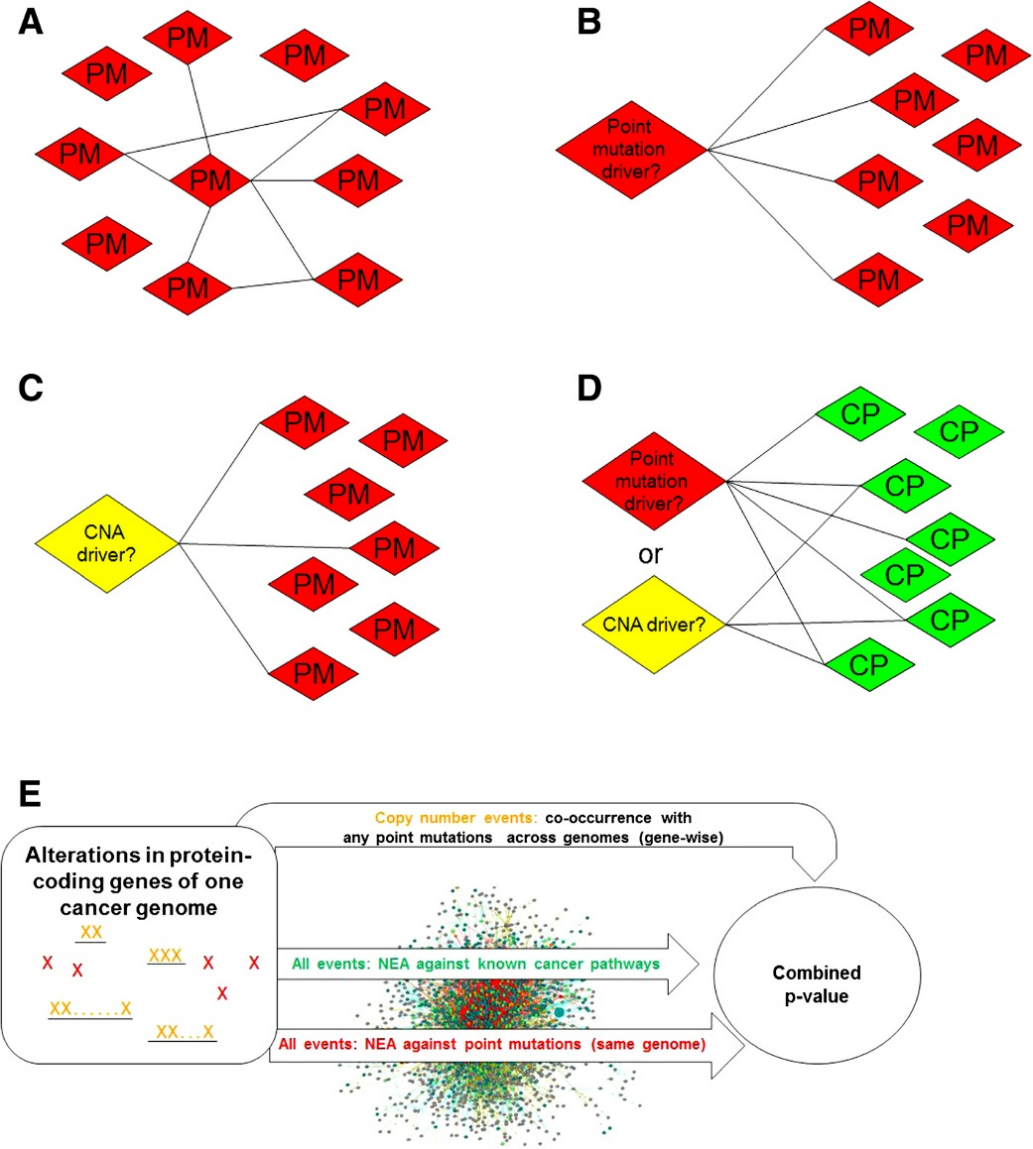
**Schematic representation of the network enrichment analysis applied to detection of driver mutations. A**, total quantification of inter-relations between somatic point mutations (PM) in one genome. **B**, test of a single point mutation for being related to all other PMs (1point-vs-MGS). **C**, test of a copy number alteration (CNA) against all PMs in the same genome (1CNA-vs-MGS). **D**, test of either a CNA or PM against a known cancer pathway (CP), irrespective of genome (1-vs-CPW). **E**, overview of the algorithm. The analyses at B, C, and D were summarized into a single combined p-value for each gene copy number change (yellow) and point mutation (red).

The multiplicity of observed somatic mutations in most cancer genomes indicates that the emergence of cancer might require perturbations at multiple network points. This conjecture was confirmed in our previous work [25]: many individual, tumor-specific sets of somatic mutations in GBM exhibited coherence in the global network context when analyzed as whole groups (or mutated gene sets, MGS). A representative case is shown in Figure 1A. This coherence was demonstrated by the presence of a greater number of connections between simultaneously mutated genes than the number expected by chance alone (analysis details for the GBM and OV sets are given under the heading “Coherence of genome-specific sets of point mutations” in the Methods section). This allows us to suggest that MGSs could be used as functional gene sets needed for the NEA tests in the current work. Each particular mutation in the MGSs may be either a passenger, and then no enrichment to the rest of MGS should be detected, or a driver, and then we should obtain a significant network enrichment score (if the global network contained relevant edges). In addition to using MGSs, we can test each mutation against known cancer pathways. In this case, we expect that the mutation interacts with pathway genes, while the latter are not necessarily mutated in this genome. Thus, we applied three modes of NEA in parallel, independently of each other (illustrated in panels B, C, and D of Figure 1), and combined their results at the last step (Figure 1E). In these modes the individual genomic alterations (i.e. point mutations or copy number changes that could influence protein-coding genes) were evaluated against:

1. the sets of all point mutations in the same genomes (referred to as 1point-vs-MGS or 1CNA-vs-MGS evaluations, respectively), or
2. the known cancer pathways (1-vs-CPW);It should be noted that we did not analyze copy number changes with respect to one-another because they were very abundant and positionally coupled, so that such an analysis would have yielded high false positive rates. Instead, we evaluated each of the copy number altered genes for their statistical significance of co-occurrence (CO) with any point mutations across tumors of the same cancer type (Figure 1E; details of this analysis are provided under the heading “Validation by co-occurrence of mutations” in the Results section). Thus, after completing all these tests, each mutation event was assigned two or three (if the CO analysis was included) separate p-values, which were then combined using Fisher’s formula and adjusted for multiple testing. According to the Fisher’s definition, a significantly low combined p-value would suggest that the candidate driver was involved in at least one (and possibly two or all three) of the roles. The candidate drivers were then ranked according to their combined p-values.

### Selection of parameters

#### Network enrichment statistics

Our software was capable of calculating network statistics (see “Connectivity tests” in Methods) by counting both direct links (i.e. existing network edges) and indirect links in which two genes shared a neighbor (i.e. both nodes of interest had edges connecting them to the same third node). A preliminary study indicated that the inclusion of indirect links improved the method’s performance when dealing with sparse networks. While the latter are discussed in the following section, the method’s overall sensitivity and specificity when analyzing sparse networks were consistently worse than those achieved for more dense networks even if only direct links were considered in the dense cases (data not shown). We therefore primarily employed analyses using direct links.

#### Choosing the optimal network

So far, many alternative versions of the global network of functional coupling in human (otherwise called gene regulatory network, interactome etc.) have been made public. Hence generation or compilation of a novel network version was beyond our focus in this work. In order to optimize the discovery of cancer drivers, we wanted to identify by benchmarking the most efficient global network in our collection of public and custom networks, also considering possible merges thereof. The previously published network analyses have often utilized physical protein interactions obtained from the literature and high-throughput experiments [28, 29, 38]. However, given the multiplicity of interaction mechanisms in the underlying biological network, the systematic *integration* of diverse high-throughput data types should provide a more informative resource [39–42]. Our FunCoup framework [25] enables the construction of such integrated networks of high confidence and coverage, which was achieved by incorporating eight different types of data from a range of model eukaryotes as well as from the human itself. However, the relevance of such broad data collections in the cancer domain was questioned. *A priori*, it was not clear whether it would be best to focus on a network with the maximum achievable size and coverage (with the risk of lower specificity as a possible trade-off), a high-confidence curated network, or a cancer-specific network based on only the most immediately relevant data.

**Figure 2:**
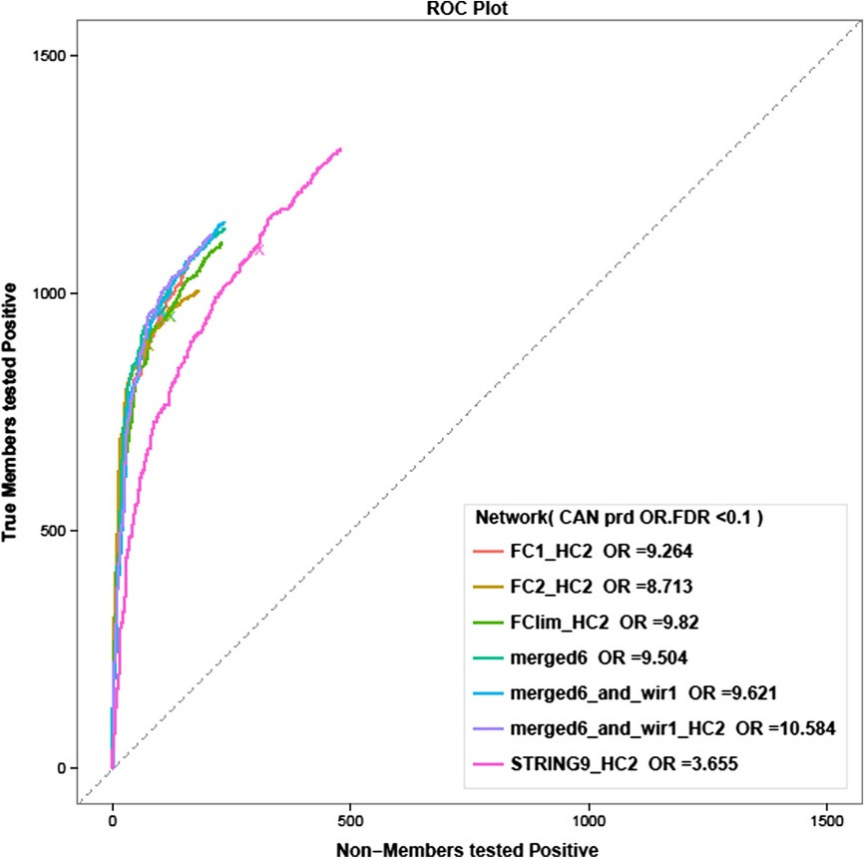
**ROC curves allow evaluating differential performance of global network versions in predicting members of cancer-related gene sets.** Crosses: points where FDR = 0.1. OR (“odds ratios”) quantify sensitivity/specificity trade-offs as ratios of Y/X coordinates at the cross points.

To clarify this issue, a benchmarking study was conducted (for details, see the Methods section). The best performance was achieved with a network obtained by merging the FunCoup data with links available from a number of curated databases (Figure 2). All of the analyses presented herein were based on this combined network, which was named merged6_and_wir1_HC2 and is available for downloading at http://research.scilifelab.se/andrej_alexeyenko/downloads.html. For the sake of simplicity, the types, origins, and strengths of the network edges are omitted in figures highlighting relevant examples.

### Discovery of driver mutations

#### Point mutations

We tested all of the point mutations reported in the MAF files for the GBM and OV sample collections, for which 1020 and 14842 somatic point mutation events in gene coding regions were identified, respectively. Using the procedure described above (the first section of Results), we performed 1point-to-MGS and 1-vs-CPW network analyses for each mutation. By imposing a minimal adjusted combined p-value confidence threshold of 0.01, we classified 591 and 2506 mutations in the GBM and OV sets as drivers (Sheet 1 in Additional file 1), where these numbers corresponded to 259 and 1258 distinct genes, respectively. The two sets were originally produced using different approaches: the OV set was generated in a whole-exome effort whereas the GBM set was generated by analyzing a pre-defined set of around 600 genes selected on the basis of previous cancer studies. The MGS for GBM were consequently smaller (7 mutations per GBM sample compared to more than 50 per OV sample) and were found to contain a greater proportion of drivers in our analysis: 57.8% of the GBM mutations were identified as drivers compared to only 16.8% of those in the OV set. The sensitivity of the analysis of the GBM set thus appeared to represent a trade-off between lower MGS sizes (which would be a disadvantage for NEA) and a greater proportion of real drivers due to the target gene pre-selection (which increased the statistical power of the analysis). The 1-vs-CPW procedure was, on average, more sensitive than the 1point-vs-MGS one: around 50% of all detected drivers could have been successfully qualified using 1-vs-CPW alone. However, the 1point-vs-MGS analysis complemented the 1-vs-CPW results by increasing the overall confidence and sensitivity.

How often were mutations in the same gene classified differently in different samples (i.e. as drivers in one case and passengers in another)? There were 25 such genes out of 196 that were mutated twice or more in the GBM set, and 94 genes out of 2755 multiply-mutated genes in the OV set. Most of these mutations occurred in genomes with small MGS size, which were not amenable to 1point-vs-MGS analysis. However, many of them had also been linked to known cancer pathways in previous studies and were therefore assigned low p-values in the 1-vs-CPW analysis.

**Figure 3:**
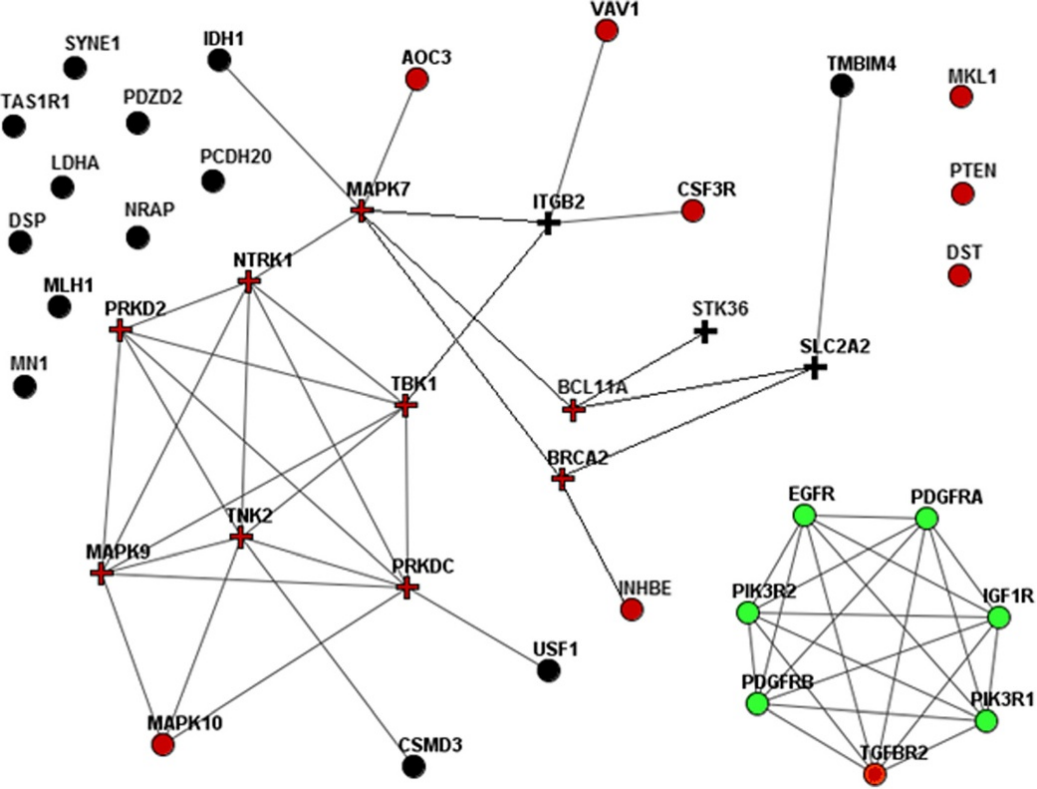
**Network enrichment analysis of point mutations in genome TCGA-02-0014.** The likelihood that one of the 34 mutated genes in the genome (indicated by red and black nodes) was determined either based on their linkage to the set of other 33 point mutations (black crosses), or in the context of known cancer pathways (red circles), or by both criteria (red crosses). Mutations that did not reveal any functional involvement are indicated by black circles. Linkage to cancer pathways is exemplified by the relationship between TGFBR2 and the genes of the KEGG05214 glioma pathway (green circles). Other relationships of this type are not shown.

Figure 3 exemplifies the network analysis with 34 point mutations from the GBM sample TCGA-02-0014. More than half of the genes were assigned low combined p-values because they had significant connections to known cancer pathways (red circles), to other genes with point mutations in the same genome (black crosses), or both (red crosses). Nine genes were qualified as passengers in this set (black circles). The network view reiterates the importance of statistical estimation: there were several cases in which a node had many links but was not significant. For example, while MAPK10 had three edges connecting it to other mutated genes, it was not qualified as significantly linked to this MGS because the MAP10K node itself had a high degree (468) as did the three other mutated kinases (MAPK9, TNK2, and PRKDC, which had 403, 360, and 1212 network edges, respectively). The presence of three edges was therefore considered to be spurious in this case. However, it should be noted that MAPK10 was identified as a driver in the CPW analysis.

#### Resolving copy number altered regions: gene copy gains and losses versus somatic point mutations

This section describes the justification for the CNA analysis, the procedure for performing it, and the results obtained using it.

Both the GBM and OV genomes often exhibited substantial chromosomal re-arrangements that involved thousands of potential driver genes. Therefore, using CNAs in the same way as point mutations would have resulted in a low power to detect functional involvement. Another complication was that even though many chromosomal segments appeared to be recurrently affected across either GBM or OV, their borders were ambiguous (i.e. varied from genome to genome). This again justified the use of the gene-wise approach.

First, we checked whether copy number changes had a tendency to be related to the point MGS in the same cancer genome. CNA genes were indeed enriched in functional associations with sets of point mutations. For example, at a threshold of 35.3% below the normal copy number value, significant enrichment was demonstrated for both the GBM and OV sets (the p-values of the Fisher’s exact test were below 10^-13^ in both cases).

As with the pairwise co-occurrence of point mutations described above (in the section on “Validation by co-occurrence of mutations”), we observed many cases in which CNAs co-occurred with point mutations in other genes. However due to the large sizes of the studied chromosomal fragments, chromosomal neighbors appeared in large clusters with identical or very similar patterns of association with certain point mutations. Such extended chromosomal fragments have long been linked to cancer in epidemiological studies, but identifying specific drivers among their many genes remained challenging.

In order to identify CNA drivers, we applied our network analysis to every CNA gene within each of the chromosome fragments. For each such gene, we calculated

1. their co-occurrence with each point mutation in the cohort; only genes having a p-value of <0.01 for co-occurrence with at least one point mutation according to Fisher’s exact test were regarded as potential drivers,
2. 1CNA-vs-MGS, i.e. their NEA z-scores were calculated with regard to MGS for all genomes in which the copy number of the corresponding gene was changed, and
3. 1-vs-CPW, i.e. the NEA z-scores for their relationships to known cancer pathways.

The p-values for these tests were combined hierarchically as described in the *Methods* and used to prioritize potential CNA drivers (Sheet 2 in Additional file 1). This analysis was much more sensitive for the GBM data set than for the OV set, yielding 365 and 90 prioritized driver CNAs with combined p-values of *p* < 10^-6^ and 232 and 61 genes with combined p-values of *p* < 10^-12^, respectively. Aside from possible biological differences between the sets, the main reason for this is probably the lower sensitivity of NEA against point MGS in the OV set (which contained a much greater proportion of passengers than the GBM set, as discussed above). We note that the whole analysis (i.e. that based on the application of all three conditions together) was probably too conservative and may have yielded many false negatives.

**Figure 4:**
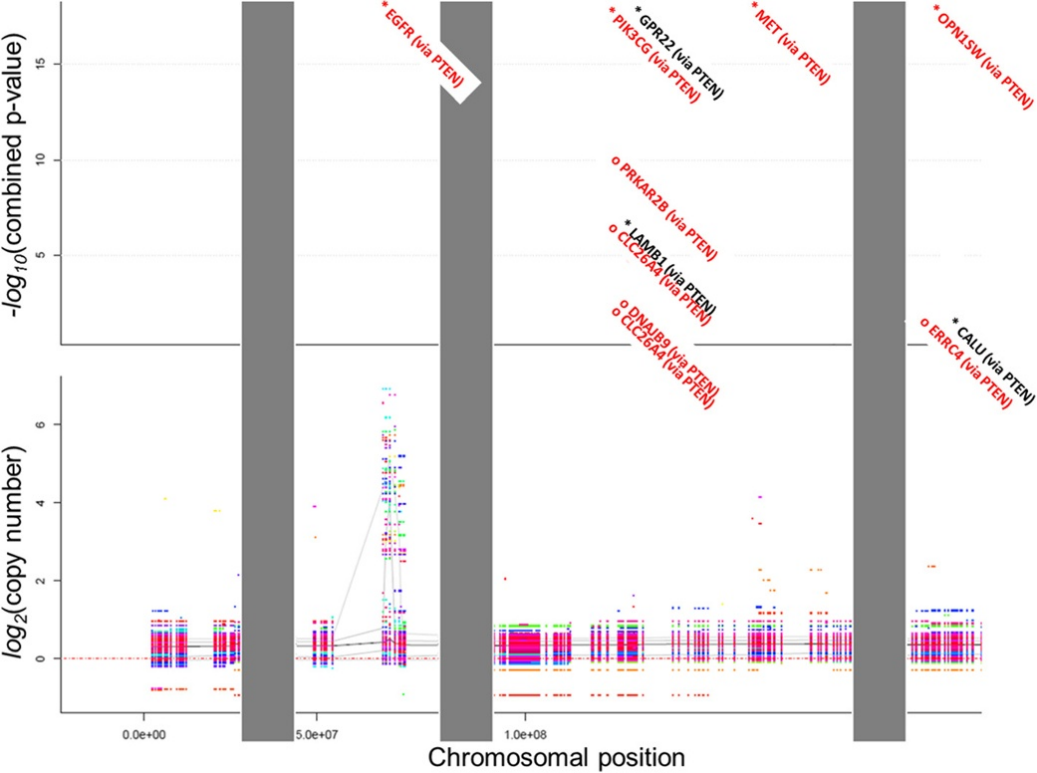
**Driver analysis over extended chromosomal regions.** All the components of NEA were run over copy-number altered regions of chromosome 7 in the ovarian cancer. The grey areas mask the chromosomal regions omitted from this plot, so that only four selected regions are shown. Multi-colored bars in the lower plot indicate the copy numbers in individual OV genomes relative to the reference diploid genome (dotted red line). Grey lines indicate the 10^th^, 25^th^, 50^th^, 75^th^, and 90^th^ percentiles of copy number in the OV cohort. The upper plot shows the prioritization of genes based on the–log_10_ of their combined p-values: genes with higher positions are more highly prioritized. Genes found to be highly significant in the 1-vs-CPW analysis are highlighted in red, those highly significant in the 1CNA-vs-MGS analysis are marked with a black asterisk, and red asterisks indicate genes highly significant in both analyses. Gene symbols in brackets indicate point mutations that co-occurred with the driver CNAs. A complete version of this figure can be found in the Additional file 2 and Additional file 3: GBM.CNA_drivers_along_chromosomes.pdf and OV.CNA_drivers_along_chromosomes.pdf.

We visualized the results of all three tests and their combined results using chromosomal maps (Figure 4 and Additional file 2: GBM.CNA_drivers_along_chromosomes.pdf and Additional file 3: OV.CNA_drivers_along_chromosomes.pdf). Figure 4 shows the results of the analysis for chromosome 7 in the OV set. While the copy numbers varied along the chromosome’s length, there were only a few regions in which these variations significantly co-occurred with point mutations in such genes as TP53, BRCA2, or TTN (see names in brackets) and thus satisfied the first condition. Next, only some of these genes were further functionally linked to either a given MGS (indicated with an asterisk) or to a particular cancer pathway (indicated by red coloration). A few genes satisfied all three criteria: EGFR, PIK3CG, HBP1, OPN1SW, MET, and CALD1. The left chromosomal arm probably exhibited a tendency toward duplication primarily because this increased the copy number of EGFR. Variations in the other chromosomes may have affected a number of different drivers. Interestingly, in the GBM cohort, EGFR CNAs co-occurred with point mutations in the same gene (mostly of the missense type): out of 24 genomes with point mutations in EGFR, 22 also contained EGFR duplications. However, there were 72 other GBM samples that contained EGFR duplications but no EGFR point mutations.

**Figure 5:**
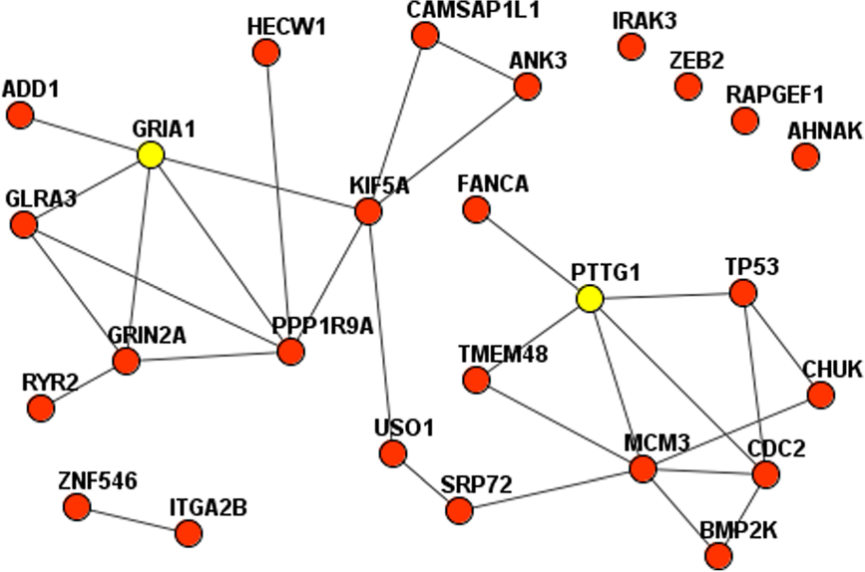
**Close functional relationships between genes with point mutations and copy number changes in the same somatic genome.** The relations were first prioritized for their significant co-occurrence with point mutations in TP53, and then for enrichment in network connections with other point mutations. Red nodes indicate genes with point mutations detected in the OV tumor sample TCGA-13-0906-10; Yellow nodes indicate a pair of genes that both exhibited copy number losses in the same genome, both of which were highly ranked as drivers in this genomic context.

Analyses spanning multiple samples revealed interesting functional relationships. For example, copy losses in a region of chromosome 5 strongly suggested that both GRIA1 and PTTG1 were potential drivers. Both of these CNAs were originally prioritized because of their co-occurrence with point mutations in TP53, and subsequently exhibited tight functional links to specific MGS. In a sub-network of two MGS involving GRIA1, PTTG1, TP53, and other MGS members from two representative OV genomes, only PTTG1 was directly linked with TP53 (Figure 5). The functional association between GRIA1 and TP53 was thus revealed via their connections to other mutations.

#### The collagen network

**Figure 6:**
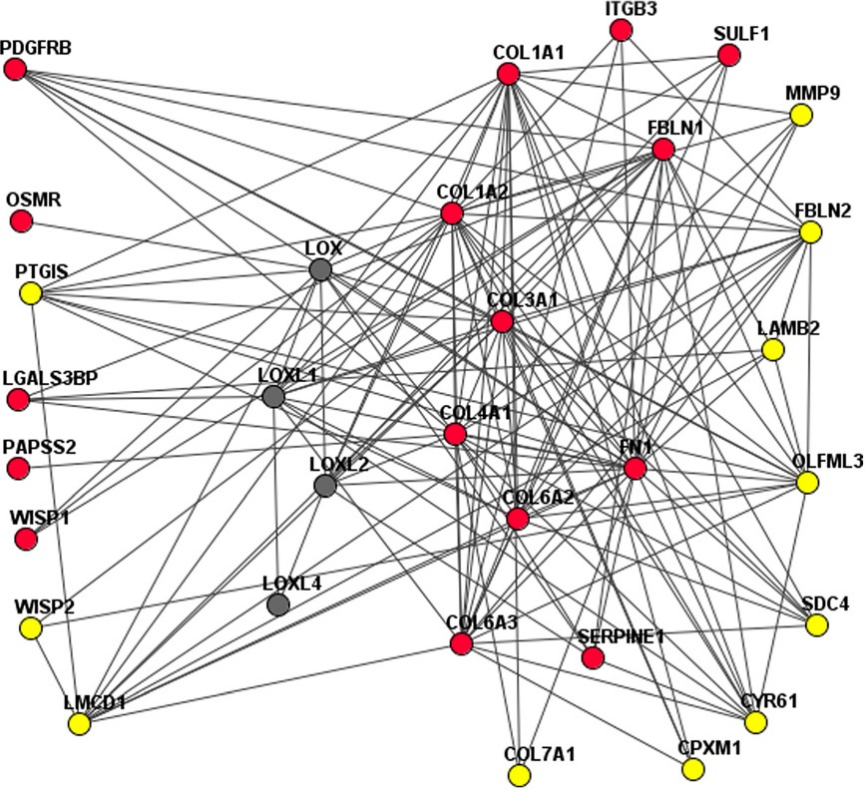
**Collagen-associated network affected by somatic mutagenesis in GBM, as identified by the network analysis.** The sub-network was retrieved from http://funcoup2.sbc.su.se by searching for direct links that connect genes with point mutations (red), copy number alterations (yellow), and LOX genes (grey). Each of the red and yellow nodes represents a gene with a driver role established via 1) network enrichment analysis against either known cancer pathways or sets of genes with point mutations in the same tumor, and 2) significant association (co-occurrence) with collagen mutations (or being collagens themselves). The network edges shown in the figure are independent of the latter analysis because the whole network was compiled using the FunCoup tool based on multiple sources of high-throughput, annotation, and literature data.

In addition to the oncogenes and tumor suppressors that perform controlling functions in cancer, we identified a group of structural proteins with apparent functional roles in GBM and OV. Mammoto et al. [43] summarized the molecular mechanisms of brain cell compaction and angiogenesis in *glioblastoma multiforme*. In experimental studies on glioblastoma cell lines, these authors demonstrated that changes in the expression of genes encoding proteins such as lysyl oxidase (LOX), collagens of groups 2, 4, and 6, and metalloproteinases 2 and 9 were associated with changes in the physical microenvironment of the extracellular matrix in neoplastic brain tissues. Collagens were affected by point mutations in 16 of the 148 GBM genomes and 102 of the 326 OV genomes. All of these cases from the GBM set and around 50% of those from the OV set scored highly in our driver analysis. Furthermore, many collagens exhibited either copy number alterations or had point mutations that co-occurred with CNA in other genes. However, neither LOX itself (which enables neoplasia by cross-linking collagen chains) nor the three human LOX homologs exhibited any genomic alterations. Figure 6 shows a sub-network that combines all of the relevant proteins that exhibited mutagenesis in the GBM set. Each of the presented genes is connected to multiple structural and regulatory interactors of the extracellular matrix, tumor-related angiogenesis, and tissue formation: matrix metalloproteinase MMP9, fibronectin FN1, fibulin-1 FBLN1, laminin beta LAMB2, extracellular sulfatase SULF1, oncostatin M OSMR, bifunctional 3’-phosphoadenosine 5’-phosphosulfate synthetase-2 PAPSS2, galectin-3-binding protein LGALS3BP, “LIM and cysteine-rich domains 1” LMCD1, PDGF receptor PDGFRB, prostacyclin synthase PTGIS, probable carboxypeptidase X1 CPXM1, syndecan SDC4, WNT1-inducible proteins WISP1and WISP2, and cysteine-rich angiogenic inducer 61 CYR61.

Each of the nodes shown in Figure 6 (aside from the LOX genes) represents a gene with either a point mutation (red) or a CNA (yellow) in GBM. Their roles as drivers were confirmed by both NEA and their co-occurrence with other mutations (Additional file 1). On a larger scale (data not shown), this sub-network was linked to EGFR through FN1, LMCD1, PDGFRB, tenascin TNC, and integrin beta-3 ITGB3, as well as to protein kinases PIK3CA and PIK3C2A through CPXM1, SDC4, and WISP2.

A similar pattern was observed in the OV samples, although in these cases somatic mutagenesis affected collagens of classes 11, 12, 14, and 16, as well as genes encoding HSPG2 (basement membrane-specific heparan sulfate proteoglycan core protein) and MXRA5 (matrix-remodelling-associated protein 5).

#### Novel drivers

Many of the driver mutations identified in this study have not previously been linked to glioblastoma and/or ovarian carcinoma. As far as we could see, around 30 of the GBM drivers identified in this work were neither known members of cancer pathways nor mutated in cancers of the central nervous system other than those from the TCGA dataset. More specifically, a number of the identified protein kinases had not previously been known for their involvement in *glioblastoma multiforme*, although the literature does contain some indirect evidence suggesting that they may be tumor suppressors or oncogenes.

On the other hand, some of the identified drivers were previously known to be involved in cancer; these are discussed here.

The possibility that the protein kinase ATR may play a role in cancer was first revealed quite recently, by Toledo et al. [44]; these authors found that when activated by replication, this protein locally protects replication forks and globally suppresses origin firing. Cerami et al. [28] identified a functional module of 4 genes (DCTN2, TUBGCP2, TUBGCP6, and FGFR1OP) that encode components of the centrosome and microtubule organizing center. By testing the data analyzed by these workers using our 1point-vs-MGS procedure, we found that another protein kinase ATM should be included in this module because it was a likely driver in two GBM and in two OV genomes. The kinase CSNK1E had been previously associated only with non-brain cancers [45, 46], while HIPK2, LYN, and EPHB4 have been suggested as targets for anti-tumor therapies [47–49]. PIK3C2B was considered to be a possible cause of resistance to erlotinib during the later stages of glioblastoma, i.e. it may become a driver in tumors that have evolved beyond a certain point [50].

In general, the top ranking driver point mutations and copy number changes were significantly more likely to affect genes encoding protein kinases (PK) and transcription factors (TF) than other types of genes. These two categories exhibited both gene set enrichment, i.e. they were over-represented among the set of identified drivers, and network enrichment, i.e. there was an over-representation of network edges connecting other drivers to PK and TF genes such as RUNX1, SIK1, ETS2, VSX1, FOXA2, SOX1, BMP2, TP53BP1 in the GBM set and TGFBR2, ACVR1, ACVR2A, WNT6, WNT16, STAT4, PRKRA, PDGFRA, PAX4, GLI3 in the OV set (Table 1).

**Table 1:** **Enrichment of genes encoding protein kinases, transcription factors, and genes linked to these categories in the global network**

	Driver mutations			GO term	No. of genes	No. of network edges, total	No. of shared genes	FDR(GSEA)	No. of network links	FDR(NEA)
Set	Type	No. of genes	No. of network edges, total	Title						
GBM	Point mutations	277	40863	Protein kinase activity	277	49534	50	1.10e-63	3680	0.00e + 00
	Point mutations	277	40863	Regulation of transcription, DNA dependent	453	84621	19	1.05e-05	2340	1.14e-40
	Copy number	455	49817	Protein kinase activity	277	49534	32	2.79e-18	3379	0.00e + 00
	Copy number	455	49817	Regulation of transcription, DNA dependent	453	84621	40	2.99e-16	2817	1.87e-44
OV	Point mutations	1611	168022	Protein kinase activity	277	49534	103	1.52e-50	10941	0.00e + 00
	Point mutations	1611	168022	Regulation of transcription, DNA-dependent	453	84621	93	8.99e-19	9766	0.00e + 00
	Copy number	168	24513	Protein kinase activity	277	49534	12	1.71e-07	1247	0.00e + 00
	Copy number	168	24513	Regulation of transcription, DNA-dependent	453	84621	7	0.29	1289	1.15e-11

FDR (GSEA): significance for fractions of PK and TF as shared genes.

FDR (NEA): significance for fractions of genes connected to PK and TF in the network.

### Comparison with existing methods and gene sets

It should be noted that each of the three major classes of methods possessed own features, which did not allow a symmetric, uniform comparison. Namely, the methods that used sequence and frequency were both less dependent on existing knowledge, although not entirely free of it. For comparison, the network methods could explicitly employ literature data in the form of known pathways, edges of the global network etc. Next, both the sequence and network based methods were applicable to events of any frequency, including unique ones. And lastly, both the frequency and network based methods could use plane genes symbols, whereas the sequence analysis required specifying nucleotide changes exactly. These circumstances warranted applying specific approaches to the comparisons presented below.

#### Sequence analysis methods and mutation frequency approaches

Sequence-based methods tend to be specialized for the analysis of specific mutation types such as copy number changes, gene fusions, or short insertions/deletions/substitutions. They evaluate concrete alterations of the nucleotide sequence in each gene and assess their potential impact on the protein’s function. If the predicted impact is strong, the mutation is deemed damaging.

In order to compare NEA with sequence-based methods, we uniformly submitted the point mutations from the GBM and OV sets to three web services that can estimate the impact of point mutations on peptide chain functionality: Mutation Assessor [51], PolyPhen-2 [52, 53], and SIFT [54]. Of course, the classifications obtained using these services are not identical to those used when defining driver mutations in cancer biology, but they are what cancer scientists look for when using these resources. The methods’ efficiency was probably limited by a lack of information on homologous sequences and protein structures in their databases, although the extent to which they rely on such information varies.

The overall positive rate of the sequence-based methods was very high. For example, around 40% of 4896 mutations in the OV genomes were predicted to be damaging by at least one of the three sequence-based tools (while as few as 3% were identified as potential drivers by 1point-vs-MGS NEA at FDR <0.1). On the other hand, one would expect to see functionally significant peptide chain alterations in most frequently mutated genes. We did note that 70-80% of EGFR (in OV) and TP53 (in both OV and GBM) mutations were predicted to be either high impact or damaging. However, this was not the case for most of the other genes that were mutated in more than five (and up to 70) genomes each (NF1, IDH1 etc.). Between 35 and 40% of mutations in these genes were classified as having “damaging” or “high” impact, which is no greater than the rate for genes in which mutations occurred only once (Additional file 4: Figure S7, discussed in the next section). Furthermore, these high positive rates did not decrease in the OV set, where mutations in ~13000 genes were analyzed. For example, the group of 3899 genes mutated in a single OV sample each also had a positive rate close to 40%. This was a counterintuitive result, especially when compared to the GBM set with its ~500 mutated genes pre-selected by known implication in cancer. Considering the sequence analysis results in the gold standard sets of frequently mutated and validated GBM drivers from Parsons et al. [5] and Vogelstein et al. [19], we also could not see much difference from the bulk of the genes. Finally, the three methods were in equally poor agreement with each other as with our method (Additional file 4: Figure S4). It could be noted that a smaller fraction of mutations were identified by all the four (including our NEA) methods (35 and 791 in GBM and OV, respectively), but these were mostly the well known cancer drivers.

The set of drivers predicted by 1-point-vs-MGS NEA at FDR < 0.1 only exhibited a formal overlap with the results obtained using two tools, GeneSift and PolyPhen-2 (and only in the GBM and not in the OV dataset). The 1-point-vs-MGS NEA agreed quite well with respect to frequency: frequent mutators were classified as drivers much more often than single-case mutators (5 - 32% compared to 3 - 5% based on the pooled GBM and OV results). As explained above, the sequence based tools did not show such agreement.

There were still remarkable exceptions. Many known drivers such as TP53, PTEN, RB1 etc. did not receive any significant 1point-vs-MGS scores in genomes with few point mutations. On the other hand, these genes were detected by 1-vs-CPW analysis. As another example, NEA missed nearly all of the mutations in IDH1, an enzyme with acknowledged involvement in GBM [55], because of its unique global role in DNA methylation, which was not reflected in our network.

Further, despite the poor correspondence between the sequence tools and NEA, the results obtained with the latter were in good agreement with the basic variant classification data available from the TCGA maf files. The strengths of the differences between silent mutations and those classified as either “missense” or “nonsense” increased with the confidence of the NEA analysis: mutations with 1-point-vs-MGS NEA z-scores of > 10 exhibited the most significant overlap with the “missense OR nonsense” category (p-values of p < 0.01 according to Fisher’s exact test for GBM and p < 0.00001 for OV; Additional file 4: Figure S5).

We conclude that sequence-based methods are likely to yield very high positive rates, which seems especially counter-intuitive when whole-exome mutations sets are analyzed (such as the OV set). Moreover, they are only consistently efficient for a few well-characterized genes such as EGFR and TP53. However, NEA also has some significant limitations, including its inapplicability to rare mutations in small mutation sets and genes with very special roles (e.g. those that extend beyond well-characterized signaling pathways).

#### Gold standard sets

We also compared the results of our NEA tests to selected gold standard driver lists, namely:

1. 42 GBM CNA-genes presented by Parsons et al. ([5], see Additional file 4: Table S7 to that article), and two lists created by Vogelstein et al. [19];
2. 43 cancer predisposition genes ([19], Additional file 4: Table S4);
3. 138 mut-driver genes ([19], Additional file 4: Tables S2A and S2B).

List (1) was compiled based on an integrated analysis of sequence changes, amplifications, and homozygous deletions, and had three different p-value levels for each gene (Passenger Probability Low, Passenger Probability Mid, and Passenger Probability High). We could therefore calculate the correlations between each of these three categories and the three NEA p-values obtained using the 1-vs-CPW, 1-point-vs-MGS, and 1CNA-vs-MGS procedures (Additional file 4: Figure S6A). Despite the very small gene sets used (11 to 33 genes were available for each comparison), the overlaps proved to be stable and positive.

The list (2) was compiled from the Cancer Gene Census and did not contain quantitative scores, so we could only calculate the enrichment of NEA-prioritized genes in this list (Additional file 4: Figure S6B). Again, there was significant and stable concordance despite the small number of overlapping genes in the two sets. The significance of enrichment increased with the stringency of the combined NEA p-value cut-off (in all of the 1-vs-CPW, 1point-vs-MGS, and 1CNA-vs-MGS analyses).

Finally, the strongest concordance was observed between the NEA p-values and the mut-driver list (3). The latter was compiled by Vogelstein and co-authors [19] according to their “20/20 rule”, which states that a gene can be classified as an oncogene if at least 20% of its recorded mutations are missense mutations that occur at recurrent positions, and as a tumor suppressor gene if at least 20% of its recorded mutations are inactivating. Of the 134 genes that were included in both sets, 101 had 1-vs-CPW p-values of <0.001 (FDR < 0.05). Of the 133 genes available for 1CNA-vs-MGS analysis, p-values of <0.001 were observed for 50 and 48 genes in the GBM and OV sets, respectively (the results were combined across MGSs). Finally, of the 52 and 39 genes from the mut-list that had somatic point mutations in the GBM and OV sets, 22 and 6, respectively, had 1point-vs-MGS p-values of <0.001 (also combined across MGSs). Enrichment by Fisher’s exact test in these analyses was significant at all cutoffs (data not shown).

When the positive predictive rates of the network analysis were plotted against relative mutation frequencies in the GBM and OV sets, the gold standards by Parsons et al. [5] and Vogelstein et al. [19] (which were both based on the *glioblastoma multiforme* analyses) demonstrated somewhat better results than the sets of all the mutations of the TCGA GBM and OV sets (Additional file 4: Figure S7). However, the difference was not found significant: frequent mutators of both the gold standard sets and in the bulk of TCGA genes demonstrated higher rates of positive NEA predictions.

We conclude that the validity of the NEA results was confirmed by their significant overlap with these three published gene collections. We also note that although the mut-drivers were discovered by analyzing point mutations, many of the genes in the GBM and OV sets exhibited driver copy number alterations as well.

#### Validation by co-occurrence of mutations

Given the assumption that driver perturbations in multiple key sub-pathways are required for cancer development, one might expect genes from different network domains to exhibit co-occurring mutations in cancer MGS. The presence of such non-random patterns would provide alternative evidence that a given gene is a driver. Conversely, passenger point mutations would not be expected to behave in this way. It should be noted that CNA pairs were not analyzed in this way because of their positional interdependence. We calculated the associations between pairs of somatic point mutations and between somatic point mutations and CNAs. Significant ones were found both for genes with frequent mutations (TP53, PTEN, DST, RB1, IDH1) and for genes in which these events were rare. Many of the latter category were affected by CNA (as shown in Table 2).

**Table 2:** **Representative 2x2 table of mutation co-occurrence across GBM genomic samples (Fisher’s exact test** ***p***_***0***_ **= 2.6** ^**-07**^ **)**

		Point mutation in FN1	
		Yes	No
Point mutation in MSH6	Yes	4	0
	No	1	143

To verify the consistency of these patterns using NEA, we compared the summed 1point-vs-MGS NEA scores across all samples in which a given gene had point mutations to the number of co-occurrences with any other mutation. The list of predicted drivers with high sums overlapped significantly with the list of genes with co-occurring mutations (Additional file 4: Figure S8; one-sided binomial test p-values of 0.00027 and 0.000008 were achieved for GBM and OV, respectively). Importantly, pairs of genes with correlated mutation patterns were usually not directly connected by network edges (we found only 35 such pairs in total). Nonetheless, our method was able to characterize these genes as drivers by utilizing higher-order interactions involving multiple genes as shown in Figure 5 and discussed at length above. This stands in contrast to the results obtained by Ciriello at al. [29], who based their mutual exclusivity modules on known links in a protein interaction network.

Thus, the co-occurrence analysis in pairs of driver mutations confirmed the overall validity of NEA. When applied to mutations from the same sets, the results obtained were practically independent of the network context. As described above, we utilized the association analysis as an auxiliary part of our method.

#### Comparison to MEMo algorithm

Most of the methods of network analysis could only discover multi-genic entities such as network modules, putative pathways, motifs, gene signatures etc. and thus were not directly comparable to our method. However, we could still use the results published by Ciriello at al. [29] by considering individual genes from their modules. Their MEMo algorithm was applied to the same data with a different approach: by pooling genomic samples they discovered groups of frequent mutations that were negatively correlated with each other and fully connected in the network of protein-protein interactions. Due to these strict requirements, the list of significant modules was quite short and most of the members (in total, 19 genes in GBM and OV in either of the two network versions by requiring FDR < 0.1) were assigned to multiple modules. We found that our analysis successfully assigned very low combined NEA p-values (below 10^-10^) to all the 13 GBM genes and to 5 out of 7 OV genes identified by MEMo. It should be noted, however, that there were crucial differences between MEMo and our approach. First of all, we were more flexible by looking at individual mutations and by defining network enrichment in a much looser manner. In addition, the detection of negative correlations needed for MEMo is generally much more challenging than that of positive ones (as described in the previous section) because most of the mutations have low marginal frequencies.

## Discussion and conclusion

The mutated gene sets for glioblastoma and ovarian tumors contained both driver and passenger mutations. Synergies between drivers in individual tumors were elucidated via their functional connectivity in the cancer interactome. Using our network-based method, we demonstrated that more than half of the point mutations in the GBM set and around 1/6^th^ of those in the OV set had some functional involvement in the corresponding cancers. While these cancers may involve different mutation mechanisms, the poorer results in the latter set are probably due to the different sequencing approaches used in compiling the two data sets. The GBM set was the very first set compiled for TCGA and features mutations from a limited set of only around 600 pre-selected genes with either known or suspected involvement in glioblastoma. Conversely, the OV set contains data from full-exome sequencing. The latter approach yielded a much greater proportion of passenger mutations, and this result should be primarily attributed to the less focused genomic approach rather than to a lower precision of the analysis *per se*. Applying our method to the copy number alterations in each of the two collections revealed between 300 and 600 driver CNA cases, depending on the applied confidence threshold.

**Figure 7:**
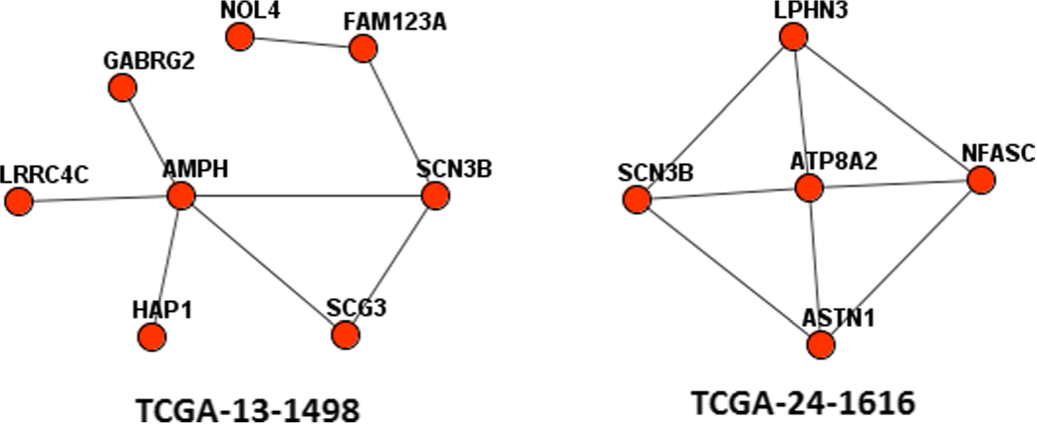
**The driver role of the sodium channel voltage-gated type III beta subunit SCN3B was likely implemented via network links to different, non-overlapping sets of point mutations in two OV genomes.** Red nodes indicate point mutations in the same tumor genome.

We reiterate that in the 1-vs-CPW tests any gene could be analyzed against third party pathway sets regardless of other genes in the somatic genome. On the contrary, the 1point-vs-MGS and 1CNA-vs-MGS procedures consider other alterations in the same genomes, i.e. they are genuinely local tests. Importantly, the local tests detected certain driver genes in novel genomic contexts (Figure 7). The applicability of these analyses will increase with the declining costs of full-exome and full-genome sequencing, growing confidence in the global network, and the progressive incorporation of data on methylation and germline variants etc.

A number of genes exhibited point mutations in some genomes, CNAs in others, and both in a third group. However, the 1-vs-CPW, 1point-vs-MGS, and 1CNA-vs-MGS scores of individual genes correlated with one-another quite well (Additional file 4: Figure S2). We take this to mean that in appropriate genomic contexts, a given gene may become a driver due to either a copy-number change or a point mutation.

Many existing methods for validating CNA drivers match observed copy numbers to expression of the affected gene. However, neighboring genes might have synchronized expression changes regardless of their driver activity. We did observe an overall correlation between CNA and expression level (median Spearman rank r = 0.2…0.4; see Additional file 4: Figure S3B, top pane). However, this was not a reliable indicator of driver status. Indeed, the correlations for known drivers such as those reported by Parsons et al. [5] and Vogelstein et al. [19] were not stronger than those for the whole set of studied genes (Additional file 4: Figure S3A). One common exception was EGFR, a driver with extraordinarily high amplification levels (up to 100-fold). In our network analysis, the likelihood of being identified as a driver was also not associated with the CNA-expression correlation (Additional file 4: Figure S3B, C, D). Relying on this weak overall correlation would have led to prohibitively high false negative rates. Akavia et al. [20] pointed out that many drivers should be less correlated with their copy numbers than passengers as a result of “selection pressure”, by which they presumably meant negative feedback in regulatory networks.

We also observed poor agreement between the tested sequence-based methods and NEA. On the other hand, there was significant correspondence between the NEA results on the one hand and both frequency-based estimates and lists of known cancer genes on the other hand.

The sensitivity of the 1point-vs-MGS procedure was dependent on the size of the analyzed MGS. No MGS with fewer than four genes produced any positive results (such samples were rare though). On the other hand, we observed a tendency for known, frequent drivers with strong mutation effects to occur more frequently in genomes with small MGS. The effects of these genes (TP53, RB1, PTEN) were probably strong enough to generate malignant phenotypes on their own. Despite their limited amenability to 1point-vs-MGS analysis, we did not overlook them in our combined procedure because they had many connections in cancer pathways. As a result, their driver roles were discovered in the 1-vs-CPW test. We also identified significant correlations between all the three types of NEA (Additional file 4: Figure S2).

Based on the demonstrated strengths and limitations of each approach, we argue that there is a large unoccupied niche for network enrichment analysis. In essence, the method requires information on the context of the somatic genome of interest, relevant pathways, and the connections between genes in the global network. It could be indispensable while integrating molecular mechanisms of cancer, in cases where large sample collections are unavailable, and when making clinical decisions concerning the treatment of specific individuals where being able to answer the question “What has gone wrong in this tissue?” would enable the selection of a tailored treatment to prevent further cancer progression.

## Methods

### Connectivity tests

This section describes the calculation of NEA Z scores as implemented in the script NEA.pl which we make publicly available at http://research.scilifelab.se/andrej_alexeyenko/downloads.html. The calculation is used in 1point-vs-MGS, 1CNA-vs-MGS, and 1-vs-CPW tests described in the Results.

For each potential driver gene *i*, the confidence of its functional relatedness to a group G was estimated using a *z*-score of network enrichment in edges that connect *i* and *G*:


where *n*_*iG*_ was the number of edges between *i* and any gene *j* : *j* ∈ *G*; *i ≠ j* found in the given network. In the procedures presented in Figure 1, 1point-vs-MGS assumed *i* ∈ *G*, whereas 1CNA-vs-MGS and 1-vs-MGS allowed any scenario. However, in the analyses *i* was treated as an external node in all three cases.

For procedure 1 from Figure 1, we tested the general coherence of G by counting:


In biological networks, the distribution of node degree (number of edges per node) follows a power law, i.e. it is very uneven: there are many nodes with either one or few links, while a few nodes have many (tens, hundreds, or even thousands) links. Thus the statistics would be strongly affected by the gene composition of particular sets. In order to obtain values for the expected (mean) number  and standard deviation *σ*_*iG*_, we randomized the network according to Maslov and Sneppen [56]. By systematically re-wiring network nodes, i.e. swapping edges between two nodes at a time, one can preserve node degrees and the total number of edges in the network. Hence, the biological content of the network is assumed to be removed while preserving its topological properties. The expected mean  and standard deviation *σ*_*iG*_ were determined after a sufficient number (25) of network randomizations. Then  was calculated for the randomized network in the same way as *n*_*iG*_ in the actual network. Alternatively, we used a network metric that counted indirect links, i.e. cases when nodes *i* and *j* shared a neighbour node in the network. In this case, *n*_*iG*_ and  summarized the shared neighbors in all possible *i-j* pairs (*j* ∈ *G*).

For the analysis, we considered only MGSs with at least three genes in each set, i.e. those amenable to NEA (which was 67.6% and 94.1% of all MGSs, respectively).

### Altered gene sets

We defined sets of individual-specific somatic point mutations (MGS) as all genes listed in TCGA maf tables for GBM and OV cancers, irrespective of their “Variant_Classification” label. Multiple mutations in the same gene were collapsed into one case per sample.

Copy number alterations were accepted as significant if the log_2_(copy number) value for the corresponding gene coding region was below 0.35.

### Functional gene sets

We took all of the pathways included in the KEGG database [57] (as of 21 Apr. 2010), and delineated the following three categories:

1. SIG, 68 pathways with signaling functionality, retrieved by using the KEGG04* mask, plus the group KEGG03320 “PPAR signaling pathway”
2. OTH, pathways other than signaling and disease ones, retrieved by using the KEGG00*, KEGG01*, KEGG02*, KEGG03* masks.
3. CAN, 15 cancer-related pathways, retrieved by using the KEGG052* mask, plus the following custom pathways:

Pancreatic cancer pathway [4],

Breast cancer pathway [1],

Colorectal cancer pathway [1],

Glioblastoma multiforme cancer pathway [5],

26 significantly frequent lung cancer drivers [3],

T cell homing on tumor pathway [58],

epithelial-mesenchymal transition (courtesy of S. Souchelnytskyi), and

tumor-specific pH-shift (courtesy of A. de Milito);

and 15 GO terms [59] that could be related to hallmarks of cancer [60]:

GO:0001525 “Angiogenesis”,

GO:0001666 “Response to hypoxia”,

GO:0002347 “Response to tumor cell”,

GO:0002418 “Immune response to tumor cell”,

GO:0005154 “EGFR binding”,

GO:0005161 “PDGFR binding”,

GO:0005164 “TNF binding”,

GO:0005520 “IGF binding”,

GO:0006915 “Apoptosis”,

GO:0007179 “TGFb pathway”,

GO:0017134 “FGF binding”,

GO:0032570 “Response to progesterone stimulus”,

GO:0032640 “TNF production”,

GO:0043120 “TNF binding”,

GO:0070848 “Response to growth factor stimulus”.

### Global networks for benchmarking and analysis

See Additional file 4: Table S1.

### Network benchmarks

The performance of different network versions in functional analysis has never previously been evaluated systematically to our knowledge. It is thus not clear which network should be used to test mutations. It is well recognized that data integration networks, despite their vast scope, have poor agreement with each other when judged by straightforward overlap. It is hard to say why this is on the global scale. However, it is fair to assume that both false positive and false negative rates are high in both resource types, i.e. many false edges will be present and many existing edges will be missed.

There are different ways of testing networks in terms of completeness, confidence, and information content. The most straightforward approach would be to count edges shared by two or more networks. However, pairs of networks generated by different computational methods would be hard to align and compare because of their different, often nonlinear edge weight relations and unequal densities. Benchmarking based on a percentage of “true” edges would require a gold standard network that would be either incomplete (e.g. the pathways of the KEGG database [57]) or abound in false positives (e.g. any network generated by computational data integration). As a way of accounting for the complex topologies generated by sophisticated analyses such as that implemented in the Ingenuity Pathway Analysis [Ingenuity® Systems, http://www.ingenuity.com], Lena et al. [61] developed a scoring system for comparing pathways. However even this method is limited to well-trusted network domains.

With the aim of avoiding both excessively simplistic and overly complex topological issues, we benchmarked networks using a method similar to that used for practical driver discovery, i.e. by their ability to recapitulate the known memberships of genes in functional groups (as illustrated in pane D of Figure 1). This network-based approach was both biologically transparent and amenable to significance estimation. Benchmarks that involved thousands of individual membership cases were visualized as ROC curves. Plotting the ROC curves required 1) positive (gold standard) test sets of functional groups, 2) negative sets, and 3) a variable parameter. These components are described in the following paragraph.

In the connectivity tests, the number of correctly identified FGS members at a given *z*-score threshold estimated the true positive rate. As *positive test sets*, we used the KEGG pathways [57]. The results obtained by using alternative pathway databases were overall similar to those obtained using the KEGG data, probably because these different resources utilized the same published experimental evidence. We preferred KEGG because it enabled simple and transparent classification of pathways into convenient categories : 1) signaling (SIG), 2) other basic (OTH), and a cancer-related (CAN) collection of pathways together with a number of biological processes based on Gene Ontology terms, and a few cancer pathways collected from the literature (described in the section on "Functional gene sets" above).

To estimate the false positive rates, we simulated *negative test sets* by replacing each actual gene member with a randomly picked gene with a matching node degree (network connectivity) value. The scales of the false positive and true positive axes were equal because each test on an actual FGS member was matched with one test on an allegedly false member. Some of the latter would have been previously unknown true members or “remote relatives” of the used pathways. These false positive estimates were thus probably too conservative, but still suitable for benchmarking.

NEA z-scores were employed as the *variable parameter* for ROC curve generation. For a single gene *i* that belonged to a group *G*, the individual NEA *z-*score conveyed enrichment in network connectivity between *i* and the rest of the group *G* (Figure 1B,C,D). Each NEA test attempted to reject a null hypothesis stating that “there is no relationship between gene *i* and *G*”. Counts of true positives versus false positives at decreasing *z*-score thresholds were thus used as Y and X coordinates for ROC curve construction. This test framework would be relatively robust to false positives and false negative edges in the global network because each test involved multiple edges. Edge weights were not utilized, i.e. each network was presented as a fixed-size graph defined at a certain edge confidence cutoff. This was important when merging networks generated by different methods. Another feature of the framework was that the ROC curves were terminated at the points of lowest formal significance (NEA *z* = 1.97, i.e. the two-tailed *p* = 0.01). For this reason, the area under ROC could not be used to compare curves. Instead, we compared sensitivity/specificity ratios at points where the false discovery rate (FDR) of NEA was 0.1. In addition to this criterion, we visually judged the ROC curves, requiring both convexity and sensitivity (based on the total number of recapitulated true members in all gene tests).

Using this framework, we evaluated a range of networks (detailed descriptions of the tested networks are given in the Additional file 4: Table S1). The most important results are presented in Figure 2 and Additional file 4: Figure S1. The first category included networks from large scale data integration (LSDI): versions of FunCoup (v. 1 and 2) and the last release (v.9) of the STRING database. We compiled and tested versions of these networks of different sizes and edge confidence values. Secondly, it was possible that specific co-expression in GBM and OV could be beneficial for cancer data analysis. For this reason, we evaluated so called relevance networks in which cancer-specific relationships (that were not considered in LSDI) were represented by correlation coefficients between gene expression profiles in GBM and OV. Finally, yet another way to obtain more specific gene networks was reverse engineering of regulatory (otherwise called causal) links from high-throughput cancer data. Two such networks were generated by us from the GBM and OV data (wir1 and wir.OV.0.5, see Additional file 4: Supplemental Methods), and one network for the ovarian cancer OV_TRANSFAC was obtained from the literature [62].

The benchmark demonstrated clear differences between the networks (Additional file 4: Figure S1). The first observation was that the LSDI networks were superior to all other alternatives, i.e. networks based on physical protein-protein interactions or co-expression relevance, reverse-engineered networks, and their unions. The levels of performance were relatively similar between the full LSDI networks, despite dramatic differences in their numbers of edges (which ranged from 1.4 to 4.6 million) and nodes (which ranged from 15.9 to 19.4 thousand genes). This similarity could be explained by the fact that the edges of lower confidence in both STRING and FunCoup networks had low experimental support. In other words, the network performance was mostly determined by core fractions of high confidence edges with extensive support from the literature, co-expression analysis etc. Further, we noted that the full STRING network clearly outperformed the FunCoup networks (Additional file 4: Figure S1A,C). However, it was known that the STRING network, unlike those generated by FunCoup, included information from all of the KEGG pathways by default. To perform a more fair comparison, we merged each of the networks with the full set of KEGG links and CORUM protein complex members and then equalized their numbers of edges by selecting the most highly ranked edges in each network. This procedure made the networks perform even more similarly (Additional file 4: Figure S1B). However FunCoup had a better ratio of sensitivity/(1 - specificity) (11…12 compared to around 9 for STRING). In this regard, STRING performed notably worse than the mammal-focused version of FunCoup FClim (in all benchmarks other than that relating to the OTH category). The final selection was made based on the most important, cancer-related category CAN (Figure 2). We hypothesize that the CAN analysis was to the particular disadvantage of STRING because it incorporates data from homologous genes in prokaryotic organisms, whereas FunCoup utilized only eukaryotic evidence and strictly defined orthologs. FClim had the highest fraction of evidence from human and other mammalian (mouse and rat) data sources. Finally, we merged the higher confidence network version FClim_HC2 with curated functional links from CORUM (protein complex membership, [63]), Phosphosite (kinase-substrate pairs, [64]), KEGG (pathways and protein complexes, [57]), MSigDB (transcription factor-regulated gene pairs, [58]), and our reverse-engineered network wir1. The resulting network merged6_and_wir1_HC2 had the best performance, i.e. the highest sensitivity/specificity ratio and at least marginally higher convexity and total sensitivity than any other network.

### Coherence of genome-specific sets of point mutations

We evaluated the functional coherence of point mutations, i.e. MGS members within each somatic cancer genome. By considering direct links, significant coherence was detected in 24 out of 98 (GBM) and in 6 out of 80 (OV) MGS (the analysis was limited to samples with both point mutation and copy number data, which was important in the following steps). Furthermore, we quantified this coherence via indirect links by quantifying shared neighbors between two genes of interest. This analysis greatly increased the number of MGS exhibiting significant coherence, to 46 out of 98 and 63 out of 80 in GBM and OV, respectively.

### Normalization of 1-vs-CPW analysis

The network analysis based on known cancer pathways (1-vs-CPW, Figure 1D) included tests against all of the FGS listed in the section on “Functional gene sets” under the CAN category. Due to the overlap of member genes, these tests were highly correlated, and their summative estimates required adjustment. One of these FGS, KEGG05200, was a super-pathway which combined genes from 14 specific KEGG cancer pathways (254 out of 375 genes, plus 11 genes that were unique to KEGG05200). We compared the two approaches: 1) using KEGG05200 alone and 2) using summed NEA z-scores from all cancer FGS. The results obtained in both cases were significantly consistent, yielding p-values in the formally acceptable range (*p* < 0.01). The approach using KEGG05200 alone had the advantage of providing directly interpretable p-values and FDR data. However, for the sake of higher sensitivity, it was desirable to use all possible FGSs. For example, at a confidence cut-off that corresponded to maximal concordance of the two alternatives, 20 actual members of KEGG05200 were not detected when using this pathway alone but were successfully detected based on the summed scores for all cancer FGSs. To regularize the summed NEA *z*-scores from multiple FGSs, they were divided by a factor of 10.16, which was the linear fit coefficient of KEGG05200 against the sum of other cancer FGSs. Next, we established that an NEA FDR of 0.1 corresponded to an NEA *z*-score of 30.17/10.16 = 2.97. Hence in the following analysis, we accepted NEA *z/*10.16 = 2.97 as the lowest significance cut-off.

### Combining p-values from multiple tests

Fisher’s combined p-value [65] assumes the calculation of a chi-squared value:


which can then be routinely converted to a p-value that summarizes multiple tests.

Using this formula, we calculated different combinations of p-values. Fisher’s formula assumes that the individual tests to be combined are independent, which cannot be entirely guaranteed in our analysis. As we were not aware of any unbiased procedures to adjust for multiple testing in this context, the combined p-values were used only for ranking and prioritization.

### CNA genes

The following procedure was applied for each gene with multiple CNA cases (requiring absolute values of log_2_(copy number) > 0.35 in at least 3 genomes within either the GBM or the OV dataset):

1. First, we selected only CNA genes that significantly co-occurred with any point mutations. Cases of co-occurrence of the CNA gene with a point mutation gene (requiring *p*_*Fisher’s exact test*_ < 0.01) were combined as follows:
2. For each copy number-altered gene, we had a NEA z-score and a corresponding p-value from the 1CNA-vs-MGS analysis from each of N_own_MGS_ genomes. These *p*_NEA, 1CNA-vs-MGS_ were combined via chi-squared values:
3. NEA z-scores from 1-vs-CPW analyses for individual cancer pathways were positively correlated with each other. Hence they were integrated as a linear sum, then divided with the correction factor 10.16 (see “Normalization of 1-vs-CPW analysis”), and converted to single values *p*_*NEA,cancer_pathways*_.
4. P-values from steps 1,2 and 3 were combined as:

### Genes with somatic point mutations

The mutation co-occurrence and 1CNA-vs-MGS analyses were not applicable here. Similarly to the above described, we combined two relevant types of p-values:


### Detection of functional consequences of mutations with sequence-based tools

Several sequence-based methods for assessing the effects of mutations on protein function have been developed. We submitted input data for the GBM and OV sets (as described below) and obtained output from the public web servers Mutation Assessor [51], PolyPhen-2 [52, 53] and SIFT [54].

The Mutation Assessor web server (version 2.0 http://mutationassessor.org/) used database versions Pfam 25 (November 2011), PDB (January 2012), RefSeq release 54, UniProtKB/Swiss-Prot and UniProtKB/TrEMBL as of July 2012. The information is derived from aligned families and sub-families of sequence homologs within and between species using combinatorial entropy formalism to calculate a functional impact score.

PolyPhen-2 web server (http://genetics.bwh.harvard.edu/pph2/) used protein sequences from UniProtKB/UniRef as of December 2011 and protein structures from PDB/DSSP Snapshot as of 3 January 2012. The probabilistic classifier of PolyPhen-2 used the HumDiv model to predict possible impacts of amino acid substitutions on the protein structure and function based on such features as sequence, phylogenetic, and structural information.

The SIFT server was accessed via http://sift.bii.a-star.edu.sg/www/SIFT_chr_coords_submit.html. SIFT does not consider protein structures to assess consequences of amino acid changes; instead, it uses a sequence conservation approach to distinguish between intolerable and tolerable amino acid substitutions and predict their impact on protein function.

The sets of point mutations in GBM and OV were compiled from the TCGA maf files, whose data was extracted in the following format: <chromosome>, <position>, <reference allele>, <substituted allele>. The functional effects of amino acid substitutions were predicted using NCBI build 36 of the human genome. We applied the score thresholds suggested by the authors of each method. Mutation Assessor classified variants into four classes (high impact, medium impact, low impact, and neutral). In our comparison, high- and medium-impact predictions were assigned a deleterious phenotype while other mutations were considered neutral. PolyPhen-2 provided three prediction classes (benign, possibly damaging, and probably damaging). In our comparison, ‘benign’ was assumed to represent a neutral phenotype and the other two categories were assigned to deleterious phenotypes. SIFT quantified tolerated and deleterious effects via a probability that was normalized by amino acid class. Values below 0.05 were considered deleterious; otherwise the mutations were deemed neutral. All the three tools could leave a fraction of mutations without any prediction. In our comparisons, such mutations were included with the negative test results, i.e. were considered neutral.

## Availability of supporting data

The supporting data to this article are included as additional files (probabilistic estimates of the driver analysis and chromosomal maps of copy number analysis). The software for the analysis as well as the global network of functional couplings are publicly available at http://research.scilifelab.se/andrej_alexeyenko/downloads.html.

## Electronic supplementary material

Additional file 1: **[Drivers.xlsx] contains the results of probabilistic analysis of both point and copy number mutations in the GBM and OV genomes.** The first two sheets contain systematic evaluation of all the mutations, whereas the latter two sheets present single-genome examples. (XLSX 2 MB)

Additional file 2: **[GBM.CNA_and_M2CH.alongChromosomes.v7.pdf] contains a graphical representation of copy number driver analysis along the chromosomes in** ***glioblastoma multiforme.*** (PDF 8 MB)

Additional file 3: **[OV.CNA_and_M2CH.alongChromosomes.v7.pdf] contains a graphical representation of copy number driver analysis along the chromosomes in ovarian carcinoma.** (PDF 5 MB)

Additional file 4: **[Drivers_in_glioblastoma.Supplementary.July29.docx] contains supplementary methods (reverse engineering of network wir1), tables (the descriptions of benchmarked networks and the** **re-analysis** **of results from Ciriello et al.** **[** [29]**]) as well as the supplementary figures.** (DOCX 744 KB)

## References

[1] Sjöblom T, Jones S, Wood LD, Parsons DW, Lin J, Barber TD, Mandelker D, Leary RJ, Ptak J, Silliman N, Szabo S, Buckhaults P, Farrell C, Meeh P, Markowitz SD, Willis J, Dawson D, Willson JK, Gazdar AF, Hartigan J, Wu L, Liu C, Parmigiani G, Park BH, Bachman KE, Papadopoulos N, Vogelstein B, Kinzler KW, Velculescu VE (2006). The consensus coding sequences of human breast and colorectal cancers. Science.

[2] Greenman C, Stephens P, Smith R, Dalgliesh GL, Hunter C, Bignell G, Davies H, Teague J, Butler A, Stevens C, Edkins S, O’Meara S, Vastrik I, Schmidt EE, Avis T, Barthorpe S, Bhamra G, Buck G, Choudhury B, Clements J, Cole J, Dicks E, Forbes S, Gray K, Halliday K, Harrison R, Hills K, Hinton J, Jenkinson A, Jones D (2007). Patterns of somatic mutation in human cancer genomes. Nature.

[3] Ding L, Getz G, Wheeler DA, Mardis ER, McLellan MD, Cibulskis K, Sougnez C, Greulich H, Muzny DM, Morgan MB, Fulton L, Fulton RS, Zhang Q, Wendl MC, Lawrence MS, Larson DE, Chen K, Dooling DJ, Sabo A, Hawes AC, Shen H, Jhangiani SN, Lewis LR, Hall O, Zhu Y, Mathew T, Ren Y, Yao J, Scherer SE, Clerc K (2008). Somatic mutations affect key pathways in lung adenocarcinoma. Nature.

[4] Jones S, Zhang X, Parsons DW, Lin JC, Leary RJ, Angenendt P, Mankoo P, Carter H, Kamiyama H, Jimeno A, Hong SM, Fu B, Lin MT, Calhoun ES, Kamiyama M, Walter K, Nikolskaya T, Nikolsky Y, Hartigan J, Smith DR, Hidalgo M, Leach SD, Klein AP, Jaffee EM, Goggins M, Maitra A, Iacobuzio-Donahue C, Eshleman JR, Kern SE, Hruban RH (2008). Core signaling pathways in human pancreatic cancers revealed by global genomic analyses. Science.

[5] Parsons DW, Jones S, Zhang X, Lin JC, Leary RJ, Angenendt P, Mankoo P, Carter H, Siu IM, Gallia GL, Olivi A, McLendon R, Rasheed BA, Keir S, Nikolskaya T, Nikolsky Y, Busam DA, Tekleab H, Diaz LA, Hartigan J, Smith DR, Strausberg RL, Marie SK, Shinjo SM, Yan H, Riggins GJ, Bigner DD, Karchin R, Papadopoulos N, Parmigiani G (2008). An integrated genomic analysis of human glioblastoma multiforme. Science.

[6] Huang S, Ernberg I, Kauffman S (2009). Cancer attractors: a systems view of tumors from a gene network dynamics and developmental perspective. Semin Cell Dev Biol.

[7] Krause DS, Van Etten RA (2005). Tyrosine kinases as targets for cancer therapy. N Engl J Med.

[8] Nelander S, Wang W, Nilsson B, She Q-B, Pratilas C, Rosen N, Gennemark P, Sander C (2008). Models from experiments: combinatorial drug perturbations of cancer cells. Mol Syst Biol.

[9] Basanta D, Gatenby RA, Anderson AR (2012). Exploiting evolution to treat drug resistance: combination therapy and the double bind. Mol Pharm.

[10] Lee MJ, Ye AS, Gardino AK, Heijink AM, Sorger PK, MacBeath G, Yaffe MB (2012). Sequential application of anticancer drugs enhances cell death by rewiring apoptotic signaling networks. Cell.

[11] Kraggerud SM, Hoei-Hansen CE, Alagaratnam S, Skotheim RI, Abeler VM, Rajpert-De Meyts E, Lothe RA (2013). Molecular characteristics of malignant ovarian germ cell tumors and comparison with testicular counterparts: implications for pathogenesis. Endocr Rev.

[12] Carter H, Chen S, Isik L, Tyekucheva S, Velculescu VE, Kinzler KW, Vogelstein B, Karchin R (2009). Cancer-specific high-throughput annotation of somatic mutations: computational prediction of driver missense mutations. Cancer Res.

[13] Cancer Genome Atlas Research Network (2011). Integrated genomic analyses of ovarian carcinoma. Nature.

[14] Cerutti P, Hussain P, Pourzand C, Aguilar F (1994). Mutagenesis of the H-ras protooncogene and the p53 tumor suppressor gene. Cancer Res.

[15] Stephens PJ, McBride DJ, Lin ML, Varela I, Pleasance ED, Simpson JT, Stebbings LA, Leroy C, Edkins S, Mudie LJ, Greenman CD, Jia M, Latimer C, Teague JW, Lau KW, Burton J, Quail MA, Swerdlow H, Churcher C, Natrajan R, Sieuwerts AM, Martens JW, Silver DP, Langerød A, Russnes HE, Foekens JA, Reis-Filho JS, Van ‘t Veer L, Richardson AL, Børresen-Dale AL (2009). Complex landscapes of somatic rearrangement in human breast cancer genomes. Nature.

[16] Kaminker JS, Zhang Y, Watanabe C, Zhang Z (2007). Canpredict: a computational tool for predicting cancer-associated missense mutations. Nucleic Acids Res.

[17] Torkamani A, Schork NJ (2008). Prediction of cancer driver mutations in protein kinases. Cancer Res.

[18] Leary RJ, Lin JC, Cummins J, Boca S, Wood LD, Parsons DW, Jones S, Sjöblom T, Park BH, Parsons R, Willis J, Dawson D, Willson JK, Nikolskaya T, Nikolsky Y, Kopelovich L, Papadopoulos N, Pennacchio LA, Wang TL, Markowitz SD, Parmigiani G, Kinzler KW, Vogelstein B, Velculescu VE (2008). Integrated analysis of homozygous deletions, focal amplifications, and sequence alterations in breast and colorectal cancers. Proc Natl Acad Sci U S A.

[19] Vogelstein B, Papadopoulos N, Velculescu VE, Zhou S, Diaz LA, Kinzler KW (2013). Cancer genome landscapes. Science.

[20] Akavia UD, Litvin O, Kim J, Sanchez-Garcia F, Kotliar D, Causton HC, Pochanard P, Mozes E, Garraway LA, Pe’er D (2010). An integrated approach to uncover drivers of cancer. Cell.

[21] Beroukhim R, Getz G, Nghiemphu L, Barretina J, Hsueh T, Linhart D, Vivanco I, Lee JC, Huang JH, Alexander S, Du J, Kau T, Thomas RK, Shah K, Soto H, Perner S, Prensner J, Debiasi RM, Demichelis F, Hatton C, Rubin MA, Garraway LA, Nelson SF, Liau L, Mischel PS, Cloughesy TF, Meyerson M, Golub TA, Lander ES, Mellinghoff IK (2007). Assessing the significance of chromosomal aberrations in cancer: methodology and application to glioma. Proc Natl Acad Sci U S A.

[22] Ciriello G, Miller ML, Aksoy BA, Senbabaoglu Y, Schultz N, Sander C (2013). Emerging landscape of oncogenic signatures across human cancers. Nat Genet.

[23] The International Cancer Genome Consortium (2010). International network of cancer genome projects. Nature.

[24] Ideker T, Sharan R (2008). Protein networks in disease. Genome Res.

[25] Alexeyenko A, Sonnhammer EL (2009). Global networks of functional coupling in eukaryotes from comprehensive data integration. Genome Res.

[26] The Cancer Genome Atlas Research Network (2008). Comprehensive genomic characterization defines human glioblastoma genes and core pathways. Nature.

[27] Torkamani A, Schork NJ (2009). Identification of rare cancer driver mutations by network reconstruction. Genome Res.

[28] Cerami E, Demir E, Schultz N, Taylor BS, Sander C (2010). Automated network analysis identifies core pathways in glioblastoma. PLoS One.

[29] Ciriello G, Cerami E, Sander C, Schultz N (2012). Mutual exclusivity analysis identifies oncogenic network modules. Genome Res.

[30] Gu Y, Wang H, Qin Y, Zhang Y, Zhao W, Qi L, Zhang Y, Wang C, Guo Z (2013). Network analysis of genomic alteration profiles reveals co-altered functional modules and driver genes for glioblastoma. Mol BioSyst.

[31] Babaei S, Hulsman M, Reinders M, de Ridder J (2013). Detecting recurrent gene mutation in interaction network context using multi-scale graph diffusion. BMC Bioinformatics.

[32] Alexeyenko A, Lee W, Pernemalm M, Guegan J, Dessen P, Lazar V, Lehtiö J, Pawitan Y (2012). Network enrichment analysis: extension of gene-set enrichment analysis to gene networks. BMC Bioinformatics.

[33] Alexeyenko A, Wassenberg DM, Lobenhofer EK, Yen J, Linney E, Sonnhammer ELL, Meyer JN (2010). Dynamic zebrafish interactome reveals transcriptional mechanisms of dioxin toxicity. PLoS One.

[34] McCormack T, Frings O, Alexeyenko A, Sonnhammer EL (2013). Statistical assessment of crosstalk enrichment between gene groups in biological networks. PLoS One.

[35] Reynolds CA, Hong MG, Eriksson UK, Blennow K, Wiklund F, Johansson B, Malmberg B, Berg S, Alexeyenko A, Grönberg H, Gatz M, Pedersen NL, Prince JA (2011). Genetic association of sequence variants near AGER/NOTCH4 and dementia. J Alzheimers Dis.

[36] Hong MG, Alexeyenko A, Lambert JC, Amouyel P, Prince JA (2010). Genome-wide pathway analysis implicates intracellular transmembrane protein transport in Alzheimer disease. J Hum Genet.

[37] Bennet AM, Reynolds CA, Eriksson UK, Hong MG, Blennow K, Gatz M, Alexeyenko A, Pedersen NL, Prince JA (2011). Genetic association of sequence variants near AGER/NOTCH4 and dementia. J Alzheimers Dis.

[38] Navlakha S, Kingsford C (2010). The power of protein interaction networks for associating genes with diseases. Bioinformatics.

[39] Jansen R, Yu H, Greenbaum D, Kluger Y, Krogan NJ, Chung S, Emili A, Snyder M, Greenblatt JF, Gerstein M (2003). Bayesian networks approach for predicting protein–protein interactions from genomic data. Science.

[40] Troyanskaya OL, Dolinski K, Owen AB, Altman RB, Botstein DA (2003). Bayesian network for combining heterogeneous data sources for gene function prediction (in Saccharomyces cerevisiae). Proc Natl Acad Sci.

[41] Lee I, Date SV, Adai AT, Marcotte EM (2004). A probabilistic functional network of yeast genes. Science.

[42] von Mering C, Jensen LJ, Snel B, Hooper SD, Krupp M, Foglierini M, Jouffre N, Huynen MA, Bork P (2005). STRING: Known and predicted protein–protein associations, integrated and transferred across organisms. Nucleic Acids Res.

[43] Mammoto T, Jiang A, Jiang E, Panigrahy D, Kieran MW, Mammoto A (2013). Role of collagen matrix in tumor angiogenesis and glioblastoma multiforme progression. Am J Pathol.

[44] Toledo LI, Altmeyer M, Rask MB, Lukas C, Larsen DH, Povlsen LK, Bekker-Jensen S, Mailand N, Bartek J, Lukas J (2013). ATR prohibits replication catastrophe by preventing global exhaustion of RPA. Cell.

[45] Kim SY, Dunn IF, Firestein R, Gupta P, Wardwell L, Repich K, Schinzel AC, Wittner B, Silver SJ, Root DE, Boehm JS, Ramaswamy S, Lander ES, Hahn WC (2010). CK-epsilon is required for breast cancers dependent on beta-catenin activity. PLoS One.

[46] Yang WS, Stockwell BR (2008). Inhibition of casein kinase 1-epsilon induces cancer-cell-selective, PERIOD2-dependent growth arrest. Genome Biol.

[47] Nardinocchi L, Puca R, Givol D, D’Orazi G (2010). HIPK2-A therapeutical target to be (re)activated for tumor suppression: Role in p53 activation and HIF-1alpha inhibition. Cell Cycle.

[48] Choi YL, Bocanegra M, Kwon MJ, Shin YK, Nam SJ, Yang JH, Kao J, Godwin AK, Pollack JR (2010). LYN is a mediator of epithelial-mesenchymal transition and a target of dasatinib in breast cancer. Cancer Res.

[49] Krasnoperov V, Kumar SR, Ley E, Li X, Scehnet J, Liu R, Zozulya S, Gill PS (2010). Novel EphB4 monoclonal antibodies modulate angiogenesis and inhibit tumor growth. Am J Pathol.

[50] Löw S, Vougioukas VI, Hielscher T, Schmidt U, Unterberg A, Halatsch ME (2008). Pathogenetic pathways leading to glioblastoma multiforme: association between gene expressions and resistance to erlotinib. Anticancer Res.

[51] Reva B, Antipin Y, Sander C (2011). Predicting the functional impact of protein mutations: application to cancer genomics. Nucleic Acids Res.

[52] Adzhubei IA, Schmidt S, Peshkin L, Ramensky VE, Gerasimova A, Bork P, Kondrashov AS, Sunyaev SR (2010). A method and server for predicting damaging missense mutations. Nat Methods.

[53] Adzhubei I, Jordan DM, Sunyaev SR (2013). Predicting functional effect of human missense mutations using PolyPhen-2. Curr Protoc Hum Genet.

[54] Ng PC, Henikoff S (2003). SIFT: Predicting amino acid changes that affect protein function. Nucleic Acids Res.

[55] Losman JA, Kaelin WG (2013). What a difference a hydroxyl makes: mutant IDH,(R)-2-hydroxyglutarate, and cancer. Genes Dev.

[56] Maslov S, Sneppen K (2002). Specificity and stability in topology of protein networks. Science.

[57] Kanehisa M, Goto S, Kawashima S, Nakaya A (2002). The KEGG databases at GenomeNet. Nucleic Acids Res.

[58] Liberzon A, Subramanian A, Pinchback R, Thorvaldsdóttir H, Tamayo P, Mesirov JP (2011). Molecular signatures database (MSigDB) 3.0. Bioinformatics.

[59] Ashburner M, Ball CA, Blake JA, Botstein D, Butler H, Cherry JM, Davis AP, Dolinski K, Dwight SS, Eppig JT, Harris MA, Hill DP, Issel-Tarver L, Kasarskis A, Lewis S, Matese JC, Richardson JE, Ringwald M, Rubin GM, Sherlock G (2000). Gene ontology: tool for the unification of biology: The Gene Ontology Consortium. Nat Genet.

[60] Hanahan D, Weinberg RA (2011). Hallmarks of cancer: the next generation. Cell.

[61] Lena PD, Wu G, Martelli PL, Casadio R, Nardini C (2013). MIMO: an efficient tool for molecular interaction maps overlap. BMC Bioinformatics.

[62] di Bernardo D, Thompson MJ, Gardner TS, Chobot SE, Eastwood EL, Wojtovich AP, Elliott SJ, Schaus SE, Collins JJ (2005). Chemogenomic profiling on a genome-wide scale using reverse-engineered gene networks. Nat Biotechnol.

[63] Ruepp A, Waegele B, Lechner M, Brauner B, Dunger-Kaltenbach I, Fobo G, Frishman G, Montrone C, Mewes HW (2010). CORUM: the comprehensive resource of mammalian protein complexes–2009. Nucleic Acids Res.

[64] Hornbeck PV, Kornhauser JM, Tkachev S, Zhang B, Skrzypek E, Murray B, Latham V, Sullivan M (2012). PhosphoSitePlus: a comprehensive resource for investigating the structure and function of experimentally determined post-translational modifications in man and mouse. Nucleic Acids Res.

[65] Fisher RA (1925). Statistical methods for research workers.

