# Supplementary figures and images for: Distinguishing between driver and passenger mutations in individual cancer genomes by network enrichment analysis
Source: BMC Bioinformatics. 2014 Sep 19;15(1):308. doi: 10.1186/1471-2105-15-308 (PMC4262241; doi:10.1186/1471-2105-15-308)

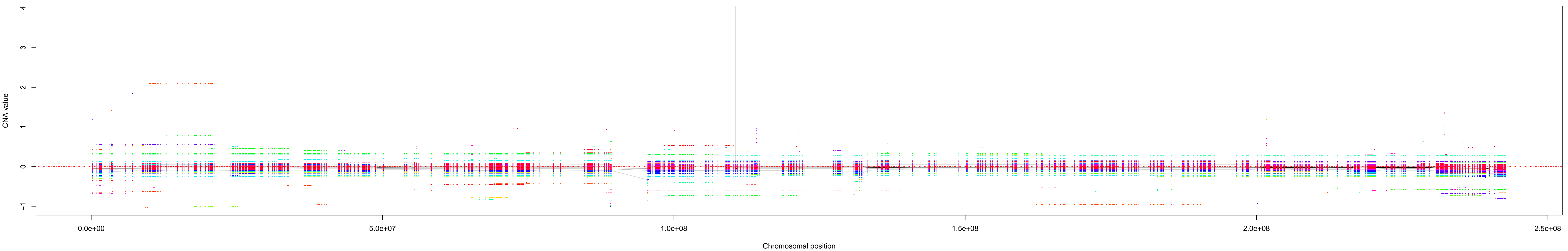

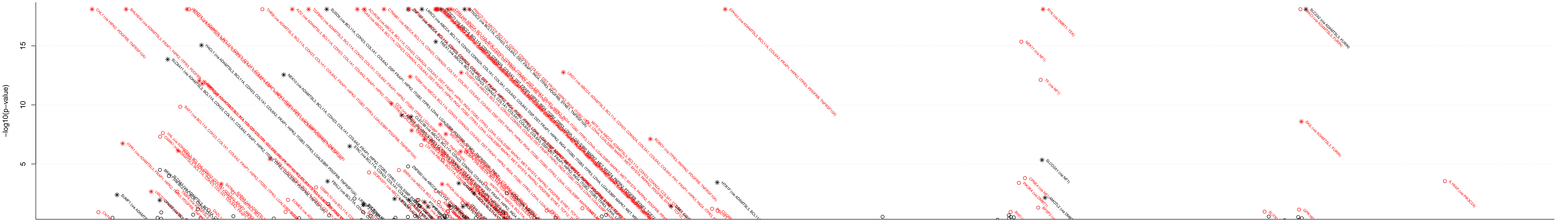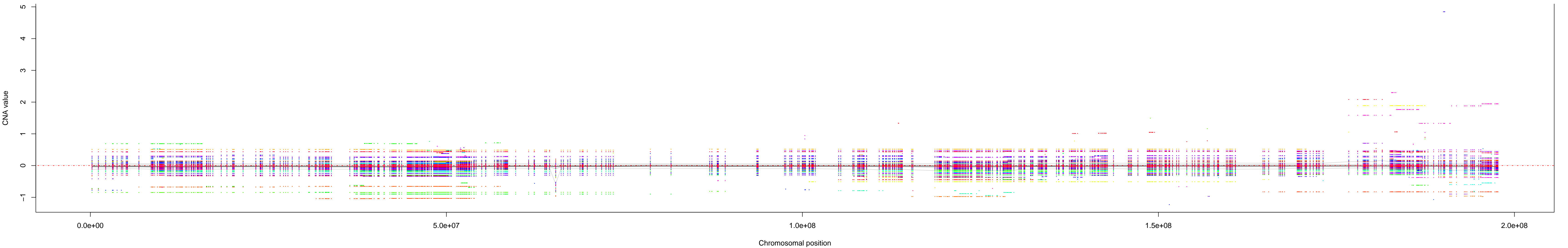

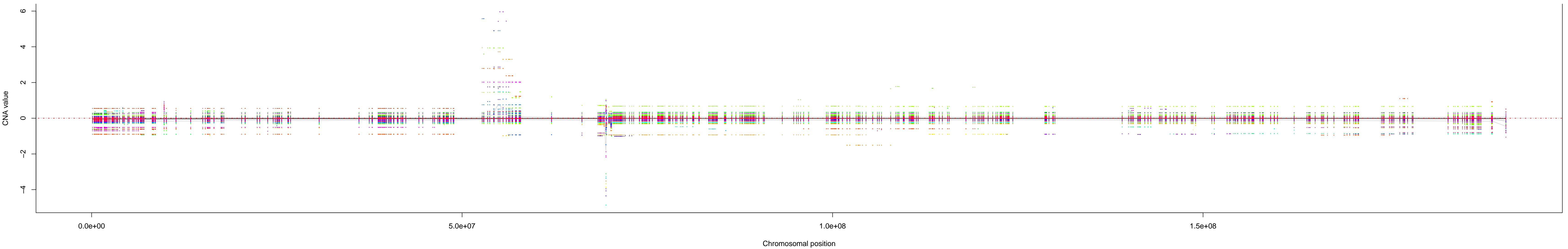

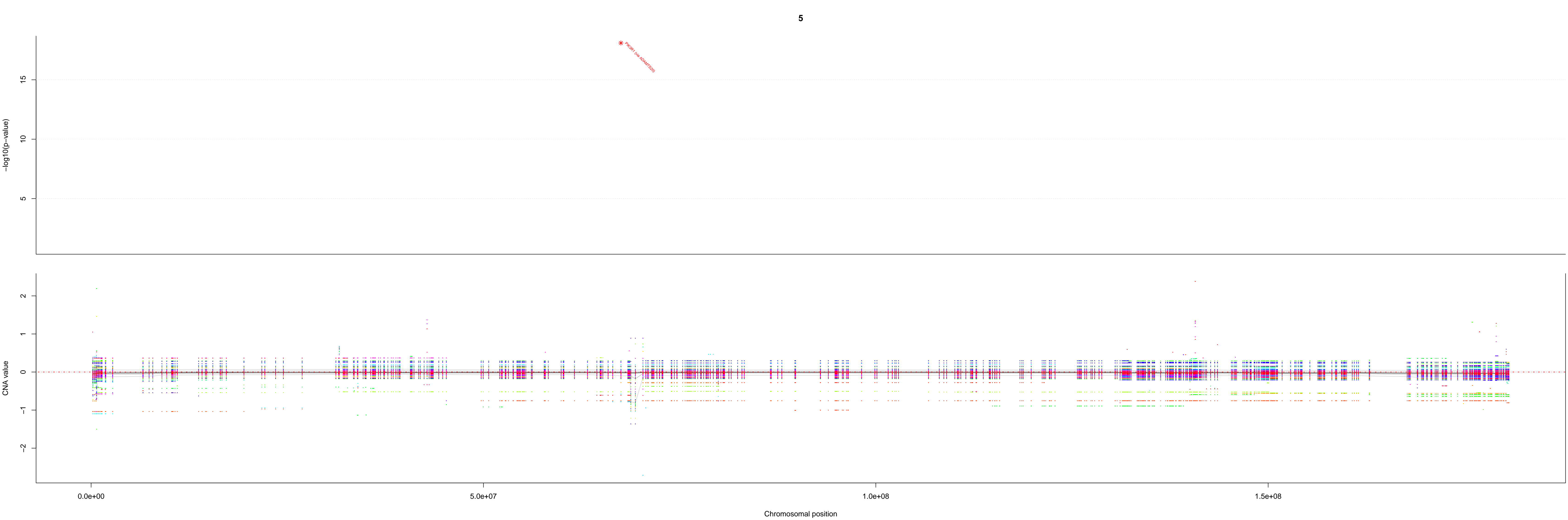

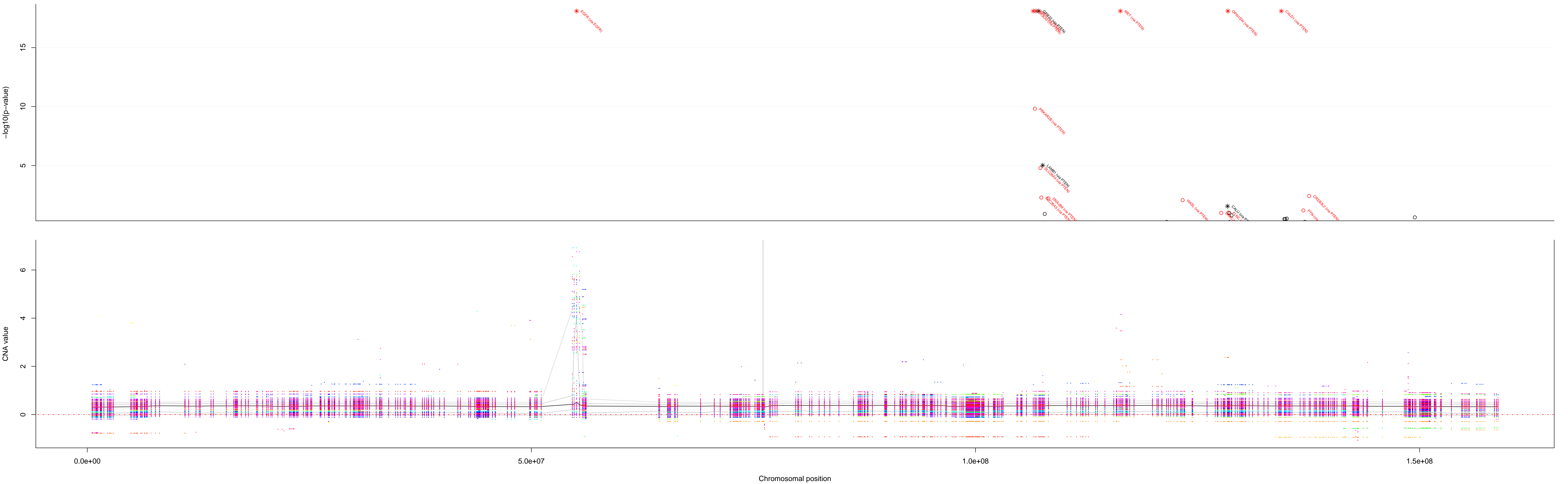

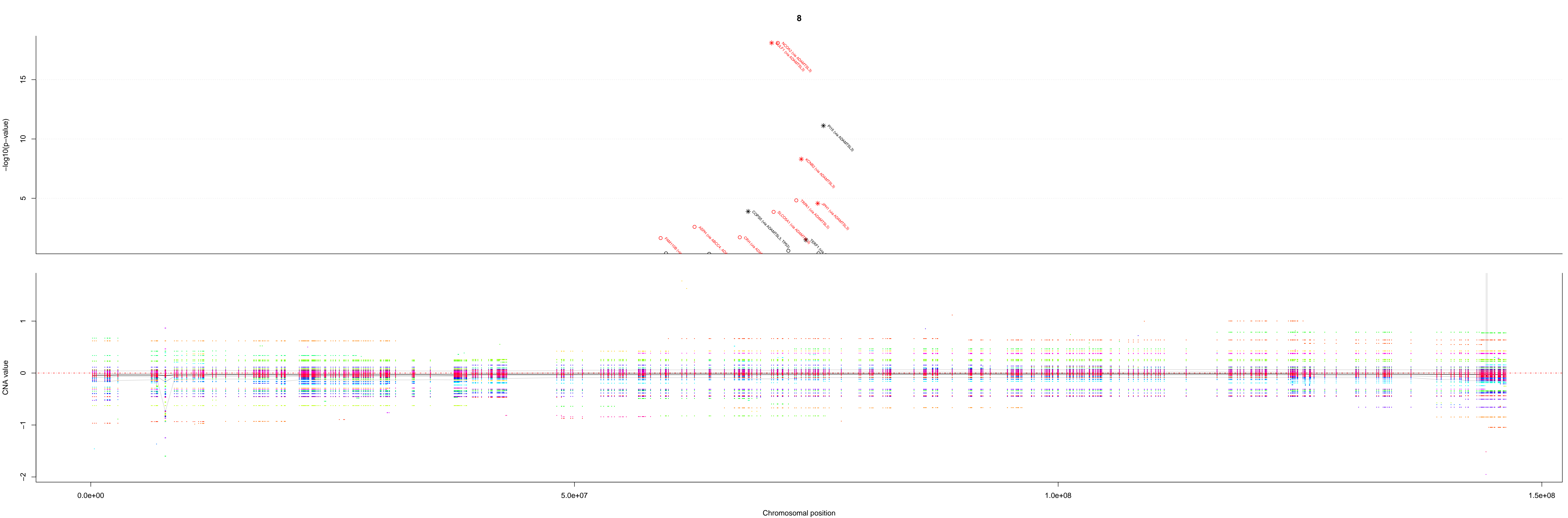

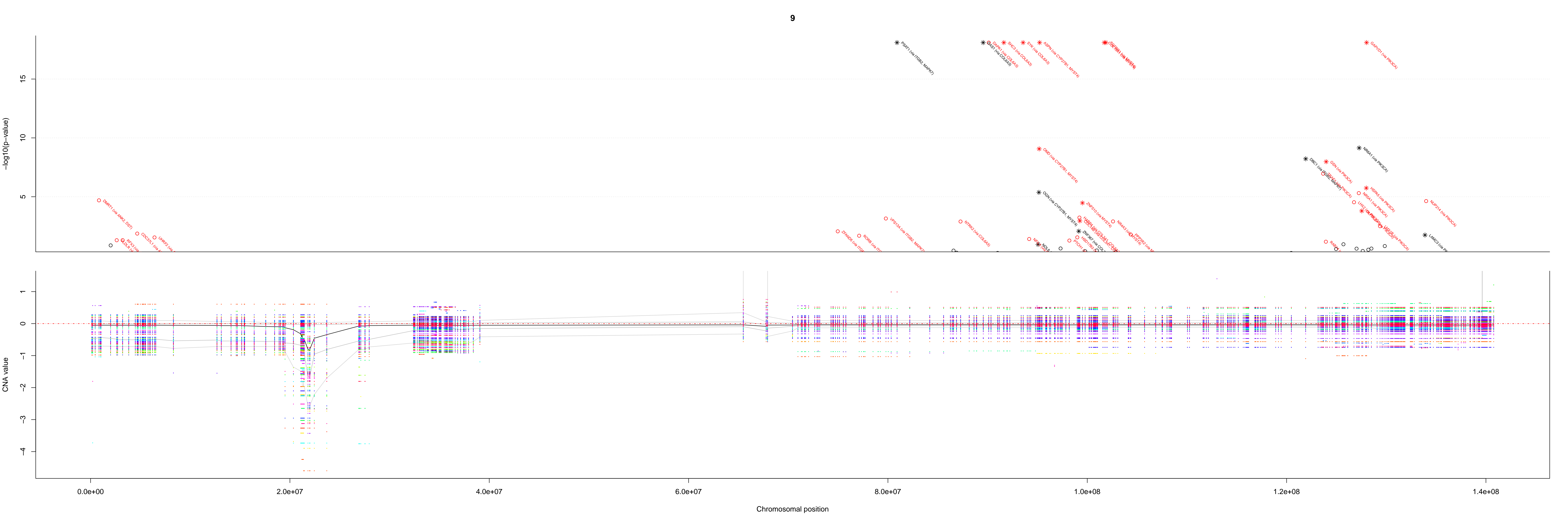

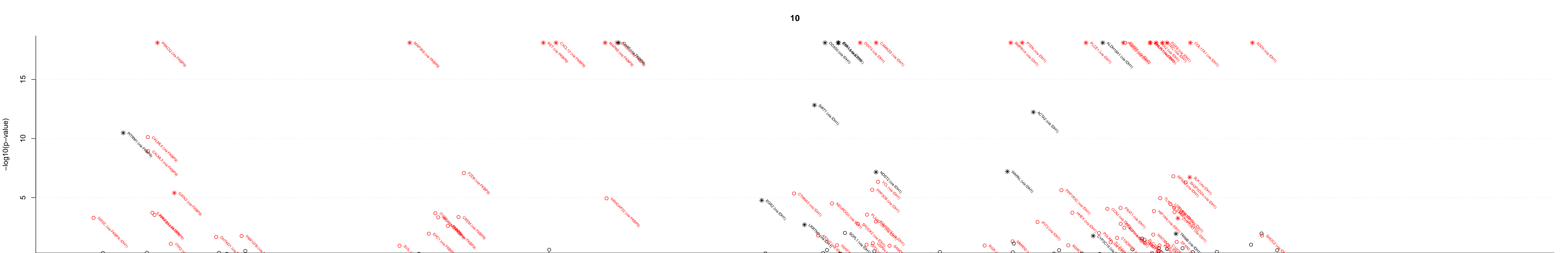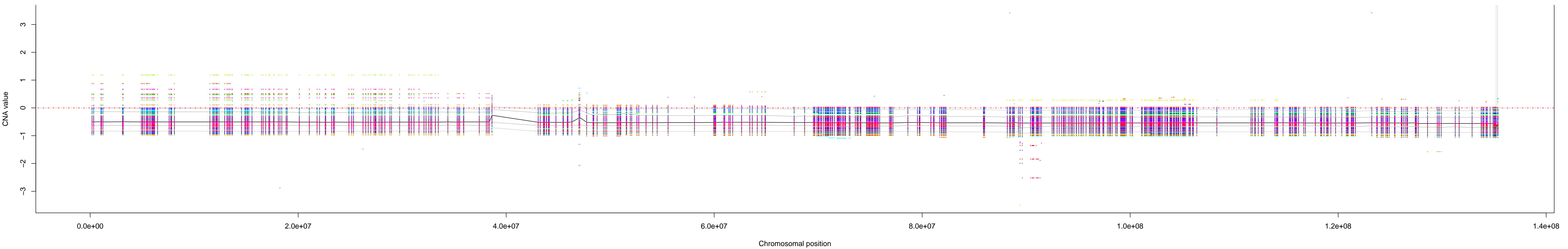

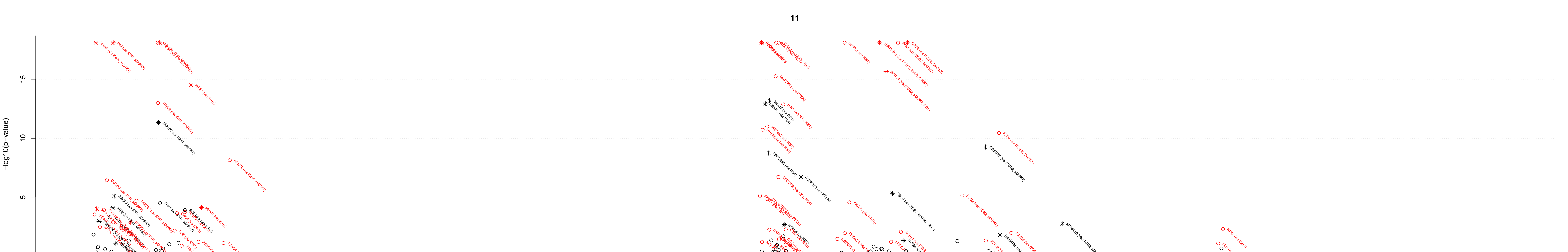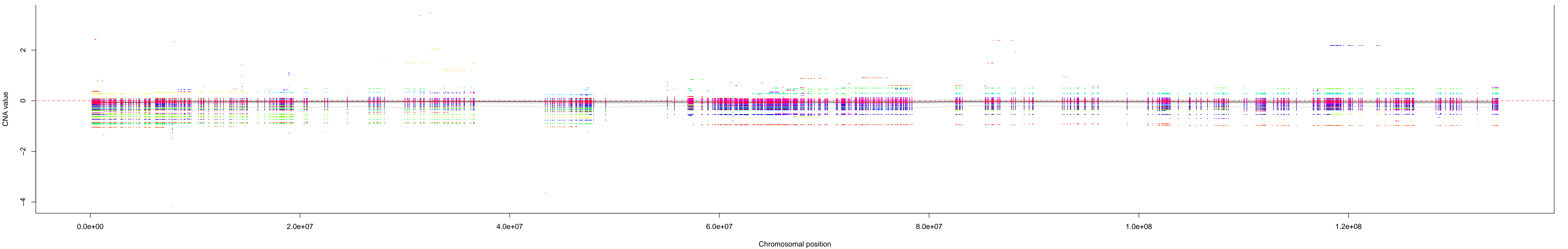

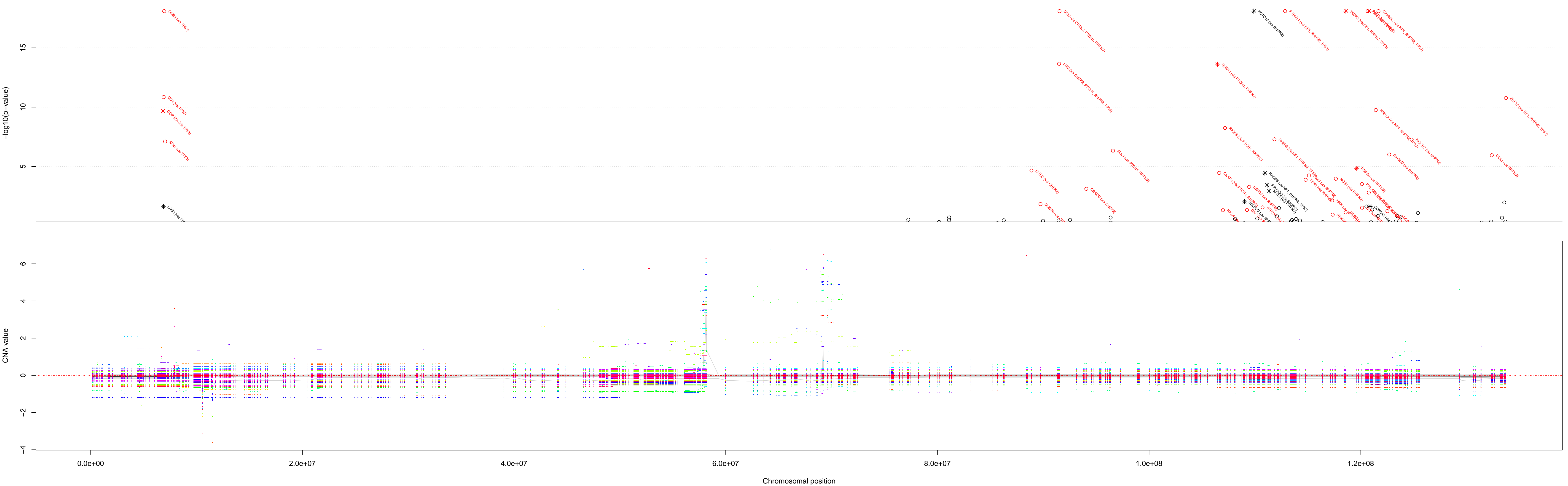

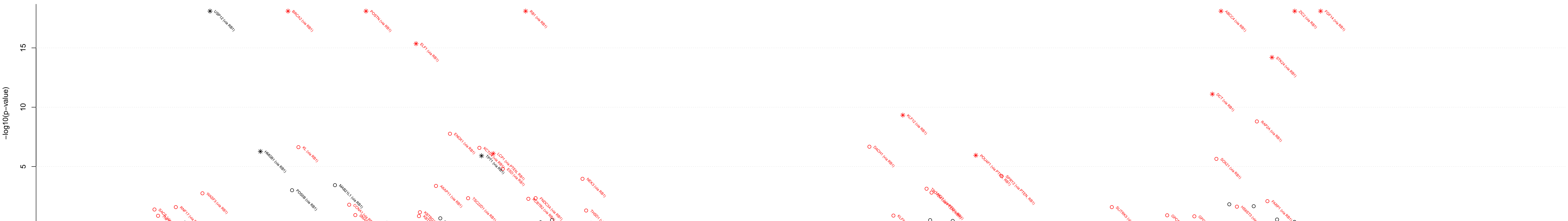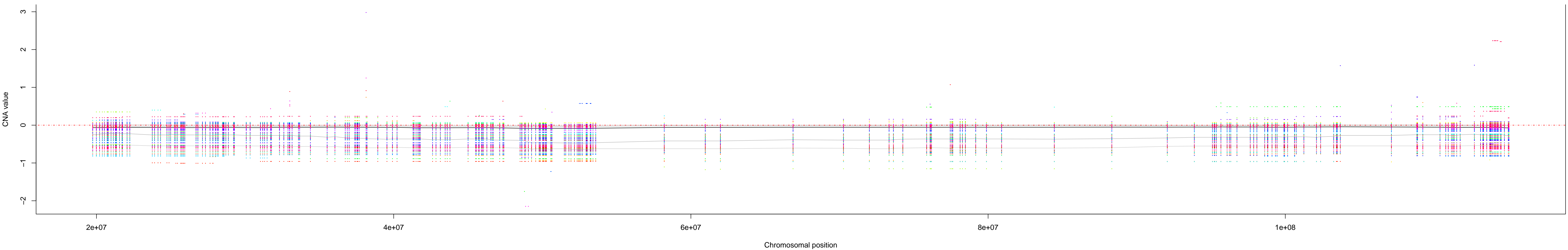

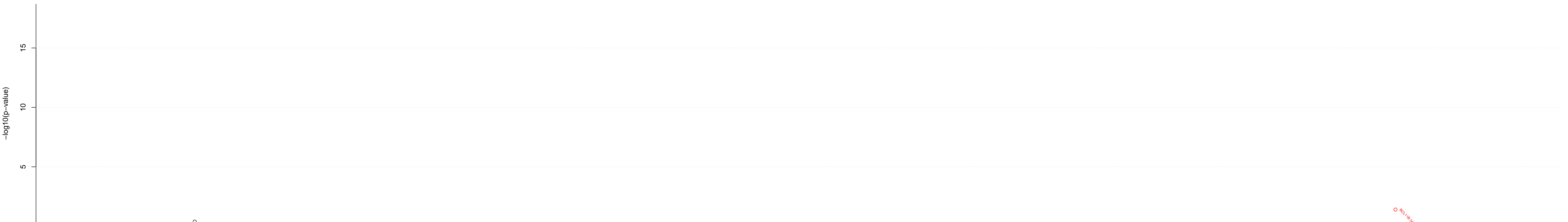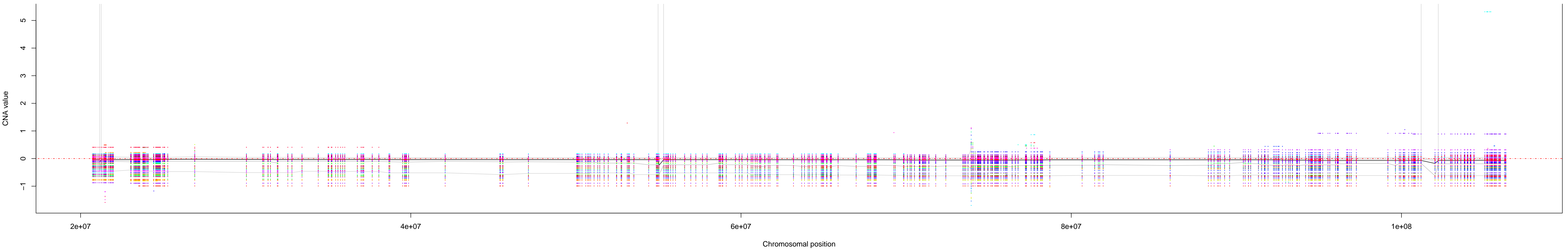

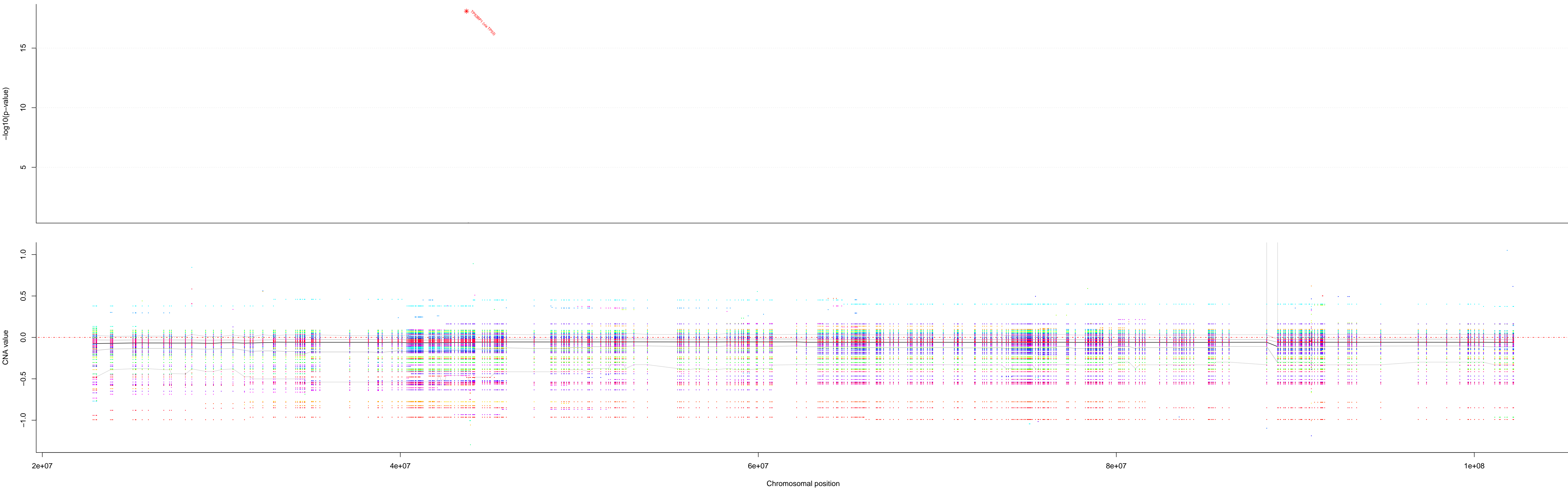

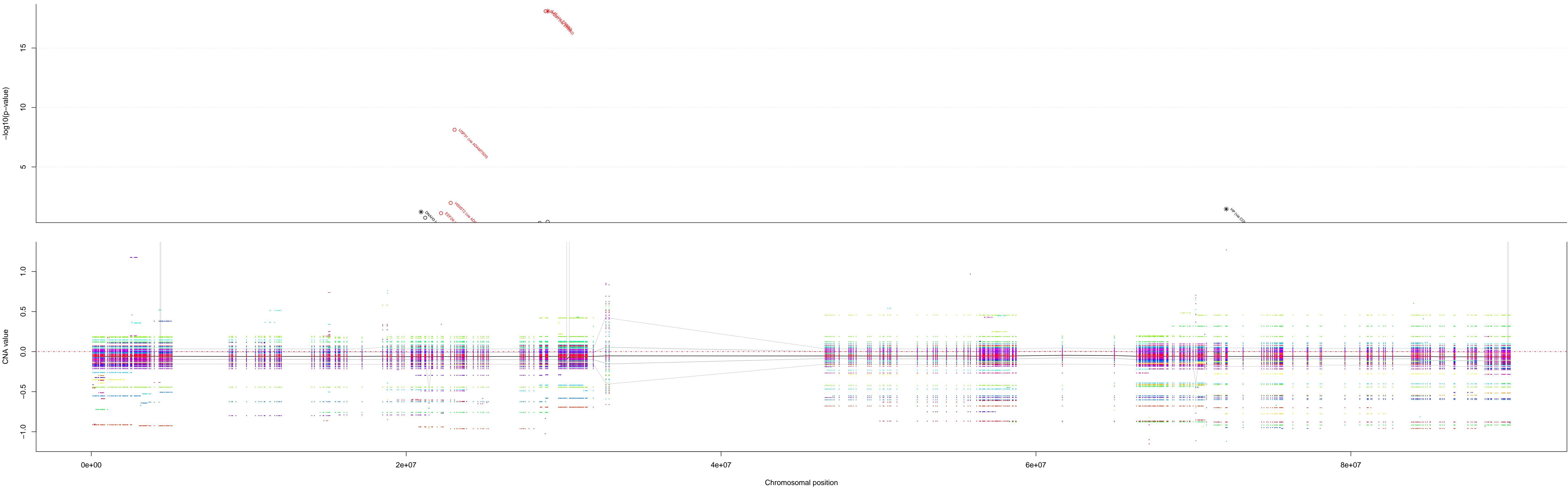

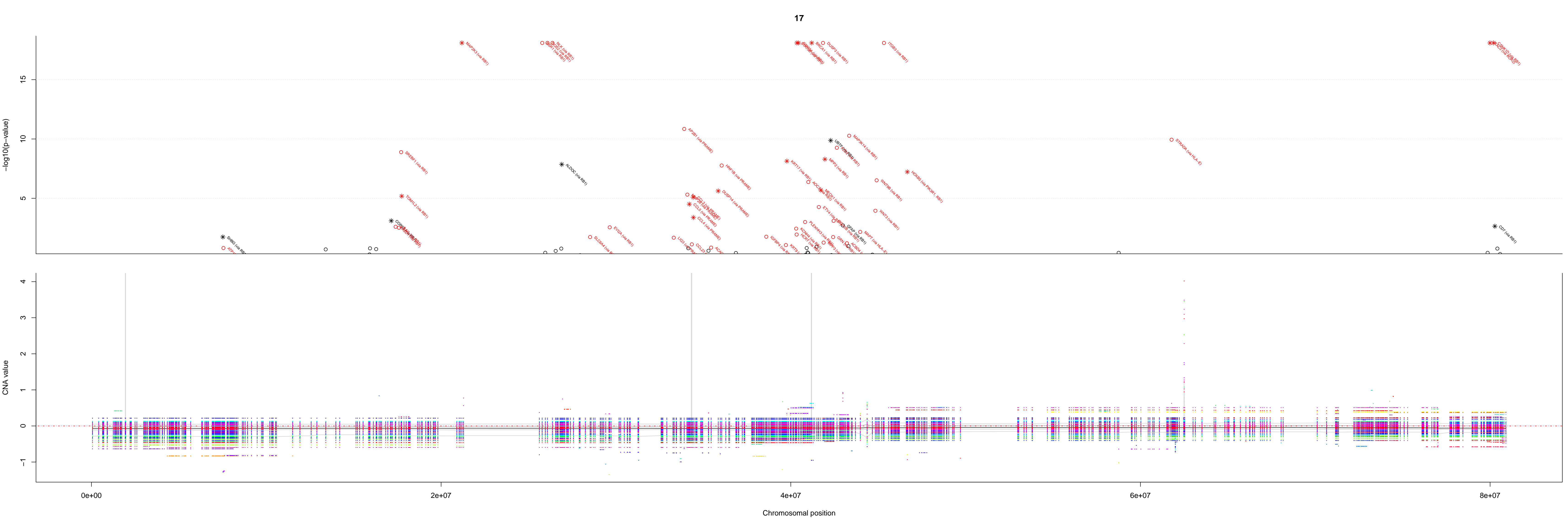

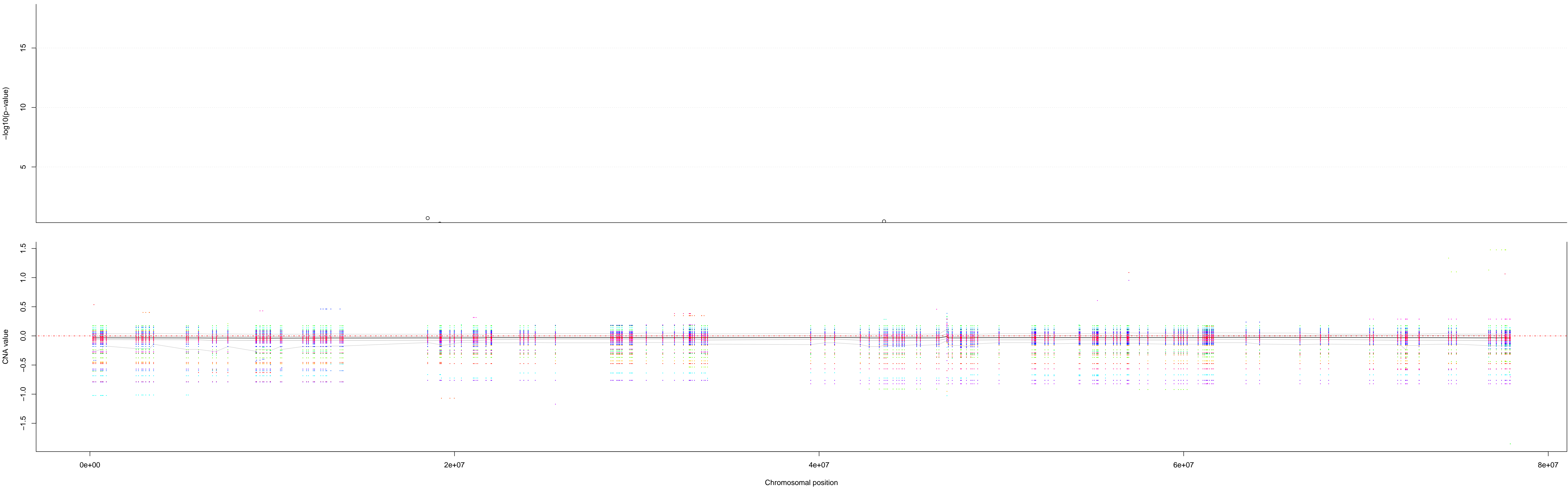

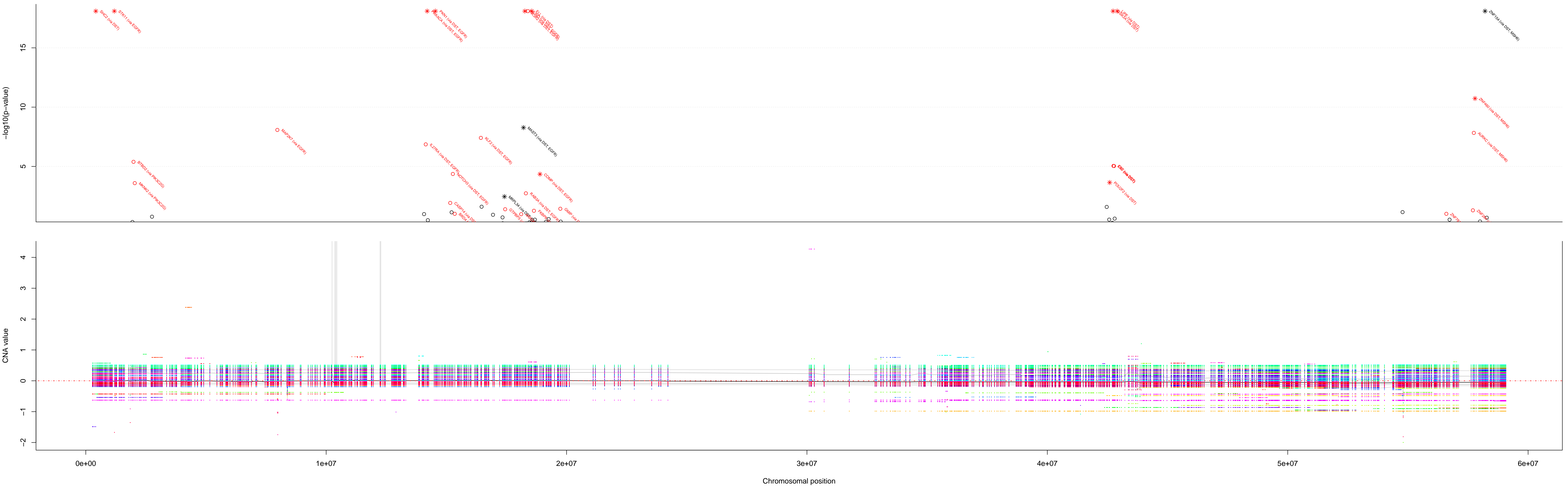

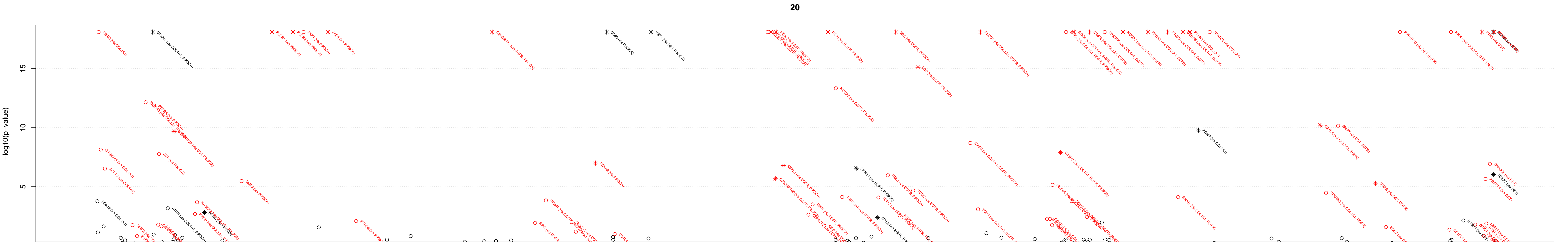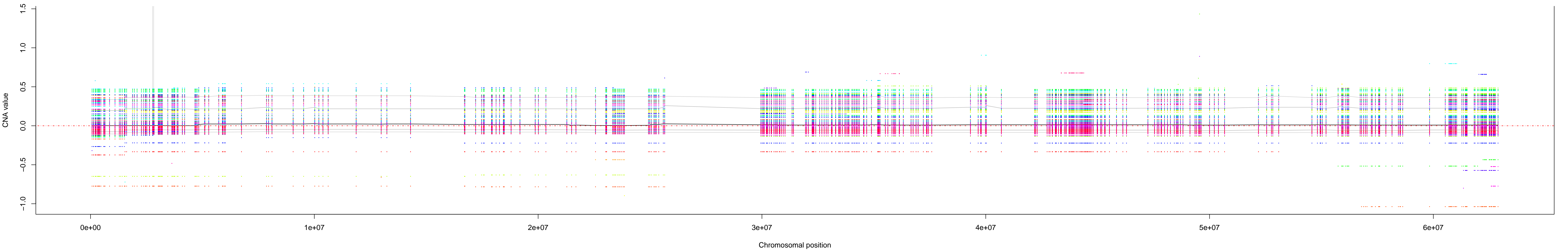

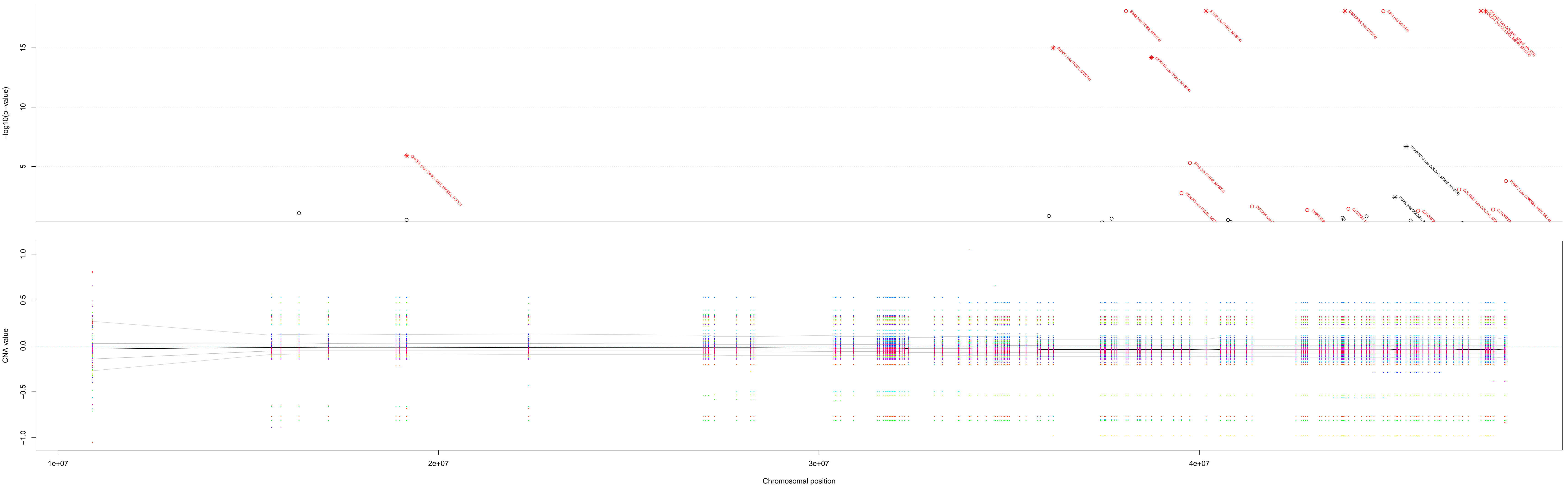

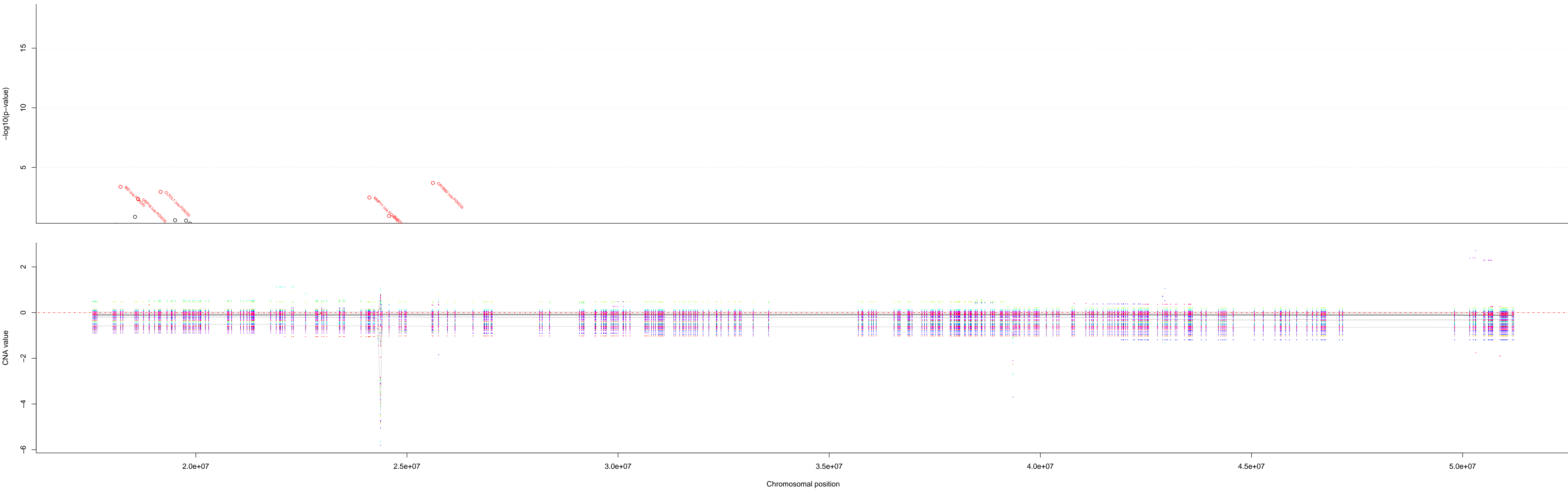

Supplement: Supplementary file 2 — Additional file 2: [GBM.CNA_and_M2CH.alongChromosomes.v7.pdf] contains a graphical representation of copy number driver analysis along the chromosomes in glioblastoma multiforme. (PDF 8 MB) [file 12859_2014_6590_MOESM2_ESM.pdf]

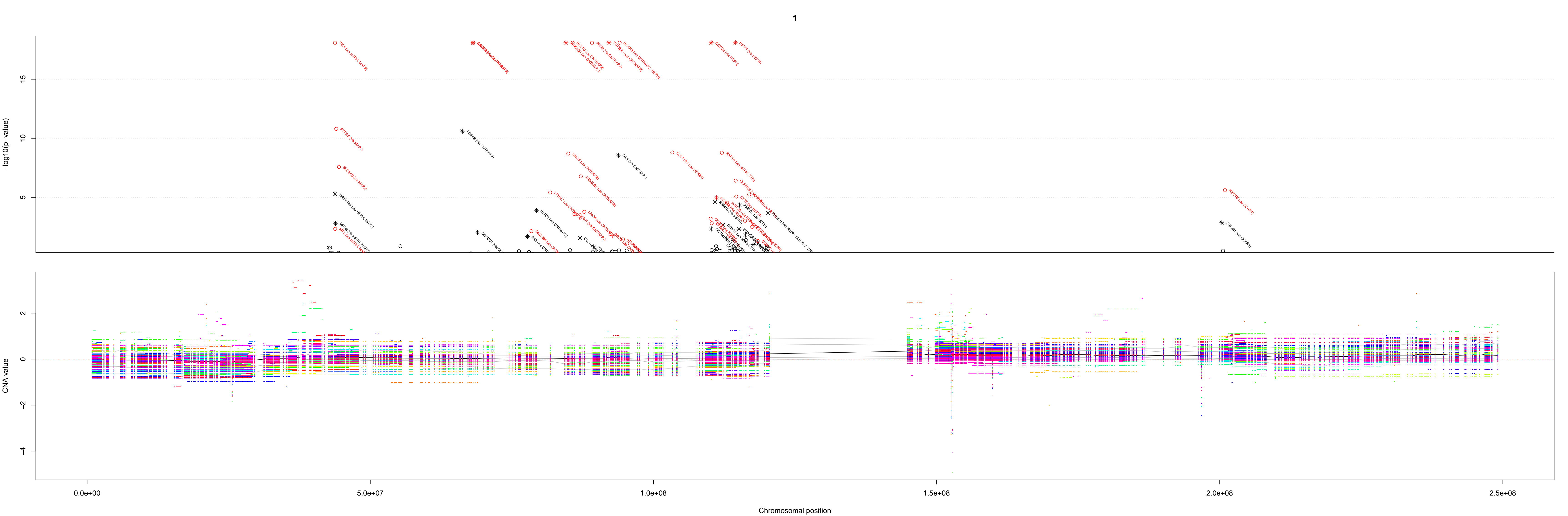

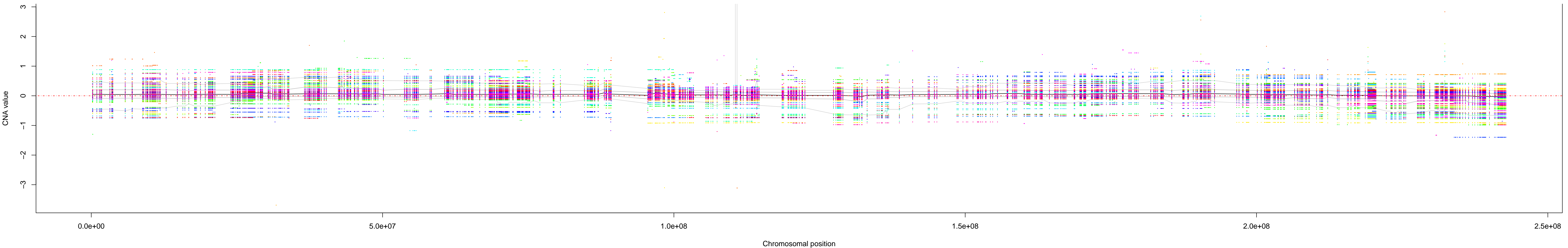

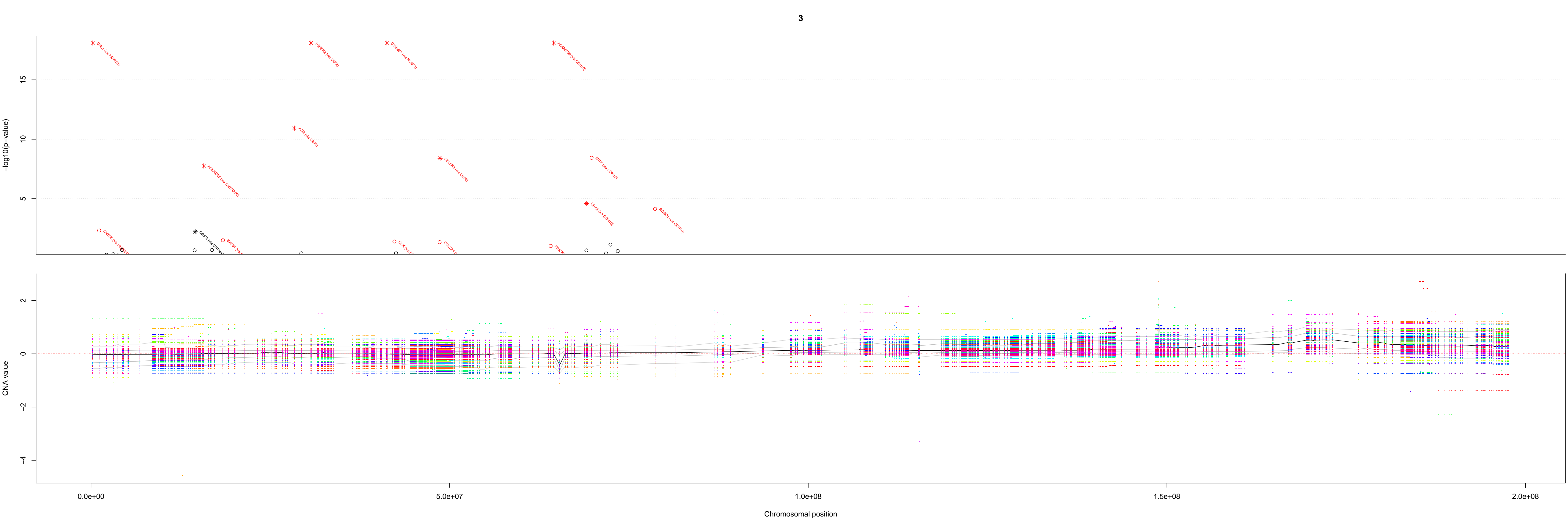

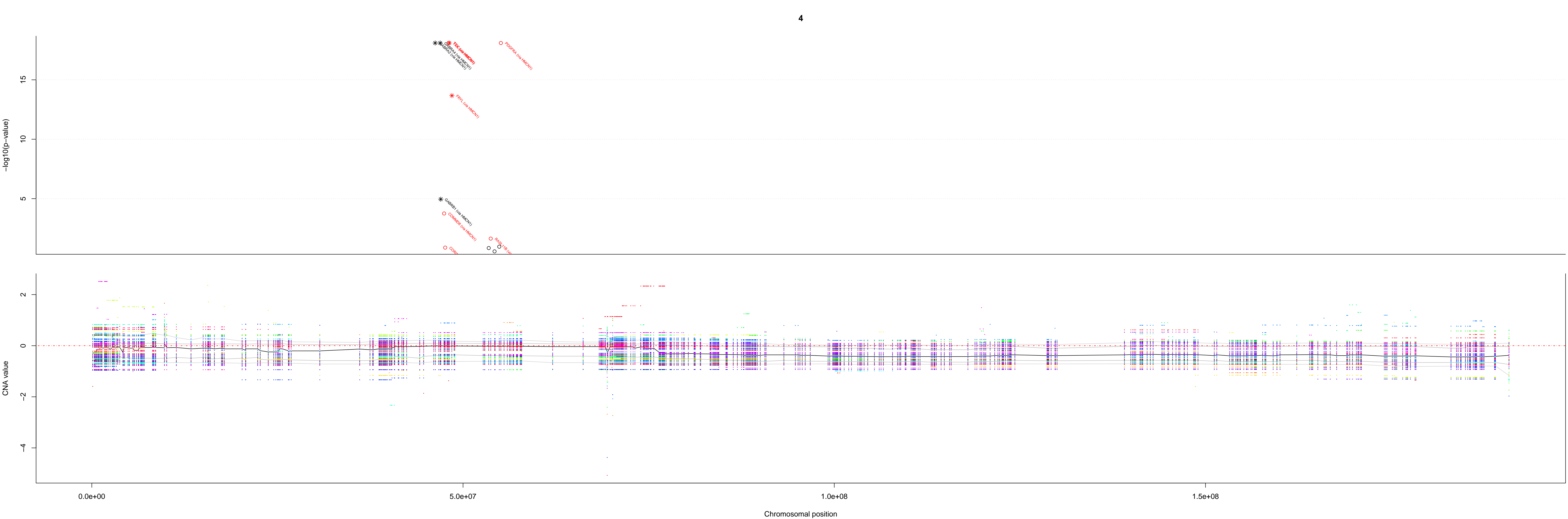

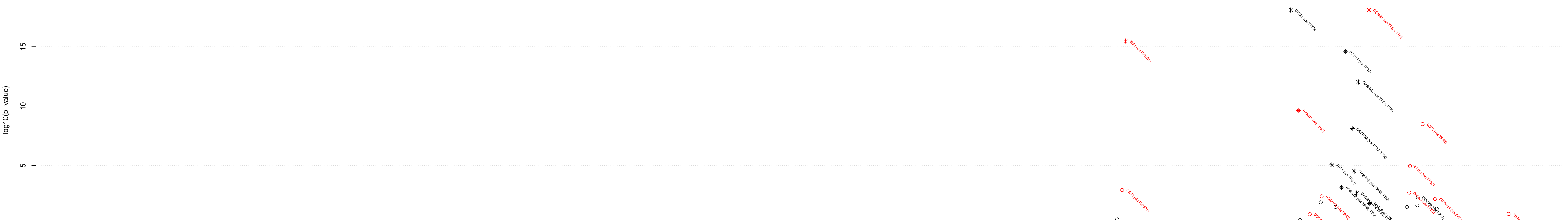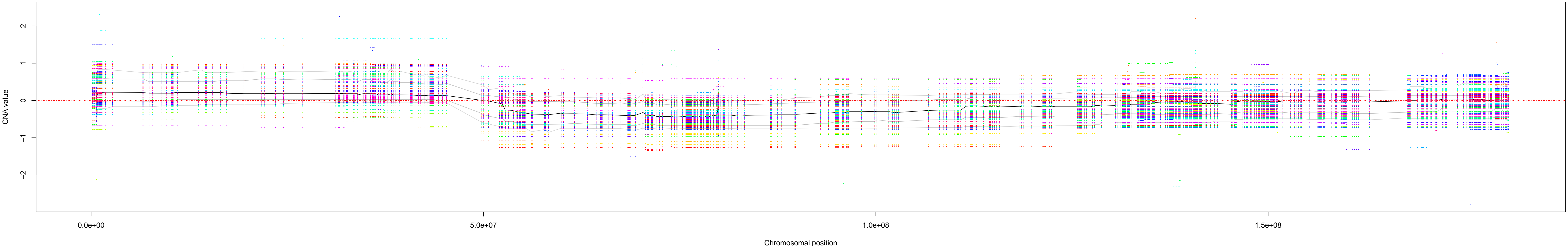

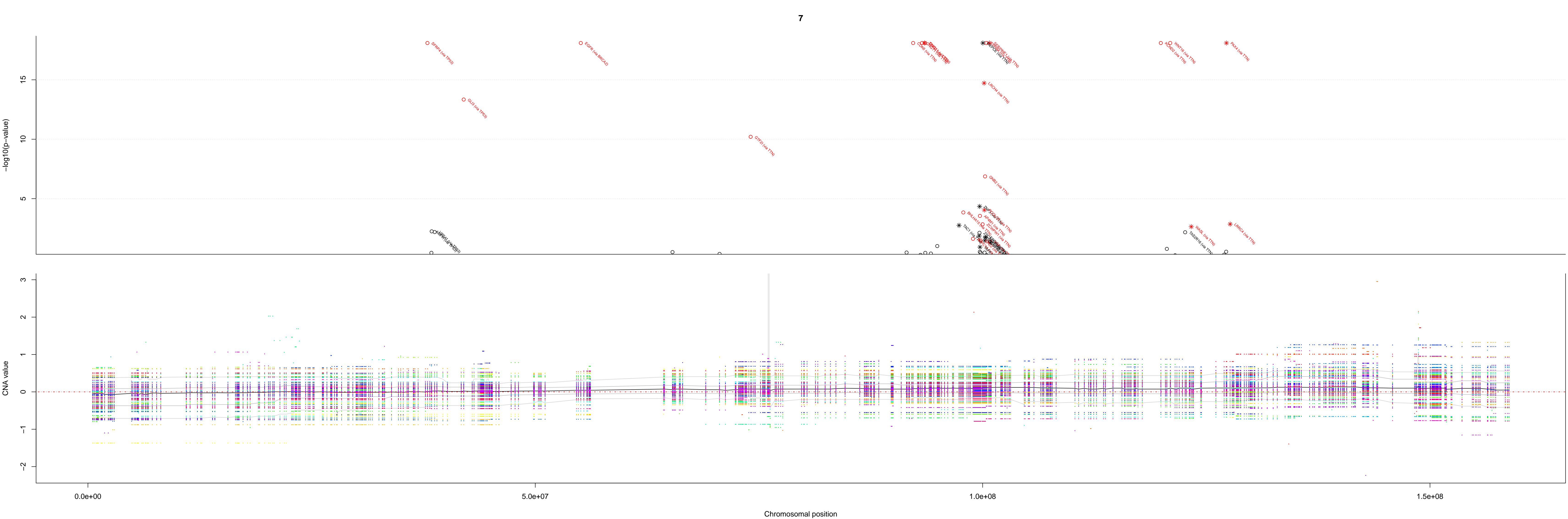

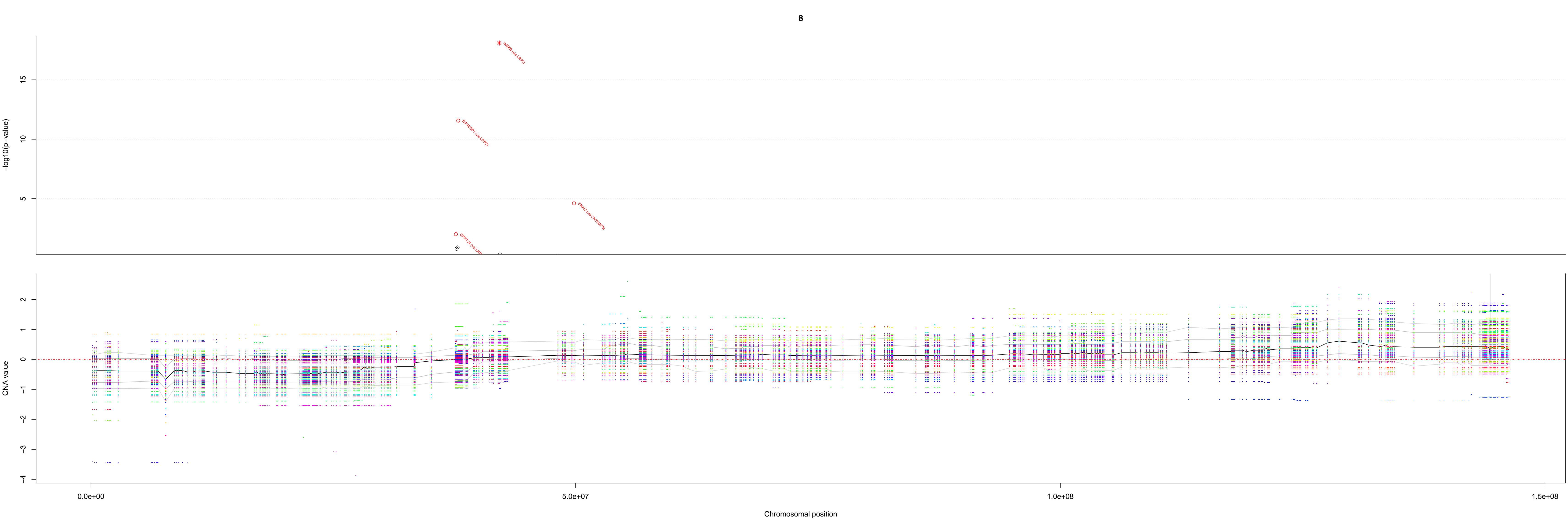

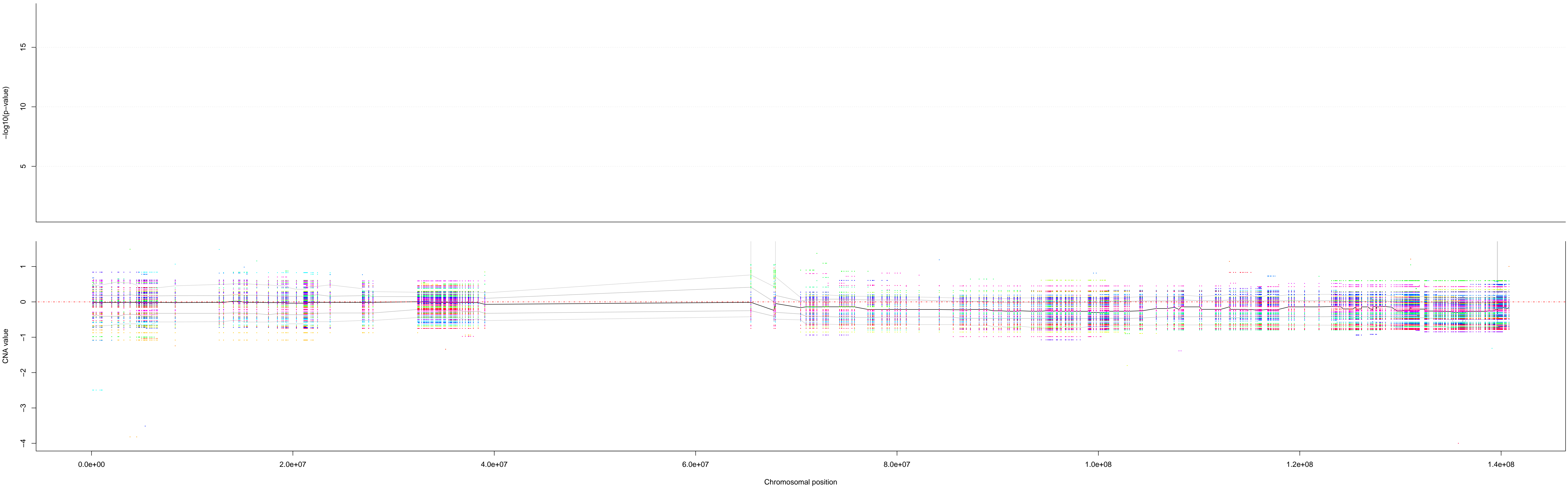

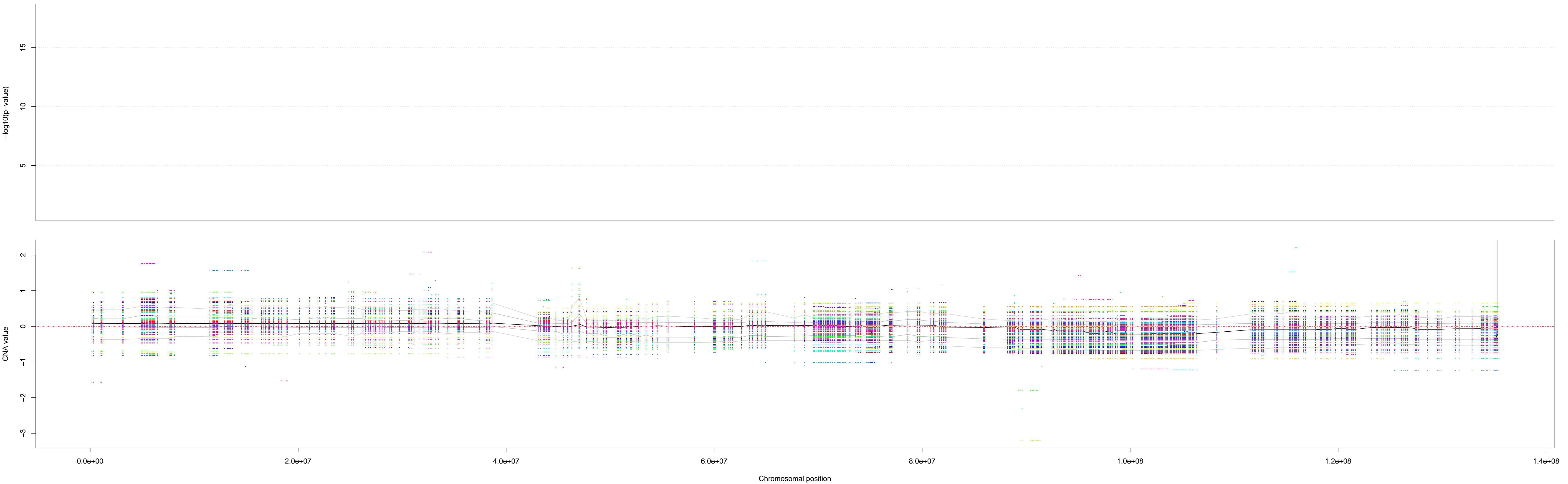

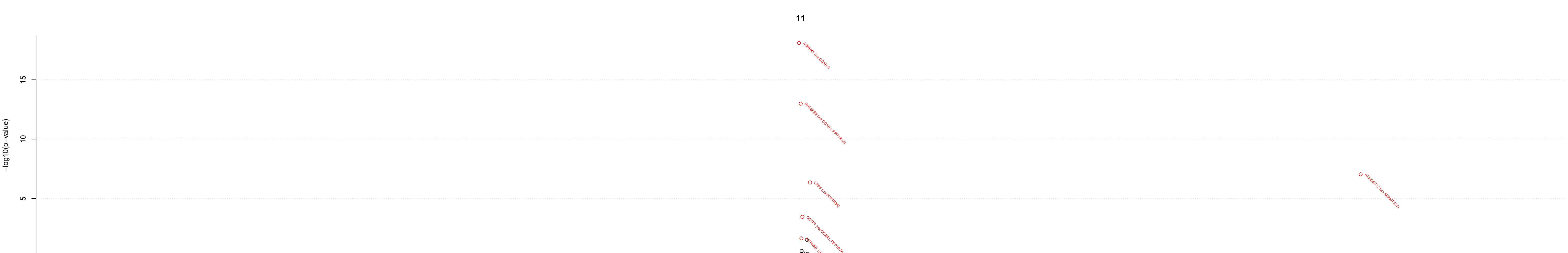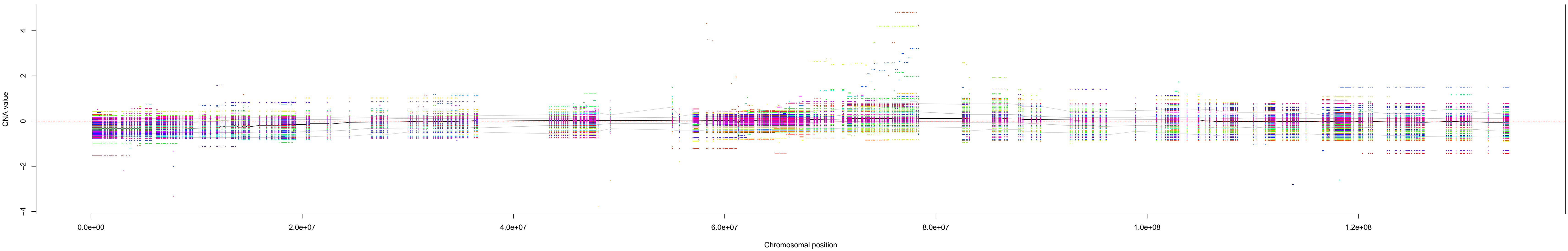

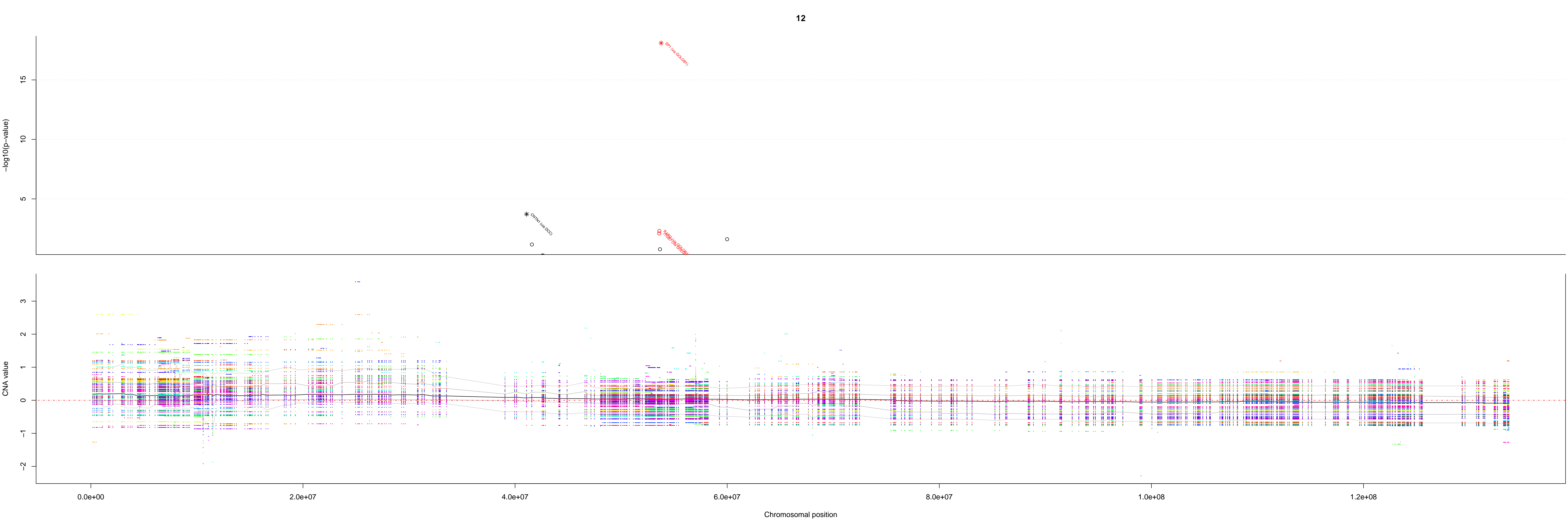

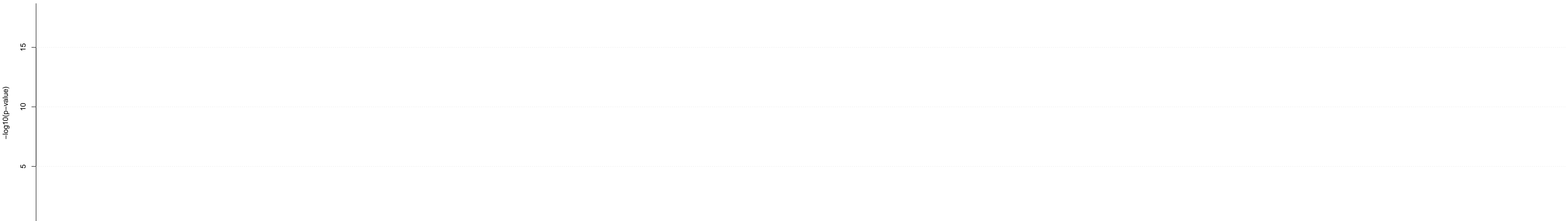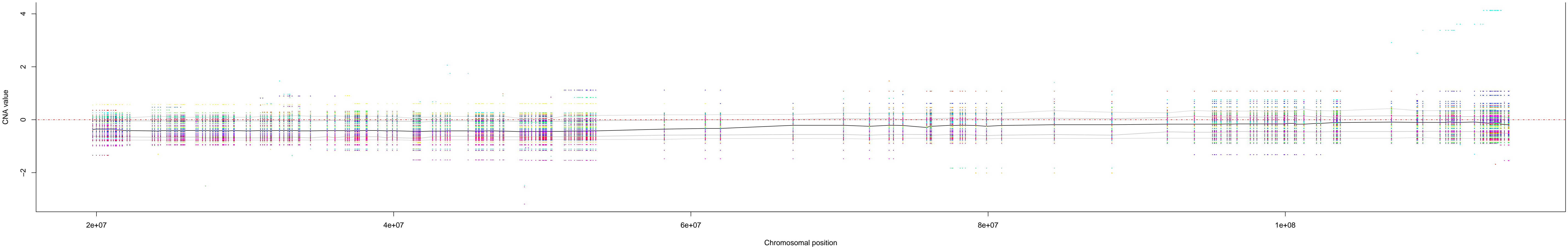

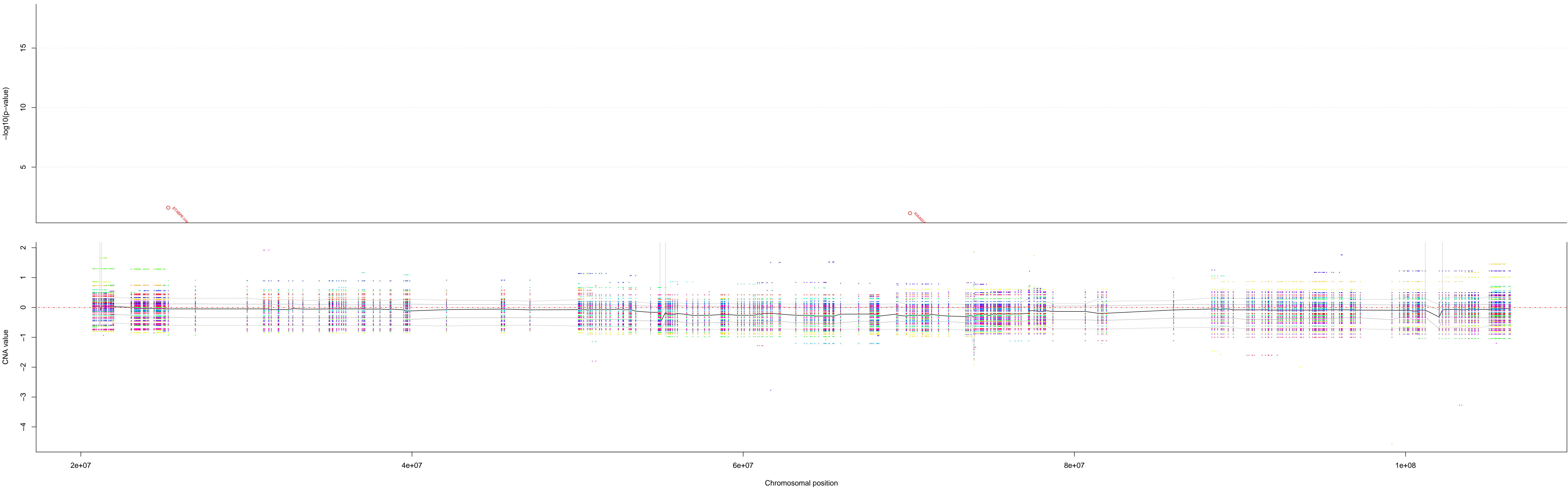

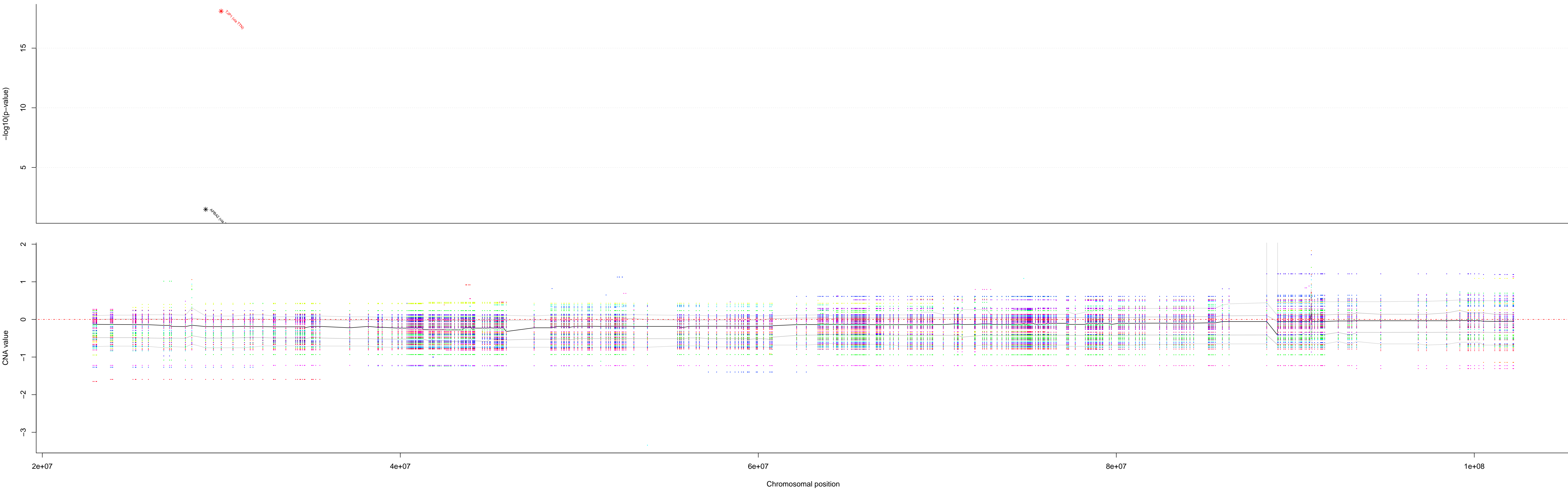

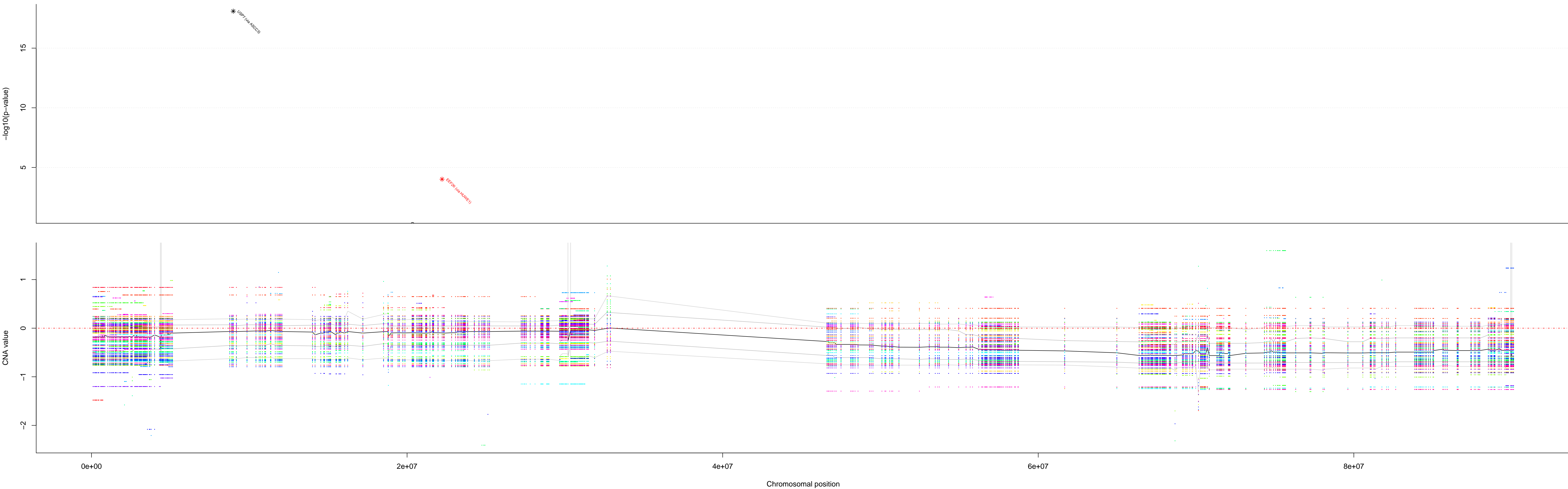

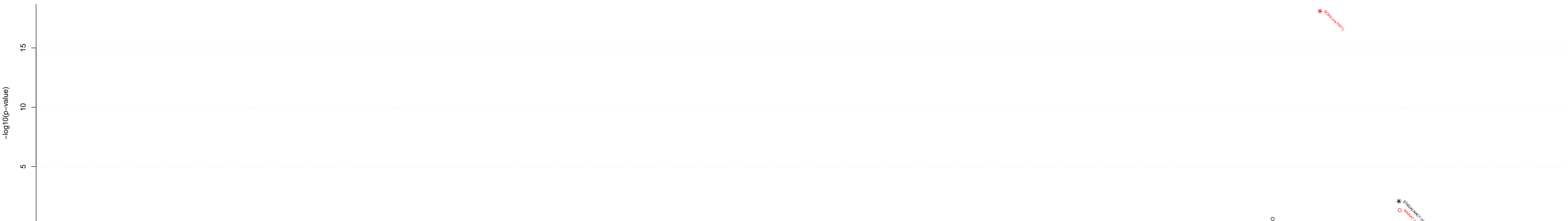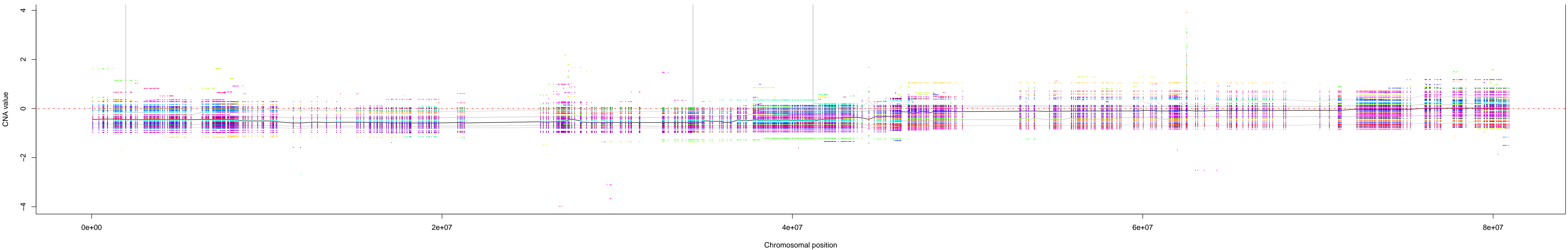

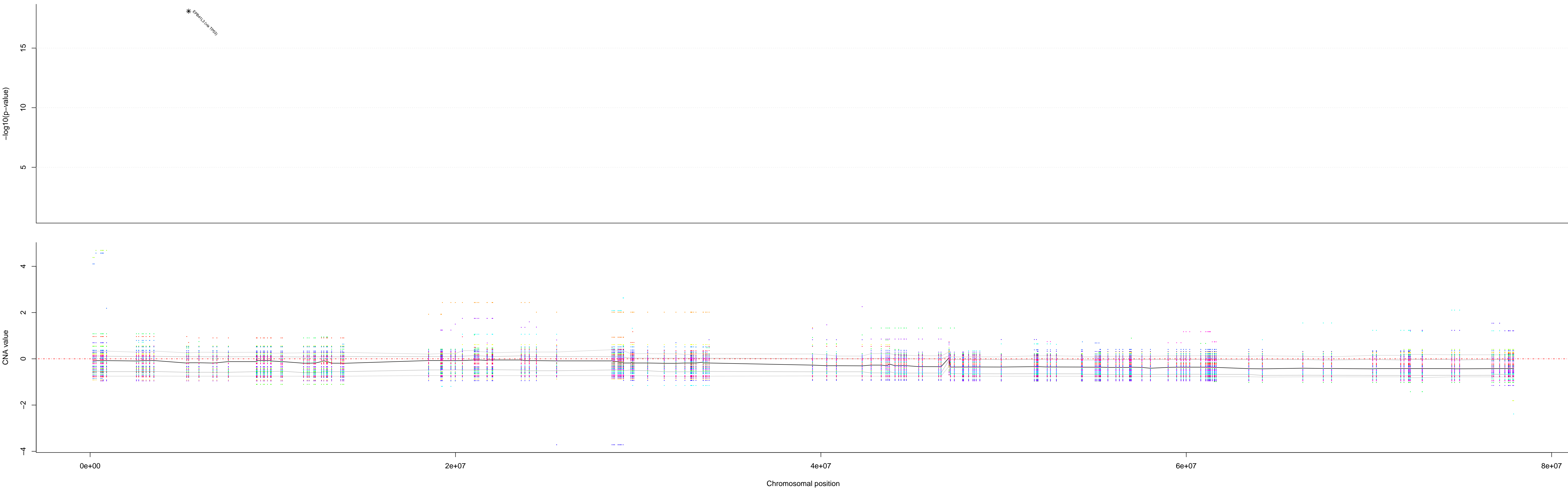

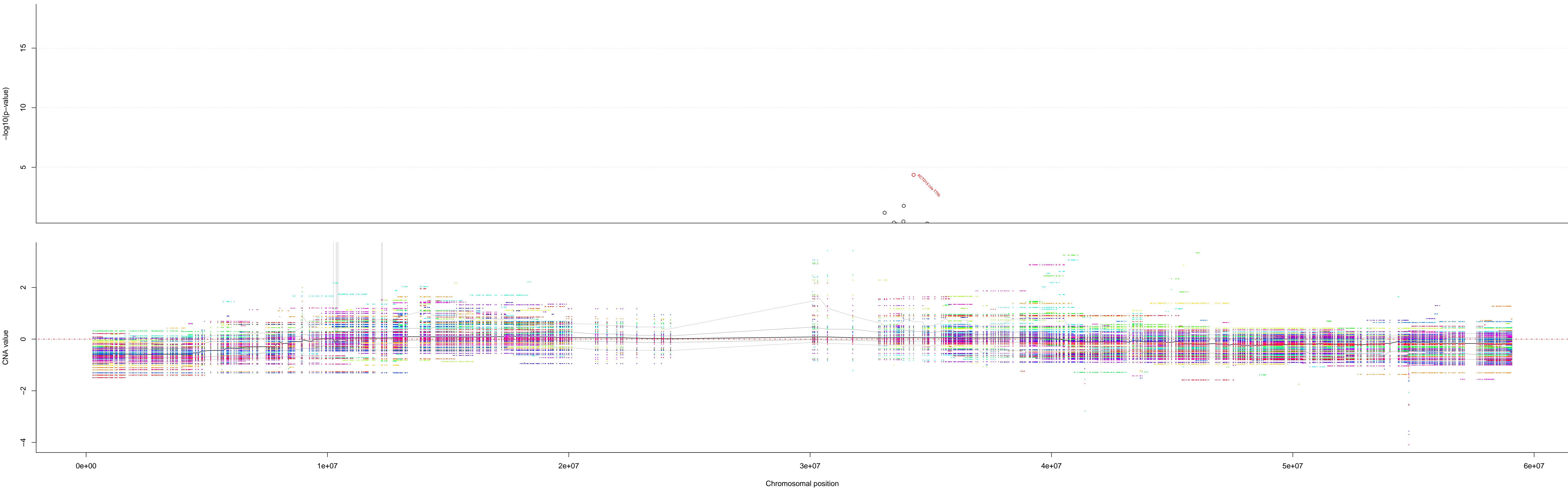

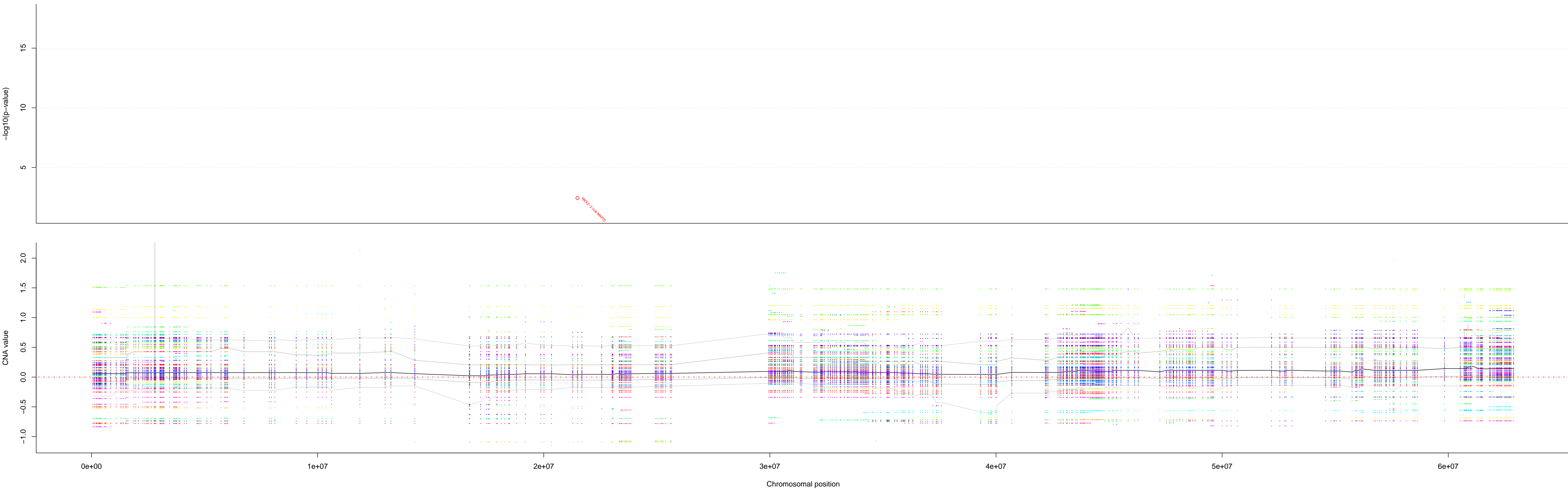

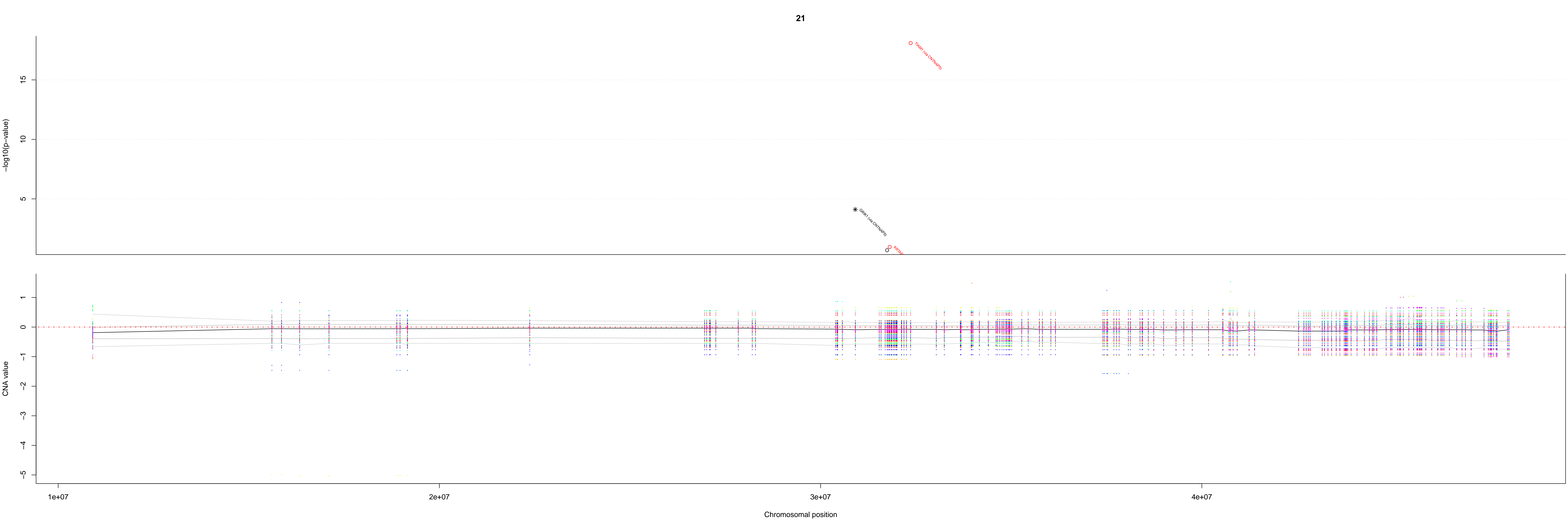

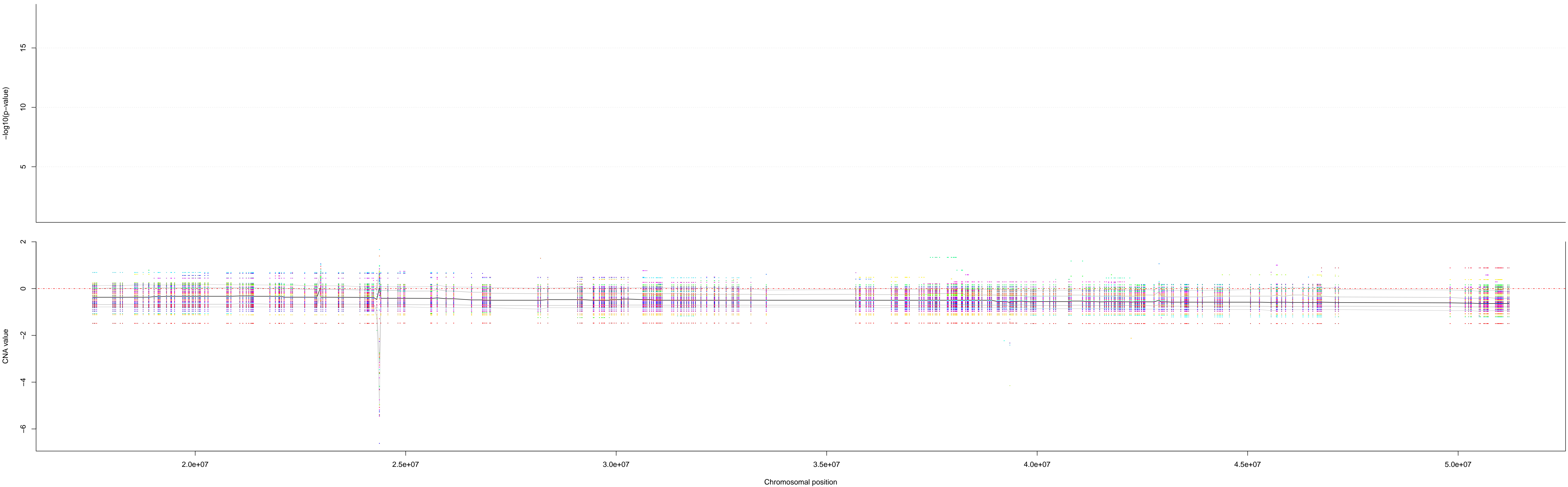

Supplement: Supplementary file 3 — Additional file 3: [OV.CNA_and_M2CH.alongChromosomes.v7.pdf] contains a graphical representation of copy number driver analysis along the chromosomes in ovarian carcinoma. (PDF 5 MB) [file 12859_2014_6590_MOESM3_ESM.pdf]
