# Supplementary material for: Distinguishing between driver and passenger mutations in individual cancer genomes by network enrichment analysis
Source: BMC Bioinformatics. 2014 Sep 19;15(1):308. doi: 10.1186/1471-2105-15-308 (PMC4262241; doi:10.1186/1471-2105-15-308)

## Supplementary methods

### Causal network wir1

In the computational reconstruction of gene networks with high-throughput data, the common approach is to infer network edges by measuring similarity of certain features (usually mRNA expression) across a series of conditions or individuals. The number of highly significant correlations is very large, i.e. far exceeds the density of links expected in a true functional network. The vast majority of correlations in such *correlation networks* are spurious, i.e. explained by activity of a third gene. A number of techniques were proposed to reverse-engineer underlying true networks, such as graphical Gaussian models ( Hartemink et al., 2001), partial mutual information (Frenzel and Pompe, 2004; Margolin et al., 2006), dynamic Bayesian networks (Yu et al., 2004), MNI algorithm (di Bernardo et al., 2005). These methods interpret one data source at a time, thus assuming that all regulation is detectable by this type of data. However for genes profiled in the GBM set, we could calculate a number of alternative similarity metrics, given respective profiles were available. One could discover interconnections between mutated genes with data of three specific types: mRNA expression, methylation, and somatic mutations. Apart from profile pairs of the same type, such as co-expression of two mRNAs, we expected other biologically plausible scenarios, such as:

- mutation in A **->** loss/gain of the ability to methylate B (mut->met),
- methylation of C **->** expression of D (met->exp),
- expression of E **->** expression of F (exp->exp), etc.

These different combinations of data profiles were assumed equally potent in revealing causative relations, given the same effect size. In principle, the underlying regulatory mechanism could remain latent, but we hoped to detect its activity via analysis of respective correlations. To account for this feature, the network inference procedure should be modified.

In the framework of partial correlation analysis, correlation metrics employed for different data types had to be comparable to each other. The *effect size* could serve this purpose. It can be expressed as the coefficient of determination, or *variance component* in gene-gene relation i(x)**->**j(y), i.e. size of the effect of feature *x* of gene *i* on feature *b* of gene *j*  is quantified as the proportion of variance of j(y) due to variation in i(x). As the square of PLC numerically equals the effect size (Steel and Torrie, 1960), it was used to measure relations met**-**met, exp**-**met, met**-**exp, and exp**-**met. On the contrary, mutation profiles were qualitative, binary data. The mutational component of variance *VC_M_*, i.e. effect of mutations in gene *i* on the variability of feature *b* in gene *j* in *N_j_* individuals was calculated as

$VC_{M}=\frac{\sigma_{M}^{2}}{\sigma_{M}^{2}+\sigma_{e}^{2}}$,

where the factorial and residual variances are learned via mean squares *mS* in standard one-way ANOVA:

$\sigma_{M}^{2}=\frac{mS_{M}-mS_{e}}{\frac{N{}_{j}}{2}}$ and $\sigma_{e}^{2}=mS_{e}$

Hence, *VC_M_* was used to measure effect size in cases mut**->**exp and mut**->**met.

Out of nine possible metrics, three (mut**->**mut, met**->**mut, and exp**->**mut) did not have biological sense and were not analyzed.

All available combinations of similarity metrics were processed, and gene pairs with significant ones saved on disk (30,078,185 pairs at *p_α_*<0.01, Table 1). Next, redundant correlations in this primary network had to be resolved to reverse-engineer the most likely causative network. To this end, we performed the partial correlation analysis (PCA). For a pair of genes, their partial correlation indicates the strength of this specific relation *not explainable* by influence of other gene(s). Hence, every network link between genes with a stronger partial correlation was deemed causative, and remained in the resulting *causative* *network*. The details of PCA were explained by Reverter and Chan (2008), who also introduced a flexible information theoretic cutoff for canceling out spurious correlations. We employed this method with the modification for multiple correlations analyzed in parallel. Thus, the squares of effect sizes were input for the PCA. In case of a single effect size was not explained by activity of any third gene, it was accepted as evidence of a causal link (upper values in Table 1). The method of Reverter and Chan (2008) was developed for a single data type. To account for alternative molecular modes of action, we extended the method so that it considered effects from the different data type combinations in parallel. The resulting network wir1 included 48763 links between 12401 genes. Hence, just one in 1308 original correlations (0.07%) proved to indicate a causative relation. Employing three data types rather than one was advantageous: with mRNA expression only, the resulting network would have been smaller by approximately 10%. In the resulting causative network, the impacts of metrics “met–met”, “mut–exp”, and “mut–met” were much higher (0.6%, 2.8%, and 1.4%, respectively) than those of “exp–exp” and “met–exp”. The network wir1 is available from the authors by request.

- [di Bernardo D](http://www.ncbi.nlm.nih.gov/entrez/query.fcgi?db=pubmed&cmd=Search&itool=pubmed_Abstract&term=%22di+Bernardo+D%22%5BAuthor%5D), [Thompson MJ](http://www.ncbi.nlm.nih.gov/entrez/query.fcgi?db=pubmed&cmd=Search&itool=pubmed_Abstract&term=%22Thompson+MJ%22%5BAuthor%5D), [Gardner TS](http://www.ncbi.nlm.nih.gov/entrez/query.fcgi?db=pubmed&cmd=Search&itool=pubmed_Abstract&term=%22Gardner+TS%22%5BAuthor%5D), [Chobot SE](http://www.ncbi.nlm.nih.gov/entrez/query.fcgi?db=pubmed&cmd=Search&itool=pubmed_Abstract&term=%22Chobot+SE%22%5BAuthor%5D), [Eastwood EL](http://www.ncbi.nlm.nih.gov/entrez/query.fcgi?db=pubmed&cmd=Search&itool=pubmed_Abstract&term=%22Eastwood+EL%22%5BAuthor%5D), [Wojtovich AP](http://www.ncbi.nlm.nih.gov/entrez/query.fcgi?db=pubmed&cmd=Search&itool=pubmed_Abstract&term=%22Wojtovich+AP%22%5BAuthor%5D), [Elliott SJ](http://www.ncbi.nlm.nih.gov/entrez/query.fcgi?db=pubmed&cmd=Search&itool=pubmed_Abstract&term=%22Elliott+SJ%22%5BAuthor%5D), [Schaus SE](http://www.ncbi.nlm.nih.gov/entrez/query.fcgi?db=pubmed&cmd=Search&itool=pubmed_Abstract&term=%22Schaus+SE%22%5BAuthor%5D), [Collins JJ](http://www.ncbi.nlm.nih.gov/entrez/query.fcgi?db=pubmed&cmd=Search&itool=pubmed_Abstract&term=%22Collins+JJ%22%5BAuthor%5D) (2005) Chemogenomic profiling on a genome-wide scale using reverse-engineered gene networks. Nat. Biotechnol. 23: 377-383
- Frenzel S &, Pompe B. Partial mutual information for coupling analysis of multivariate time series. Phys Rev Lett. 2007 Nov 16;99(20):204101.
- Hartemink, A., Gifford, D., Jaakkola, T., & Young, R. Using graphical models and genomic expression data to statistically validate models of genetic regulatory networks. In Pacific Symposium on Biocomputing 2001 (PSB01) Altman, R., Dunker, A.K., Hunter, L., Lauderdale, K., & Klein, T., eds. World Scientific: New Jersey. pp. 422–433.
- Margolin AA, Nemenman I, Basso K, Wiggins C, Stolovitzky G, Dalla Favera R, Califano A.ARACNE: an algorithm for the reconstruction of gene regulatory networks in a mammalian cellular context. BMC Bioinformatics. 2006 Mar 20;7 Suppl 1:S7.
- Reverter A, Chan EK. Combining partial correlation and an information theory approach to the reversed engineering of gene co-expression networks. Bioinformatics. 2008 Nov 1;24(21):2491-7.
- Yu, J., Smith, V., Wang, P., Hartemink, A., & Jarvis, E. Advances to Bayesian network inference for generating causal networks from observational biological data. Bioinformatics, 20, December 2004. pp. 3594–3603

Table S1. List and features of benchmarked networks.

| **Network ID** | **No. of nodes** | **No. of edges** | **Description** | **Overlap with KEGG edges** | **Added Phosphosite, KEGG, CORUM edges not present and added**  **(out of total 77260)** |
| --- | --- | --- | --- | --- | --- |
| FC2_full | 19357 | 4601749 | Latest (2.0) release of FunCoup* based on data from human and 9 other organisms, full version; edge confidence cutoff FBS>4.71 | 18749 | NA |
| FClim | 15767 | 1391225 | Special version produced by FunCoup, with limited data from model organisms (mouse, rat, D.melanogaster, C.elegans, S.cerevisiae) ; edge confidence cutoff FBS>4.00 | 13862 | NA |
| FC1_full | 15882 | 2024752 | Older release of FunCoup based on data from human and 7 other organisms; edge confidence cutoff FBS>3.00 | 11508 | NA |
| STRING9_full | 18021 | 1630508 | Latest (9.0) release of STRING** | 33990 | NA |
| FC2_ref | 12638 | 911327 | Same as FC2_full but edge confidence cutoff FBS>9.38, so that the number of edges equals that in FClim_ref | 12924 | NA |
| FC1_ref | 14490 | 911327 | Same as FC1_full but edge confidence cutoff FBS>4.17, so that the number of edges equals that in FClim_ref | 9292 | NA |
| FClim_ref | 14586 | 911327 | Same as FC_lim but edge confidence FBS>4.71 which equals the minimum cutoff in FC2_full | 12384 | NA |
| STRING9_ref | 17501 | 911327 | Same as STRING9_full; edge confidence cutoff combined_score>255, so that the number of edges equals that in FClim_ref | 32818 | NA |
| FC2_highconf | 10909 | 450000 | Same as FC2_full; with edge confidence cutoff FBS> 12.10, so that all PhosphoSite, KEGG, CORUM edges are included regardless of presence in this network | NA | 39721 |
| FClim_ highconf | 13358 | 450000 | Same as FC_lim; edge confidence cutoff FBS> 6.13, so that all PhosphoSite, KEGG, CORUM edges are included regardless of presence in this network | NA | 47729 |
| FC1_highconf | 13969 | 450000 | Same as FC1_full; with edge confidence cutoff FBS> 5.71, so that all PhosphoSite, KEGG, CORUM edges are included regardless of presence in this network | NA | 54402 |
| STRING9_ highconf | 16839 | 450000 | Same as STRING; edge confidence cutoff combined_score>475, so that all PhosphoSite, KEGG, CORUM edges are included regardless of presence in this network | NA | 29843 |
| FC2_HC | 14124 | 940000 | FC2_ highconf with edge confidence cutoff relaxed further, so that 490000 more edges from FC2_ref were added | NA | NA |
| FClim_ HC | 15243 | 940000 | FClim _ highconf with edge confidence cutoff relaxed further, so that 490000 more edges from FClim _ref were added | NA | NA |
| FC1_ HC | 15500 | 940000 | FC1_ highconf with edge confidence cutoff relaxed further, so that 490000 more edges from FC1_ref were added | NA | NA |
| STRING9_ HC | 17594 | 940000 | STRING9_ highconf with edge confidence cutoff relaxed further, so that 490000 more edges from STRING9_ref were added | NA | NA |
| Wir1 | 12401 | 48763 | Causative network from TCGA glioblastoma expression, methylation, and mutation data sets | 82 | NA |
| Wir.OV.0.5 | 5851 | 6441 | Causative network from TCGA ovarian cancer expression set | 70 | NA |
| FClim_PPI | 9494 | 156978 | Sub-network of FC_lim where each edge should have had support from protein-protein interactions with confidence FBS>4 | NA | NA |
| Primary.OV_150 | 7465 | 150000 | Relevance network: edges are gene pairs prioritized by Pearson linear correlation in GBM (r>0.422) | NA | NA |
| Primary.GBM_150 | 6759 | 150000 | Relevance network: edges are gene pairs prioritized by Pearson linear correlation in OV (r>0.654) | NA | NA |
| FClim_PPI_and_ Primary.OV_150 | 16959 | 306376 | Merge of FClim_PPI and Primary.OV_150 | NA | NA |
| FClim_PPI_and_ Primary.GBM_150 | 16253 | 306368 | Merge of FClim_PPI and Primary.GBM_150 | NA | NA |
| iRefIndex | 12566 | 382230 | Non-redundant HUGO ID pairs from the union of protein-protein interactions available at iRefIndex 9.0*** | NA | NA |
| OV_TRANSFAC | 2294 | 5907 | Experiment-based: regulatory interactions in TRANSFAC mapped to ovarian cancer data sets**** | NA | NA |
| merged6_and_wir1_HC2 | 19028 | 974427 | FClim_highconf which, in addition to all edges from PhosphoSite, KEGG, CORUM, contained also TF-target links from MSigDB and all edges from wir1 | NA | NA |

* Alexeyenko A, Sonnhammer EL: Global networks of functional coupling in eukaryotes from comprehensive data integration. Genome Res 2009, Jun;19(6):1107-16.

** von Mering C, Jensen LJ, Snel B, Hooper SD, Krupp M, Foglierini M, Jouffre N, Huynen MA, Bork P: STRING: Known and predicted protein–protein associations, integrated and transferred across organisms. Nucleic Acids Res 2005, 33:D433–D437.

*** Razick S, Magklaras G, Donaldson IM: iRefIndex: a consolidated protein interaction database with provenance. BMC Bioinformatics 2008, Sep 30;9:405.

****[di Bernardo D](http://www.ncbi.nlm.nih.gov/entrez/query.fcgi?db=pubmed&cmd=Search&itool=pubmed_Abstract&term=%22di+Bernardo+D%22%5BAuthor%5D), [Thompson MJ](http://www.ncbi.nlm.nih.gov/entrez/query.fcgi?db=pubmed&cmd=Search&itool=pubmed_Abstract&term=%22Thompson+MJ%22%5BAuthor%5D), [Gardner TS](http://www.ncbi.nlm.nih.gov/entrez/query.fcgi?db=pubmed&cmd=Search&itool=pubmed_Abstract&term=%22Gardner+TS%22%5BAuthor%5D), [Chobot SE](http://www.ncbi.nlm.nih.gov/entrez/query.fcgi?db=pubmed&cmd=Search&itool=pubmed_Abstract&term=%22Chobot+SE%22%5BAuthor%5D), [Eastwood EL](http://www.ncbi.nlm.nih.gov/entrez/query.fcgi?db=pubmed&cmd=Search&itool=pubmed_Abstract&term=%22Eastwood+EL%22%5BAuthor%5D), [Wojtovich AP](http://www.ncbi.nlm.nih.gov/entrez/query.fcgi?db=pubmed&cmd=Search&itool=pubmed_Abstract&term=%22Wojtovich+AP%22%5BAuthor%5D), [Elliott SJ](http://www.ncbi.nlm.nih.gov/entrez/query.fcgi?db=pubmed&cmd=Search&itool=pubmed_Abstract&term=%22Elliott+SJ%22%5BAuthor%5D), [Schaus SE](http://www.ncbi.nlm.nih.gov/entrez/query.fcgi?db=pubmed&cmd=Search&itool=pubmed_Abstract&term=%22Schaus+SE%22%5BAuthor%5D), [Collins JJ](http://www.ncbi.nlm.nih.gov/entrez/query.fcgi?db=pubmed&cmd=Search&itool=pubmed_Abstract&term=%22Collins+JJ%22%5BAuthor%5D): Chemogenomic profiling on a genome-wide scale using reverse-engineered gene networks. Nat Biotechnol 2005, 23: 377-383.

Table S2. Network enrichment analysis (NEA) of genes found in MEMo modules (Ciriello et al., 2011).

| Gene symbol | p-value in NEA modes** | | |  |
| --- | --- | --- | --- | --- |
|  | 1-vs-CPW | 1point-vs-MGS | 1CNA-vs-MGS | p.total.combined |
| GBM | | | | |
| RB1 | 0*** | 0 | 0 | 0 |
| CDK4 | 0 | NA | 0.99 | 2.63e-14 |
| CDKN2A | 0 | 0 | 0 | 0 |
| CDKN2B | 0 | NA | 0.909 | 2.41e-14 |
| TP53 | 0 | 0 | 6.66e-16 | 0 |
| MDM2 | 0 | 0.5 | 0 | 0 |
| MDM4 | 0 | 0.5 | 0.39 | 8.97e-14 |
| PDGFRA | 0 | 0.73 | 0 | 0 |
| PTEN | 0 | 0 | 0 | 0 |
| EGFR | 0 | 0 | 0 | 0 |
| PIK3R1 | 0 | 4.90e-14 | 1.86e-11 | 0 |
| GLI1 | 0 | 0.5 | 0.76 | 1.70e-13 |
| NF1 | 8.78e-07 | 0.0003 | 0.0002 | 2.39e-11 |
| OV | | | | |
| BRCA1 | 0 | 0.37 | 0 | 0 |
| CCNE1 | 0.98 | NA | 0.06 | 0.18 |
| RBBP8 | 7.49e-13 | NA | 3.43e-06 | 8.32e-17 |
| BRCA2 | 0 | 4.99e-15 | 0 | 0 |
| RB1 | 0 | 0.78 | 0 | 0 |
| MYC | 0 | NA | 4.48e-10 | 0 |
| RNF144B | 0.967 | NA | NA | 0.97 |
|  |  |  |  |  |

* All genes found in at least one MEMo module with q-value<0.1 in either version of the network (HRN1 or HRN2) were analyzed separately for GBM and OV datasets.

** since point and CNA alterations of the same gene could occur in multiple GBM and OV samples, their p-values were derived for each sample separately, and then combined with Fisher’s formula. The combined p-values for each gene are shown in columns 1point-vs-MGS and 1CNA-vs-MGS. Next, these latter were combined with the p-values from 1-vs-CPW using the same formula, and the resulting gene-wise value is shown in the column “p.total.combined”;

***P-values below 10^-18 are given as plain zeroes.

Figure S1. Benchmarking alternative global networks.

ROC curves evaluated differential performance of the different network versions in predicting members of KEGG pathways and cancer-related gene sets (see Methods for the benchmark description).


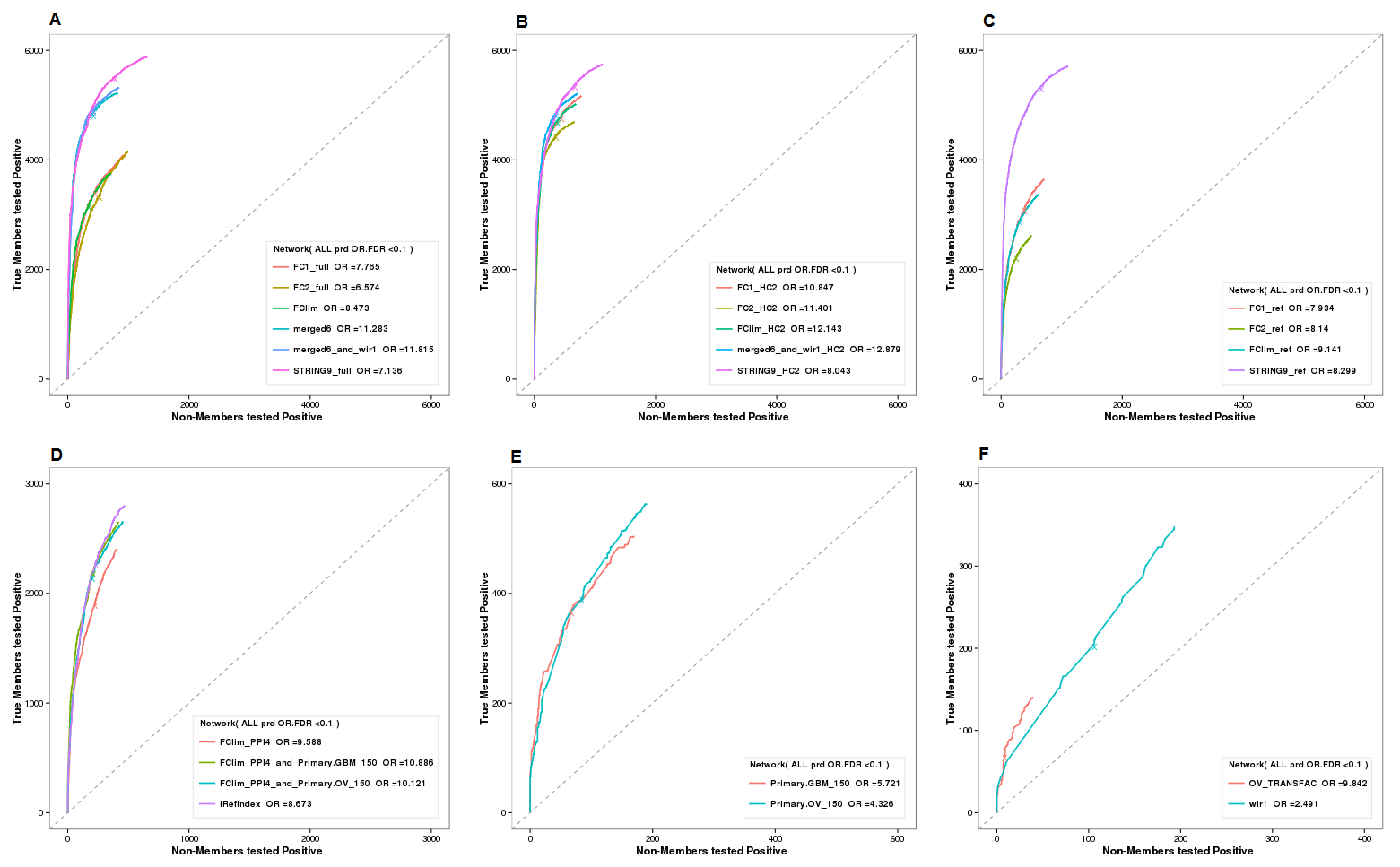


Figure S2. Correspondence of NEA scores received by individual genes in 1-vs-CPW, 1point-vs-MGS, and 1CNA-vs-MGS, summarized over all cancer pathways and all GBM and OV samples.

The visual and correlation analysis demonstrate that driver roles of the same gene can, in many cases, be revealed by different approached. The two plots on the right side (1point-vs-MGS vs.1CNA-vs-MGS) refer to cases when the same gene was either copy number altered or obtained a point mutation in different genomes. Those genes that had NEA Z = 5or higher in both dimensions were detected as drivers by the both methods.


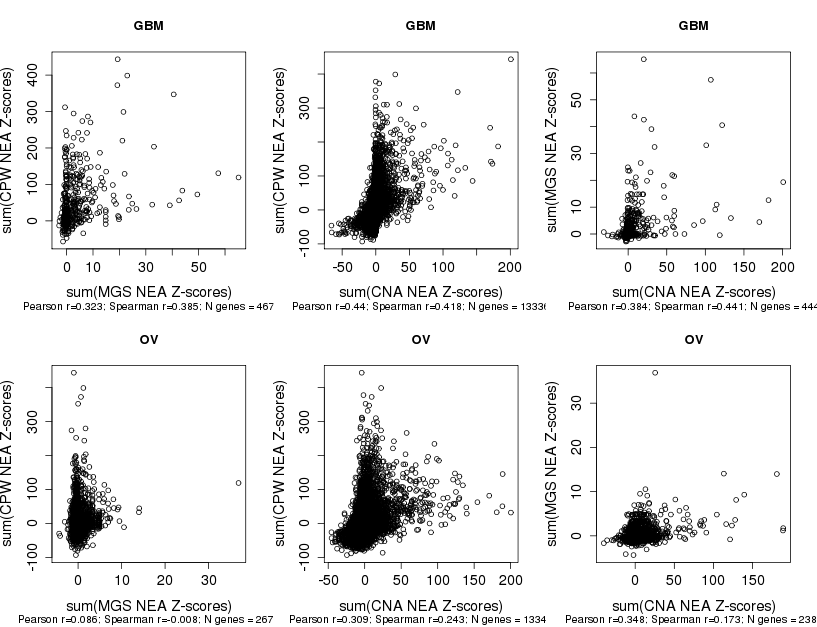


Figure S3. Correlation between copy number and mRNA expression of same gene.

The plot shows that copy number changes in both well known (A) and suggested (B) cancer drivers far from always explicitly alter mRNA expression, compared to the bulk of CNA genes (first rows at A and B).

1. Spearman rank correlations between copy number (*log_2_*-transformed CNA values from HMS HG-CGH-244A arrays) and mRNA expression profiles. Histograms in rows present gene subsets from GBM and OV sets: 1) all CNA genes (no filtration), 2) the list of mut-drivers (Vogelstein et al., 2013), 3) cancer predisposition genes (*ibid.*, Table S4) 4) CAN-genes by Parsons et al. (2008, Table S7).
2. Same as in A, but the histograms in rows 2-6 present gene subsets that passed different levels of our analysis: 2) co-occurred with any point mutations, 3) co-occurred with multiple point mutations (p.mm.combined), 4) received high NEA scores for relations to known cancer pathways (hence low p-value p.cpw), 5) received high NEA scores for relations to point mutations in the same genome (low p.nea.combined) and 6) scored high in the integration of tests 3, 4, and 5 above (low p.total).
3. Expression and CNA values of nine most likely CNA drivers in GBM (by criteria 3-5 listed in B).
4. Expression and CNA values of nine most likely CNA drivers in OV (by criteria 3-5 listed in B).

A


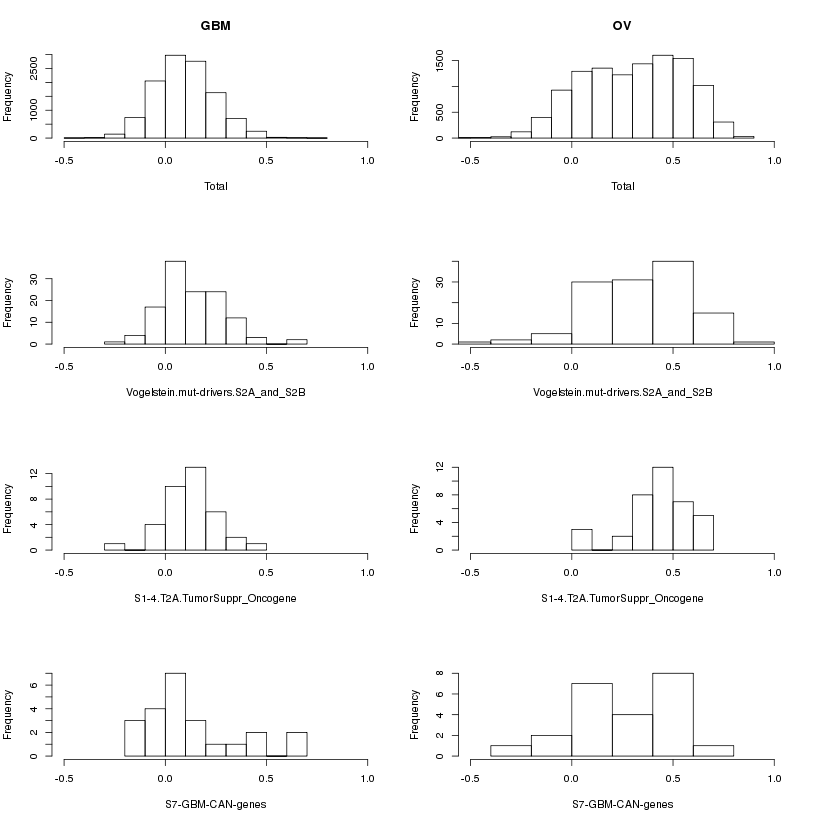


B


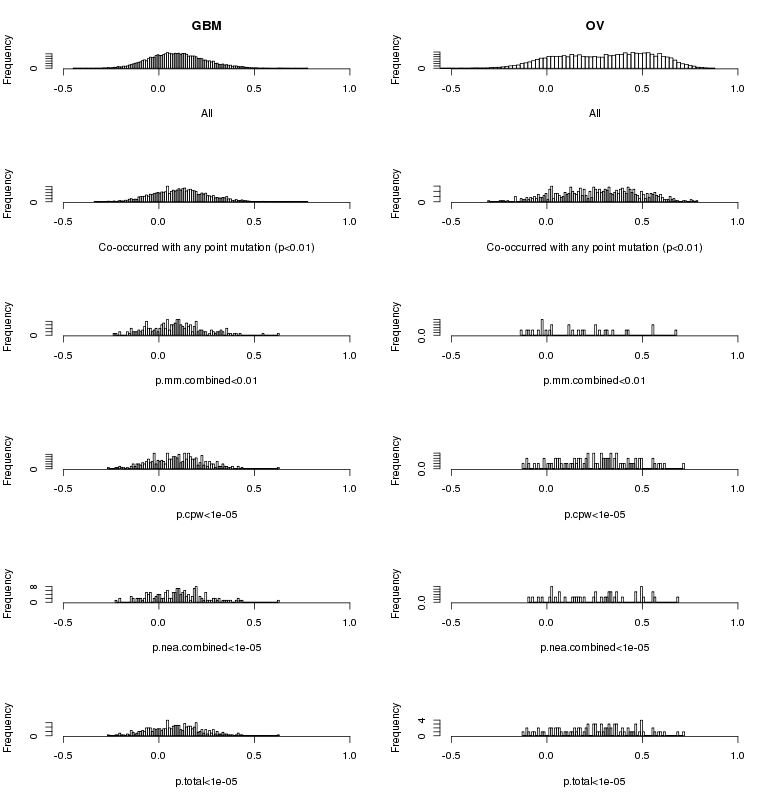


C


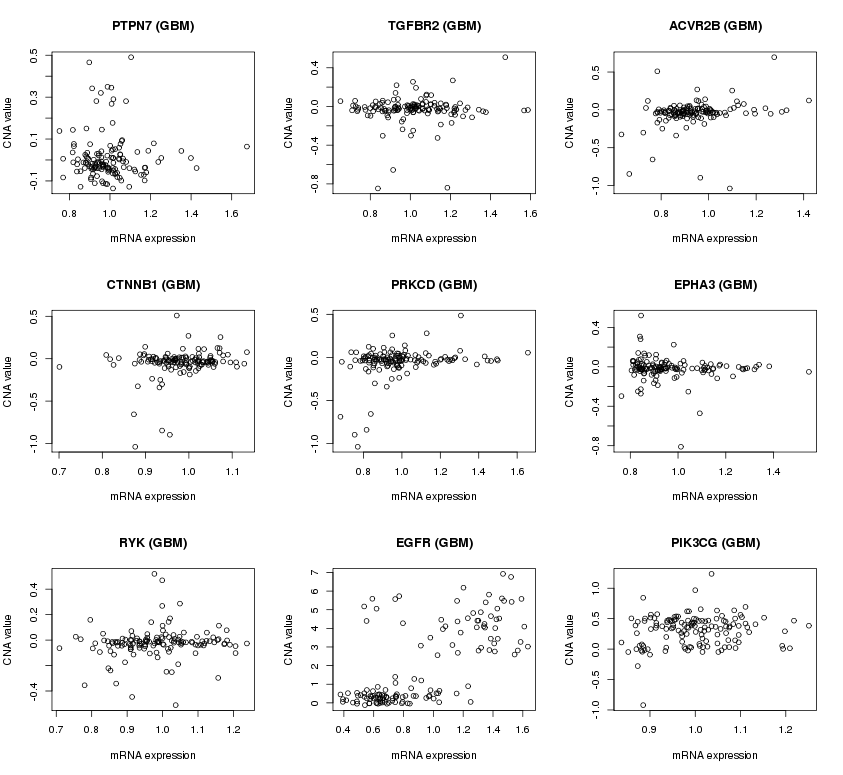


D


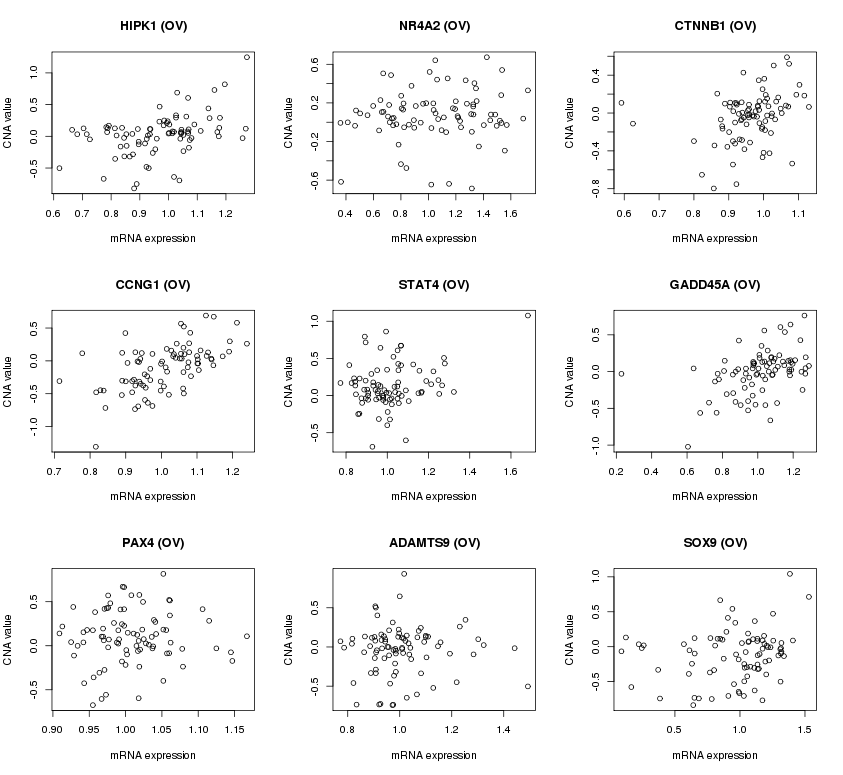


Figure S4. Overlap of predictions by NEA and sequence-based methods.

The agreement between NEA and the three sequence tools was roughly as poor as that between the latter pairwise.

A, mutations in glioblastoma multiforme

B, mutations in ovarian carcinoma

A


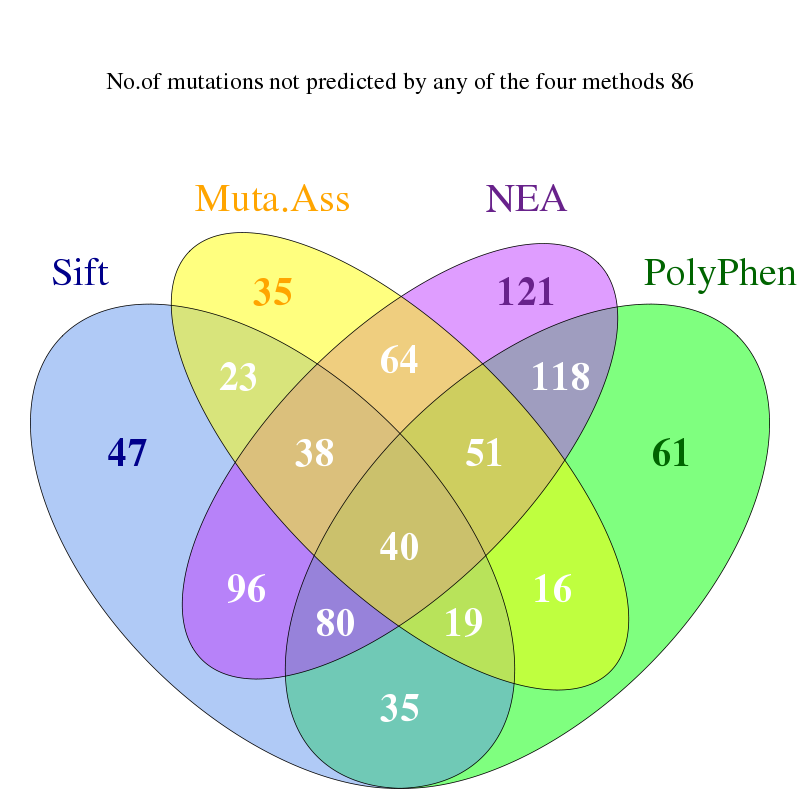


B


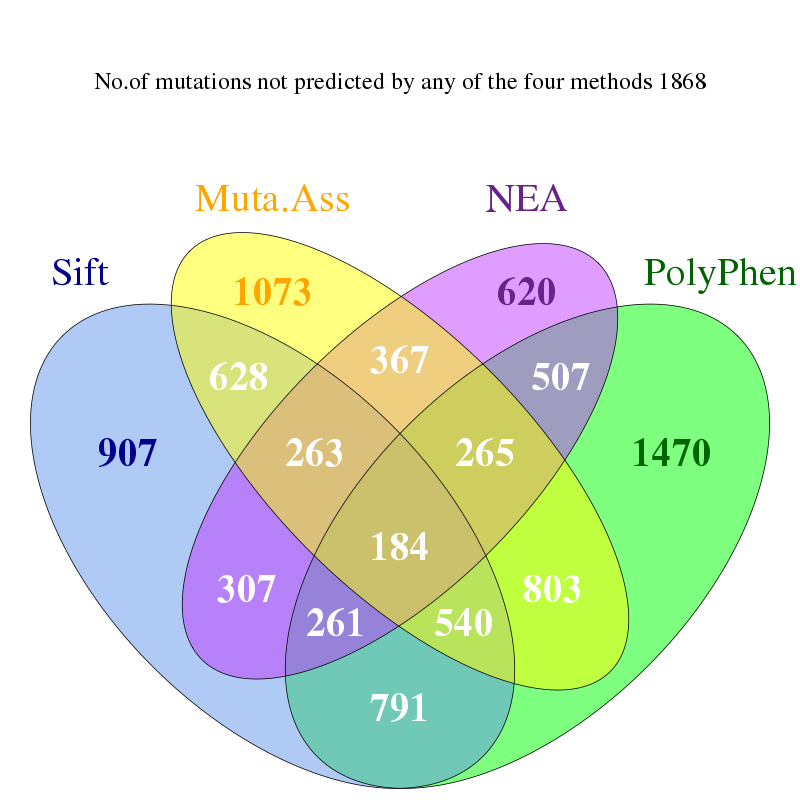


Figure S5. Agreement between silent/nonsense/missense classification of mutations and NEA driver analysis.

We calculated concordance of the two methods as enrichment in tables of the following form:

|  | | P-value from NEA, compared to cut-off *C* | |
| --- | --- | --- | --- |
|  |  | Above | Below |
| Consequence of mutation | missense AND nonsense | *a* | *b* |
|  | silent | *c* | *d* |

The tables were analyzed with Fisher's exact test (Y-axis). Overall, significance of the enrichment grew with stringency of the NEA p-value cut-off *C* (tested in the range 10^-1^...10^-20^, X-axis presents -log10(C)), which indicated presence of signal in the NEA methods and its increase with confidence.


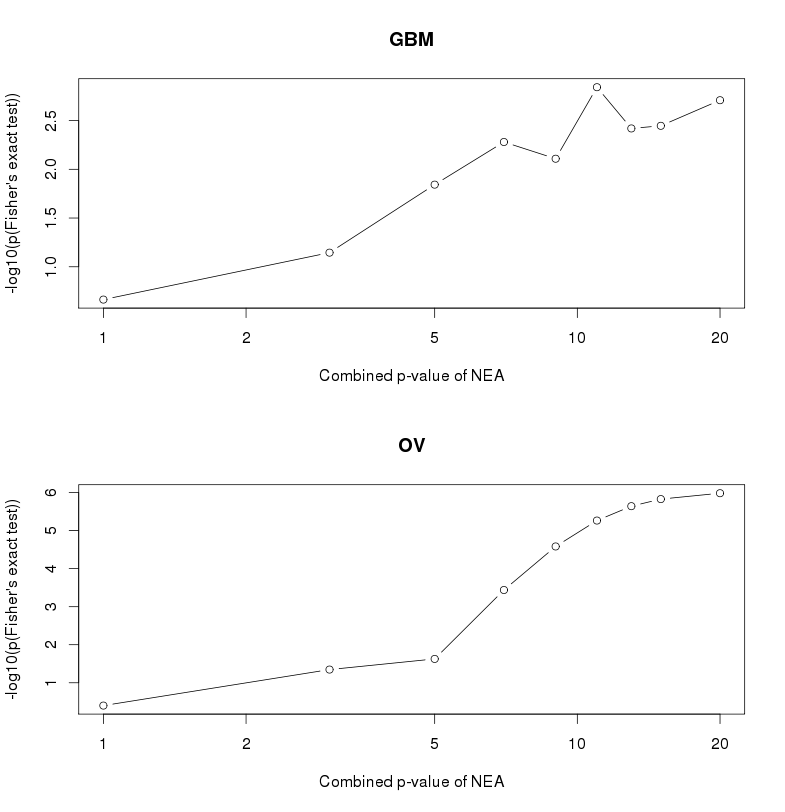


Figure S6. Agreement between driver gene sets from Parsons et al. (2008), Vogelstein et al. (2013) and predictions made with NEA.

A. Each of the three different p-value columns (Passenger Probability Low, Passenger Probability Mid, and Passenger Probability High) from Parsons et al. (2008) were compared to results of the three alternative NEA procedures: cancer pathways (CPW), somatic point mutation gene sets (MGS), and sets of copy number altered genes (CNA) on either GBM or OV dataset. Each comparison of log-transformed p-values (shown as –log10(p) at X and Y axes) was quantified with linear Pearson and Spearman rank correlations, values of which are given below the plots together with N available genes. Despite relatively low values of correlation coefficients, they were always positive (the minimum of 0.138 was observed for MGS in OV, where the analysis was challenged by overwhelming high fractions of passenger mutations).

B. The list of cancer predisposition genes (Table S4 from Vogelstein et al., 2013) was matched to the same NEA estimates as in B. At p-value cut-offs of growing stringency, we calculated concordance of the two methods as enrichment in tables of the following form:

|  | | Found in the cancer predisposition list | |
| --- | --- | --- | --- |
|  |  | Yes | No |
| P-value from NEA, compared to cut-off *C* | <*C* | *a* | *b* |
|  | >*C* | *c* | *d* |

The tables were analyzed with Fisher's exact test and plotted in the left column. Overall, the enrichment significance grew with lowering the p-value cut-off *C* (tested in the range 6*10^-1^...10^-10^), which indicated presence of true positives in the both methods.

A


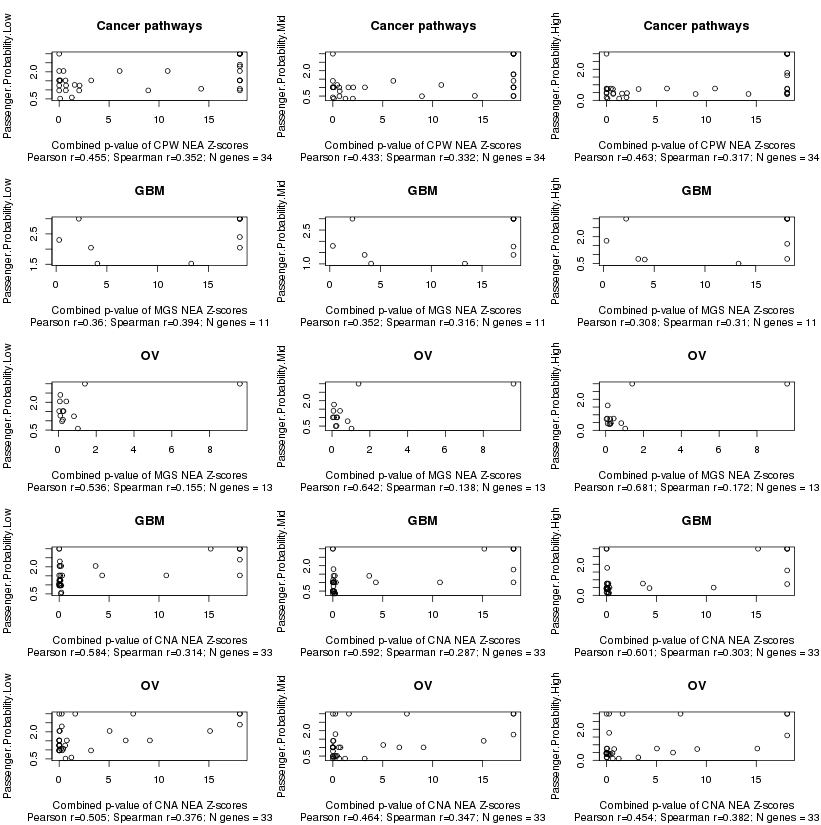


B


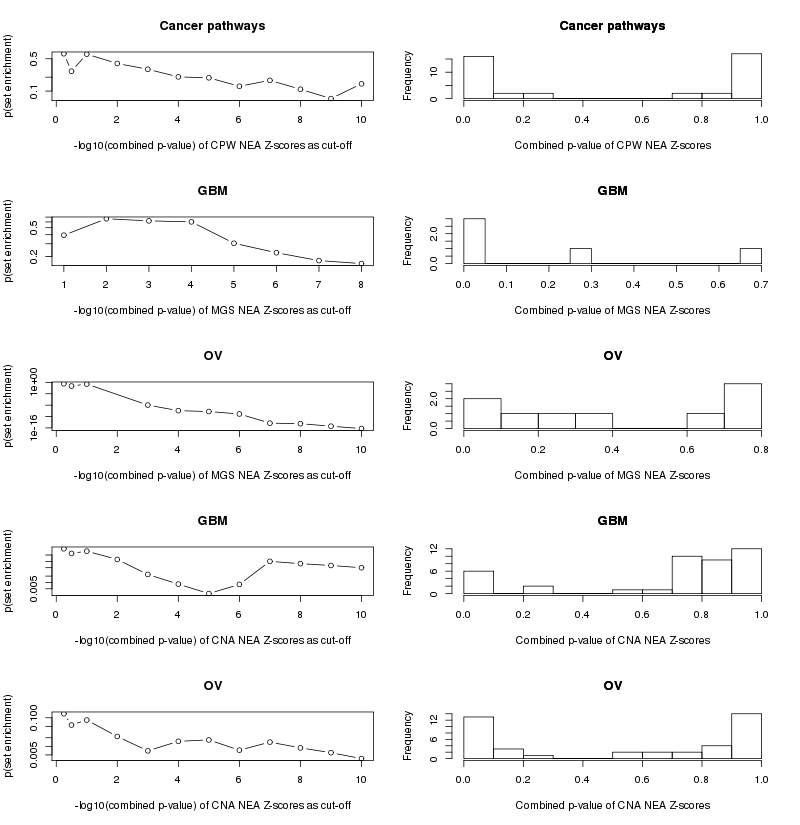


Figure S7. Positive prediction rate of sequence and network methods in mutations of different frequency.

We analyzed 1) all mutations in protein coding genes reported in the GBM and OV sets from TCGA (black); 2) genes Parsons et al. (2008) (green) and 3) genes from Vogelstein et al. (2013) (red). The were binned by their occurrence. The numbers show amounts of distinct genes found in a given number of tumor samples. For example in GBM, 276 genes were found in single samples in total, and 20 genes of these were from the list by Vogelstein et al. (2013). In the sequence analyses, 38% and 39% of these mutations were tested positively (vertical axis). For comparison 13% and 29% of the same sets were tested positively in the network enrichment analysis (NEA FDR<0.1).

Figure S8. Agreement between co-occurrence with other point mutations and results of NEA on mutated gene sets (MGS).

X-axis: NEA z-score obtained in the analysis of a single mutation against the set of other mutations in the same genome (MGS mode).

Y-axis: No. of different other genes with mutation profiles matching that of the given gene by Fisher's exact test (*p_0_* < 0.01).

The red lines show the partitioning at which the significance of association between X and Y values was estimated, i.e. NEA FDR < 0.1 and no. of co-occurring mutations > 4. The partitioning resulted in 2x2 tables which indicated enrichment (binomial Z-test, the Z values shown in the title; *p_0_* = 0.00027 and *p_0_* = 0.000008 for GBM and OV, respectively).


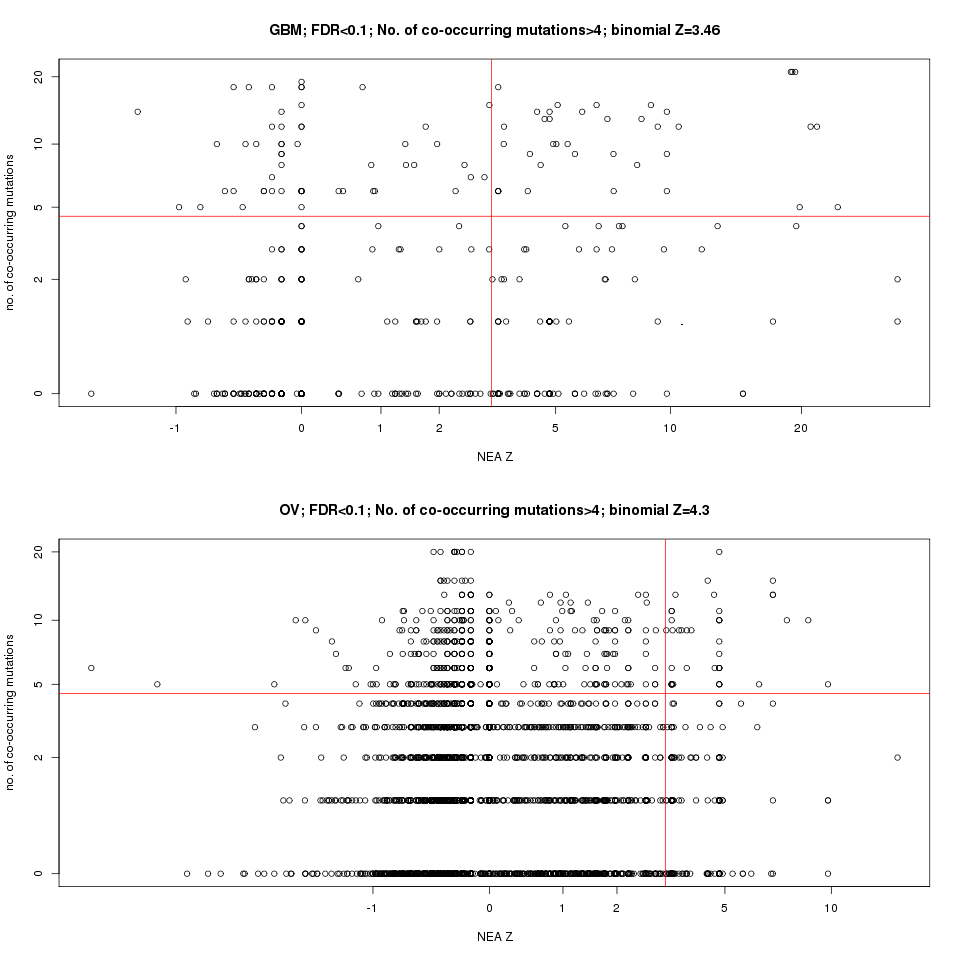

Supplement: Supplementary file 4 — Additional file 4: [Drivers_in_glioblastoma.Supplementary.July29.docx] contains supplementary methods (reverse engineering of network wir1), tables (the descriptions of benchmarked networks and the re-analysis of results from Ciriello et al. [ [29]]) as well as the supplementary figures. (DOCX 744 KB) [file 12859_2014_6590_MOESM4_ESM.docx]
